# Photoactivatable and photolabile pharmacophores: lessons learned from capsaicin†

**DOI:** 10.1039/d5cb00124b

**Published:** 2025-07-19

**Authors:** Nils Imse, Lucia Rojas, Cristina Gil Herrero, Sebastian Thallmair, JeongSeop Rhee, Nadja A. Simeth

**Affiliations:** a Institute for Organic and Biomolecular Chemistry, Department of Chemistry, University of Göttingen Tammannstr. 2 37077 Göttingen Germany nadja.simeth@uni-goettingen.de; b Max Planck Institute for Multidisciplinary Sciences Hermann-Rein-Str. 3 37075 Göttingen Germany; c Frankfurt Institute for Advanced Studies Ruth-Moufang-Str. 1 60438 Frankfurt am Main Germany; d Faculty of Biochemistry, Chemistry and Pharmacy, Goethe University Frankfurt 60438 Frankfurt am Main Germany; e Cluster of Excellence “Multiscale Bioimaging: from Molecular Machines to Networks of Excitable Cells” (MBExC), University of Göttingen 37075 Göttingen Germany; f Department of Chemistry – Ångström laboratory, Uppsala University Box 523, 751 20 Uppsala Sweden

## Abstract

Light-controlled molecules have become valuable tools for studying biological systems offering an unparalleled control in space and time. Specifically, the remote-controllable (de)activation of small molecules is attractive both to study molecular processes from a fundamental point of view and to develop future precision therapeutics. While pronounced changes through light-induced cleavage of photolabile protecting groups and the accompanying liberation of bioactive small molecules have become a highly successful strategy, approaches that focus solely on the revert process, *i.e.* the photochemical deactivation of bioactive agents, are sparse. In this work, we studied whether a given bioactive compound could be made photolability by structural design. We thus used the example of capsaicinoids, which control the transient receptor potential cation channel subfamily V member 1 (TRPV1), to generate both suitable light activation and deactivation strategies.

## Abstract 2

Structurally merging light-susceptible with bioactive molecules allows to generate photolabile chromopharmacophores. Using capsaicinoids, chromopharmacophores could activate the TRPV1 channel, while no activation is observed for the photoproducts.

## Graphical abstract

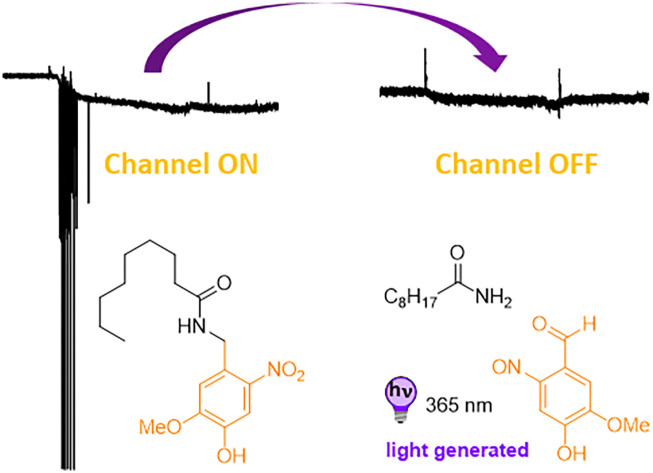

## Introduction

**Fig. 1:**
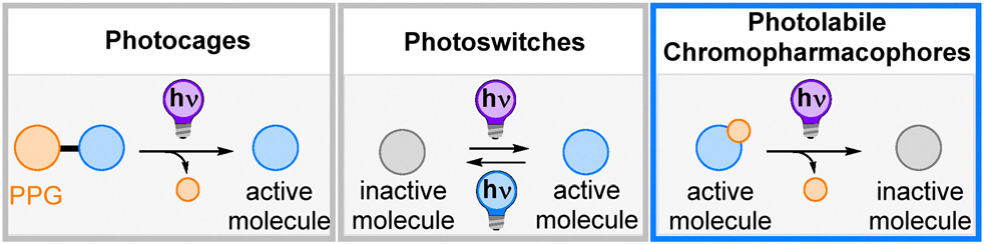
Representation of different strategies for light-control of bioactive molecules.

Light-controlled tools have emerged as indispensable probes to study and manipulate biological systems over the past decades.^1–3^ Diversification in photoresponsive fusion proteins gave rise to more and more applicable optogenetic tools with uses ranging from fundamental research to the clinic with some constructs having entered trials towards seeing or hearing restauration, for example.^4–7^ On the other end of the spectrum, small molecule photoactuators have been used as artificial building blocks for biomacromolecules, agonists and antagonists for protein receptors, enzyme inhibitors, nucleic acid intercalators, and neuronal signaling agents, to name only a few examples.^2,8–11^ The molecular tools are divided into photoswitches^2,12^ and photocages, *i.e.* photolabile protecting groups (PPGs).^13,14^ While photoswitches are molecular motifs that can reversibly isomerize between two forms and thus switch on and off biological activity on demand, photocages mostly act as steric blocking groups to prevent a biological response until light-induced bond cleavage from the biomolecule during uncaging (Fig. 1).^1^ The use of photoswitches is advantageous due to the reversibility of the biological response they induce. However, it is not trivial to design a switchable photopharmacophore in such a way to show complete isomerization and high differences in biological activity between the two forms.^2^ Photochemical uncaging is an irreversible process that usually results in very high differences in biological activity as typically a tailored bioactive molecule is used and simply temporarily blocked through a chemical modification with the PPG. On the downside, the so-released molecule cannot be deactivated anymore, which can be a disadvantage if prolonged exposure to the pharmacophore can lead to discomfort, side effects, or potential loss of drug-ability of the biological target.^15^ Examples include antibacterial agents, which can teach microbes to develop resistance over prolonged exposure,^16^ anticancer drugs, which induce cell damage also to healthy cells,^17^ and also a simple capsaicin-based warming lotion that can cause burning sensation or harm to the skin after longer periods of treatment or at higher concentrations.^18^

Thus, switching off biological activity beyond the spatiotemporal application range would be advantageous for patient treatments, in particular if a high difference in biological activity (on → off) could be achieved, comparable to photocages. This property profile could be realized if the photocage would be simultaneously biologically active and then deactivated with light, *i.e.* becomes a photolabile chromopharmacophore. From a molecular design perspective, this requires merging chemical motifs, that respond to light in a bond-cleavage reaction, with a biologically active motif. This is challenging as these structures are not necessarily similar. As a result, only a few examples are known to date. For instance, the competitive inhibitor dl-*threo*-β-benzyloxyaspartic acid was transferred into its photolabile analogue by introducing a NO_2_-group in the *ortho*-position to the benzylic ether. This compound showed inhibitory activity, while hydroxyaspartate, which was obtained as a photochemical degradation product, was inactive.^19^ Already in 2000, a similar approach was applied to construct a light-deactivatable antibiotic.^20^ In this case, the cleavage of the PPG was used to initiate a cascade reaction that led to degradation after irradiation.^20,21^ More recently, a puromycin analog was modified with two PPGs, one to activate and the second one to deactivate the pharmacophore.^22^

**Fig. 2:**
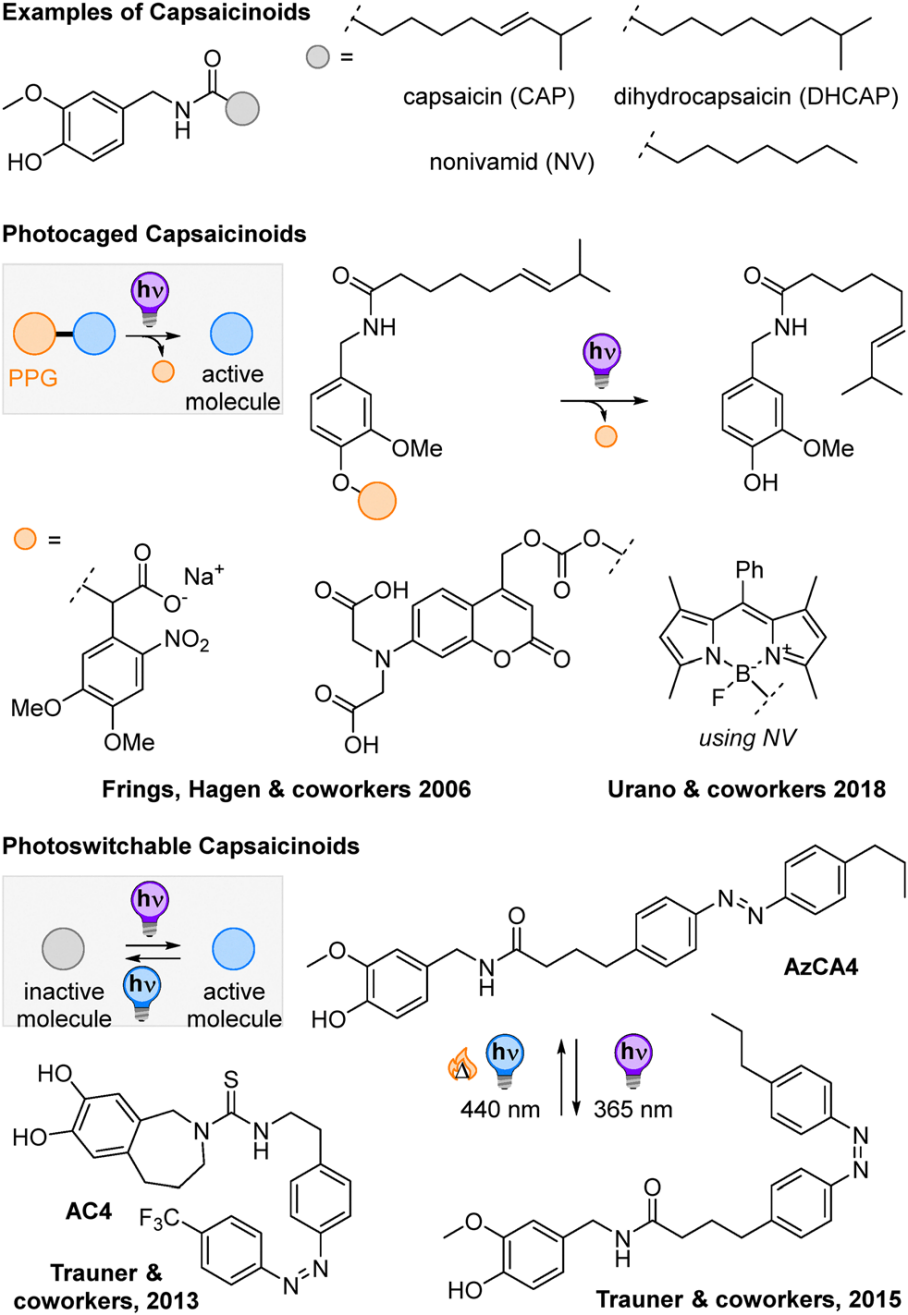
Chemical structures of capsaicinoids and previously developed light-responsive variants employing both PPGs and photoswitches.^23–26^

In this work, we explored whether it is possible to identify a general design strategy to transfer bioactive compounds into photolabile chromopharmacophores. To do so, we targeted the transient receptor potential cation channel subfamily V member 1 (TRPV1), which is responsible for pain sensation and responds to small molecules, such as capsaicin (Fig. 2). The channel is not only crucial in signal transduction between neurons, but was also targeted before *via* photoactivatable^23,24^ and photoswitchable ligands,^25,26^ and thus appeared to be a reliable starting point for our studies.

Frings, Hagen, and coworkers had prepared α-carboxy-4,5-dimethoxy-2-nitrobenzyl-caged capsaicin in 2006 and could show efficient release of capsaicin through 334 or 365 nm irradiation in aqueous buffer with a quantum yield *Φ* of 5%.^23^ Using BODIPYs instead of *ortho*-nitrobenzenes (ONBs) as photocages, Urano and coworkers could move the wavelength of irradiation to 500 nm; however, the uncaging quantum yield was lower (*Φ* = 0.21%, methanol).^24^ Due to the difference in molar extinction coefficient ε, the uncaging action cross-section (*εΦ*_u_) of both photocaged compounds was in the same order of magnitude (134 M^−1^ cm^−1^ for the BODIPY and 235 M^−1^ cm^−1^ for the ONB-caged compounds), sufficient for TRPV1 activation in living cells through light.^23,24^ While both examples show potential as light-controlled pharmacophores, they cannot be deactivated anymore, once the photocage is cleaved off.

In contrast, the photoswitchable agonists and antagonists developed by Trauner and coworkers in 2013 and 2015 (Fig. 2, bottom part) are photocontrolled *via* the reversible isomerization about an N=N double bond.^25,26^ In this manner, they can be toggled between an active and an inactive form. Photoswitching is performed with 365 and 440 nm light, respectively. The antagonist AC4 was active in the *E* form and could be deactivated upon 365 nm light-induced *E* → *Z* isomerization, while the agonist AzCA4 showed TRPV1 activity under UV-light illumination. The extend of channel current could be coupled to the photoisomer ratio and thus adjusted by tuning the illumination wavelength. Both compound series show high potential for applications; however, not all derivatives investigated showed the same behavior making photoswitchable pharmacophores less predictable than the photocages. Moreover, the change between the active and the inactive form is generally less pronounced, so that some background activity might be observed (*ca.* 20% in AC4).

These previous studies showed that pronounced activation could be realized through photocages, and significant levels of reversibility could be achieved through photoswitches. In the current study, we will investigate, if photolability could lead to pronounced changes in activity, however, through deactivation.

## Results and discussion

### Design and synthesis

**Scheme 1:**
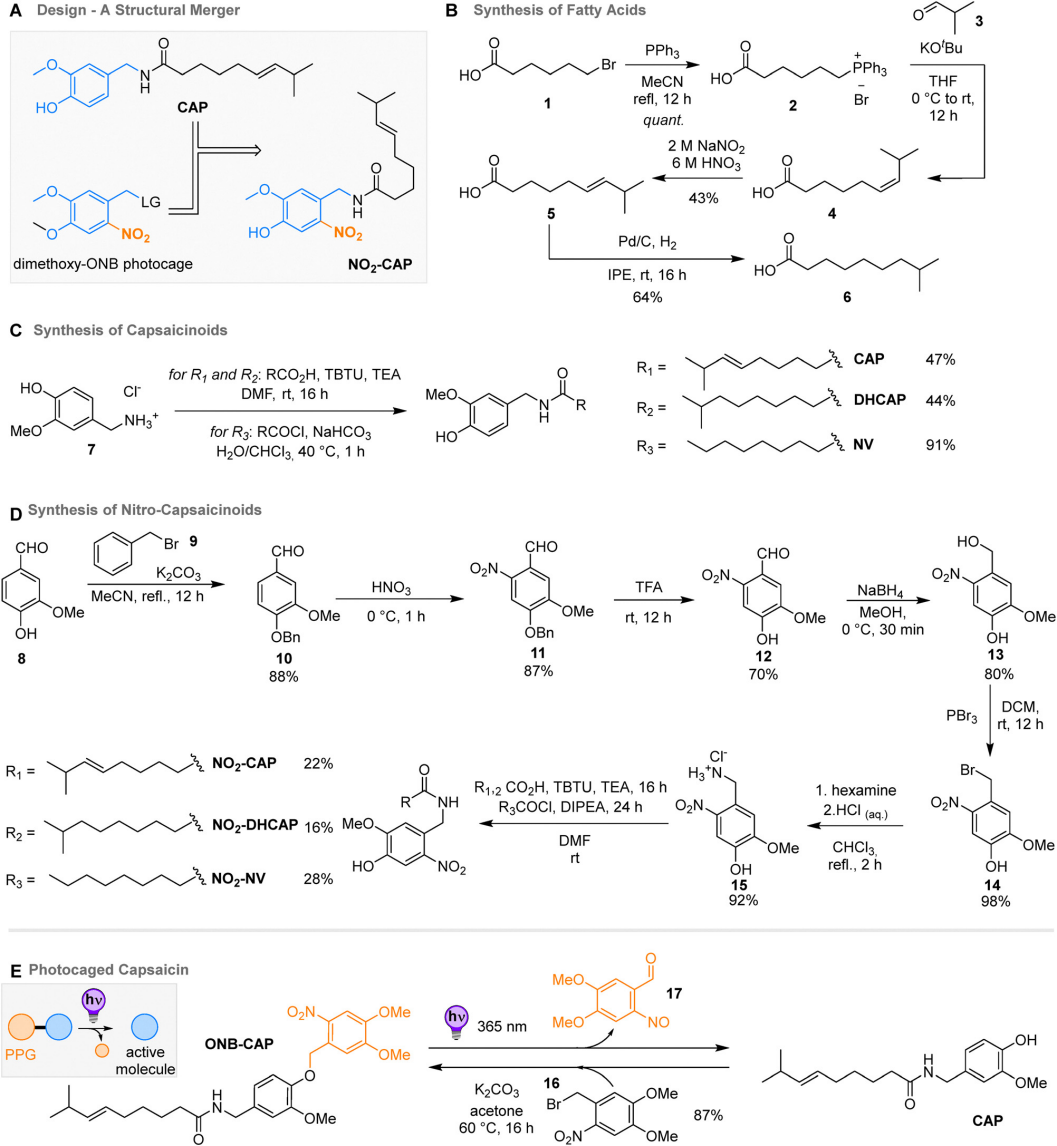
Structural merger of capsaicin (CAP) and *ortho*-nitrobenzene (ONB) and caged capsaicin ONB-CAP. (A) Design of the photolabile chromopharmacophores. (B) Synthesis of different fatty acids to serve as lipophilic side chains of ((nitro)-capsaicinoids). (C) Chemical synthesis of (nitro)capsaicinoids. (D) Synthesis of the capsaicinoids CAP, DHCAP, and NV (top), and their light-sensitive derivatives NO_2_-CAP, NO_2_-DHCAP, and NO_2_-CAP (bottom). (E) Synthesis of caged capsaicin ONB-CAP, and the photoinduced uncaging reaction.

We selected capsaicin as a suitable target structure to become a photolabile chromopharmacophore as it shares a structural core motif with known ONB photocages (Scheme 1(A)). Indeed, both molecules belong to the vanilloid family with the key difference that ONB bears a NO_2_-group next to the benzylic position, which is crucial for its sensitivity towards light. Thus, we assumed that introducing a NO_2_-group on the aromatic core of capsaicin in the *ortho*-position to the lipid chain would render the pharmacophore light-sensitive and photolabile. We hypothesized that this minor structural change would still result in TRPV1 binding, but expected a decreased effect compared to capsaicin or even an agonist behavior.^27^

### Synthesis of lipid chains

For the synthesis of the lipid side chains for capsaicin (CAP) and dihydrocapsaicin (DHCAP), *i.e.* (*E*)-8-methylnon-6-enoic acid 5 and 8-methylnonanoic acid 6, a Wittig reaction was chosen as the key step (Scheme 1(B)).^28^

Firstly, 6-bromohexanoic acid 1 was made to react with triphenyl phosphine to obtain 2 in quantitative yields. In the next step, the phosphonium salt was treated with potassium *tert*-butoxide as the base and iso-butyraldehyde to form 3 in the *Z*-conformation.^28^ Without purification, the crude product was converted to the corresponding *E*-isomer by heating it in an aqueous solution of 6 M nitric acid and 2 M sodium nitrite. In this way, compound 5 was obtained in 43% yield over two steps as a mixture of conformers (*E* : *Z* 4 : 1, based on NMR analysis). Hydrogenation of 5 with palladium on carbon resulted in 8-methylnonanoic acid 6 in a 64% yield.

### Synthesis of capsaicinoids

The synthesis of CAP and DHCAP started from commercially available vanillyl amine hydrochloride 7, which reacted with the respective carboxylic acids to attach the lipid side chains 5 and 6 for CAP and DHCAP in the presence of the coupling agents TBTU and triethylamine (47% and 44%, respectively, Scheme 1(C)) adapting a reported procedure.^29^ Capsaicinoid nonivamide (NV) was obtained by acetylating 7 with pelargonyl chloride in high yields (91%).^30^

### Synthesis of nitro-capsaicinoids

For the synthesis of the nitro-analogues, we started from vanillin 9 and protected the phenolic OH with a Bn group, using benzyl bromide, to result in 10 (88% yield, Scheme 1(D)). Afterwards, 10 was nitrated with nitric acid to yield 11 (87%), which was then benzyl-deprotected with TFA (70% yield).^31^ The aldehyde group of the so-obtained 12 was then reduced to the corresponding alcohol 13, using sodium borohydride (80% yield) and subsequently brominated in an Appel reaction with phosphorous tribromide giving 14 in 98% yield. Then, 14 was converted with hexamine into the primary amine 15 in a Delepine reaction (92% yield). The nitro-derivatives of CAP and DHCAP were formed by reacting the respective carboxylic acid side chains with TBTU and triethylamine (22% yield for NO_2_-CAP and 16% for NO_2_-DHCAP) and NO_2_-NV was synthesized using pelargonyl chloride and DIPEA (28% yield) and confirmed by X-ray analysis (see the ESI,† Section S6).

### Synthesis of photocaged (nitro-)capsaicinoids and photoproducts

To better compare the impact of light-irradiation on our light-sensitive molecules and their photochemically generated by-products in *in cellulo* experiments on TRPV1 (de)activation, we synthesized the photocaged capsaicin ONB-CAP (Scheme 1(E)). The molecule was obtained by reacting CAP with ONB bromide 16 (87% yield).

**Fig. 3:**
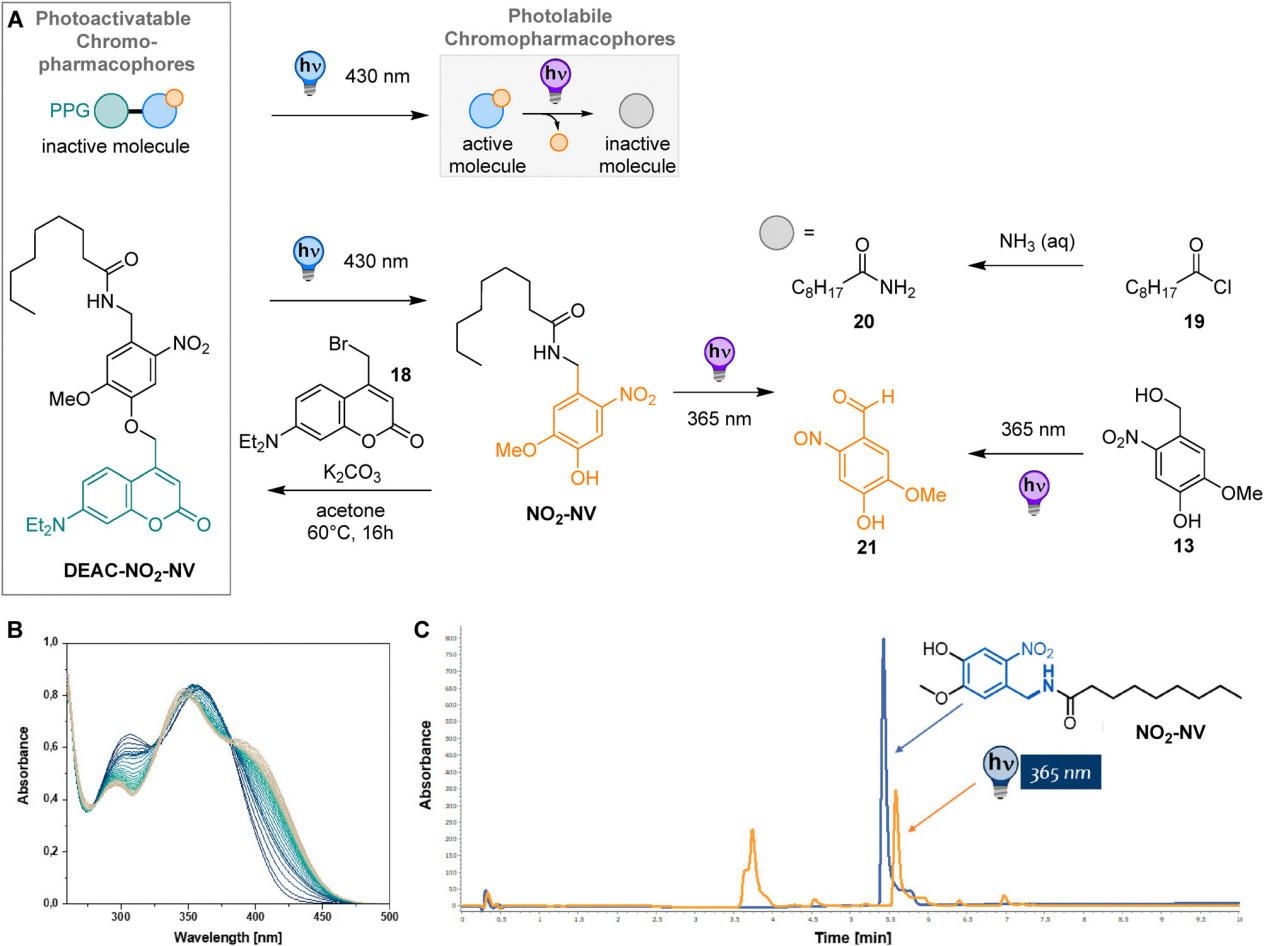
Synthesis and analysis of photoresponsive NO_2_-NV derivatives and its photoproducts. (A) Synthesis of photocaged nitro-nonivamide to serve as a two-PPGs-one-molecule (TPOM) for two-step light-induced activation and deactivation, as well as synthesis of reference compounds 20 and 21, and photochemical reactions of the various components. (B) Time-resolved UV-Vis absorption spectrum of NO_2_-NV in MeOH (50 μM) during irradiation with 365 nm light. (C) HPLC-traces of NO_2_-NV before (blue) and after irradiation (orange) with 365 nm light (a transparent HPLC vial was irradiated for *ca.* 60 min with a 365 nm LED to insure the completion of the reaction; photoproducts of the ONB-core eluted between 3.5 and 4 min, nonanamide at *ca.* 5.6 min; traces recorded at 220 nm in a gradient of 5 → 95% MeCN in water with 0.1% formic acid; for more details see the ESI,† Section S3).

We additionally synthesized the expected photocleavage products 20 and 21 as controls (Fig. 3(A)) to understand their activity towards TRPV1 (*vide infra*). This is of crucial importance as the nitroso aldehydes (*i.e.*21) obtained from the photochemical transformation of ONBs are biologically non-innocent. Specifically, both functional groups can react further into azobenzenes and anilines, or could crosslink to amino groups in proteins.^13,32^ Therefore, 20 was prepared by reacting pelargonyl chloride with aqueous ammonia, while 21 was synthesized by irradiating 13 at 365 nm in a 10 mM ethanol solution (the disappearance of the starting material was confirmed by LC–MS, but it was not isolated in the pure form, due to the reactivity of the nitroso group).^33^

Moreover, we used this opportunity to also functionalize NO_2_-NV with a PPG (Fig. 3(A)). By attaching coumarin 18, which is known to respond to a higher wavelength of light compared to ONB, it would be possible to sequentially address the two photoresponsive units and first activate and then possibly deactivate the potential pharmacophore NO_2_-NV. This strategy was previously used by Schwalbe, Wachtveitl, and coworkers for a puromycin analog taking advantage of large structural changes in both light-induced transformations and was dubbed two-PPGs-one-molecule (TPOM).^22^

### Photophysical and photochemical characterisation of nitro-capsaicinoids

Next, we studied the photophysical and the photochemical properties of the nitro-capsaicinoids NO_2_-CAP, NO_2_-DHCAP, and NO_2_-NV, and the photocaged capsaicin ONB-CAP (*cf.*Table 1 and ESI,† Section S3). As expected for ONB-derivatives, the absorption maxima were around 352–360 nm with extinction coefficients between 3.12 and 5.76 × 10^3^ L cm^−1^ mol^−1^ for the nitro-capsaicinoids and 7.01 × 10^3^ L cm^−1^ mol^−1^ for the photocages derivative (DMSO). All four compounds proved to be photosensitive upon irradiation with 365 nm LED light (Fig. 3(B) and ESI,† Section S3) and underwent a photochemical bond cleavage reaction with quantum yields around 2–3% for the nitro-capsaicinoids and around 12% for ONB-CAP, which is higher due to the better leaving group phenol.^34^

**Table 1:** Extinction coefficient, absorbance and quantum yield of nitro-capsaicinoids in DMSO under irradiation at 365 nm

	NO_2_-CAP	NO_2_-DHCAP	NO_2_-NV	ONB-CAP
*λ*_max_ [nm]	358	360	358	352
*ε* a (*λ*_max_)/10^−3^ [L cm^−1^ mol^−1^]	5.30 ± 0.04	5.76 ± 0.11	3.12 ± 0.04	7.01 ± 0.07
*A*_365 nm_ a	1.04 ± 0.009	1.13 ± 0.015	0.629 ± 0.019	0.628 ± 0.035
*Φ* [%]^a^	1.9 ± 0.2	2.0 ± 0.3	3.4 ± 0.4	12.3 ± 2.3

a All measurements were taken independently three times, and as an error, the highest deviation from the average value was taken for all measured variables.

Also, DEAC-NO_2_-NV was studied regarding its propensity to undergo a stepwise photochemical bond cleavage reaction. The molecule was thus first irradiated with 430 nm light, a wavelength that an ONB-type PPG should not respond to.^31,35^ Indeed, LC–MS analysis showed the formation of NO_2_-NV (see the ESI,† Section S3). Switching then to 365 nm light irradiation, we could confirm that NO_2_-NV could be photochemically degraded (Fig. 3(C)).

### Dose–response-studies on TRPV1

**Fig. 4:**
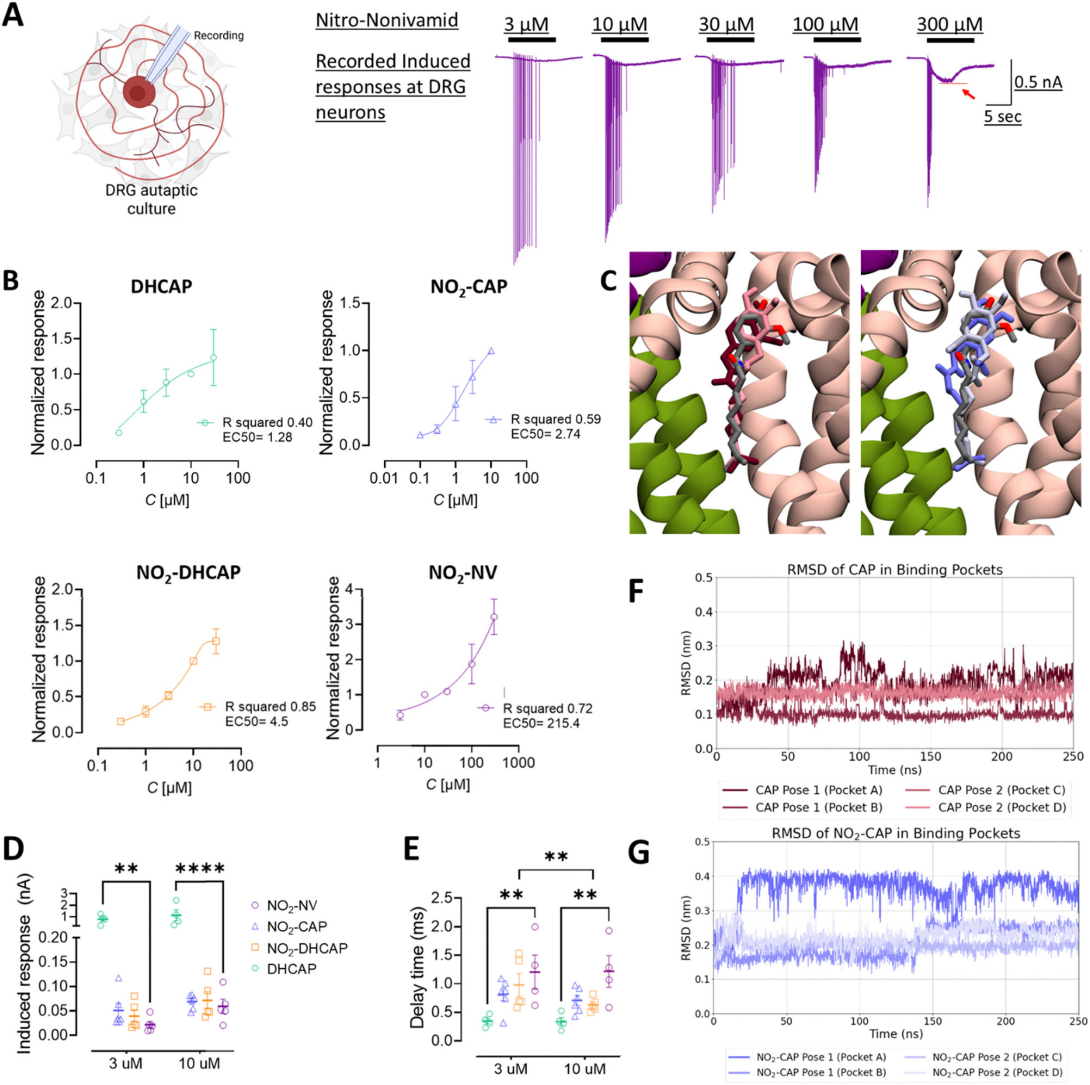
Dose–response relationships and activation kinetics of TRPV1 by capsaicin derivatives in DRG autaptic neurons. (A) Left: A schematic diagram of the autaptic culture DRG neuron. Right: Representative current traces showing responses to various concentrations of NO_2_-NV. The red arrow indicates peak current amplitudes. (B) Dose–response curves were generated by normalizing peak current amplitudes for each compound to their respective response at 10 μM. Data were fit with asymmetric sigmoidal functions. DHCAP (*n* = 4): EC_50_ = 1.28 μM, hill slope = 0.54, and *R*^2^ = 0.40. NO_2_-CAP (*n* = 6): EC_50_ = 2.7 μM, hill slope = 0.62, and *R*^2^ = 0.59. NO_2_-DHCAP (*n* = 5): EC_50_ = 4.5 μM, hill slope = 8.0, and *R*^2^ = 0.85. NO_2_-NV (*n* = 5): EC_50_ = 215 μM, hill slope = 4.3, and *R*^2^ = 0.72. (C) Docking poses of CAP and NO_2_-CAP in TRPV1. Snapshots of the TRPV1 crystal structure (PDB code: 7LPE, chains colored in pink, ice blue and green) displaying CAP from the crystal structure (gray, O atoms in red) and the two binding poses with the highest docking score for CAP (left; dark blue: highest, light blue: second highest docking score) and NO_2_-CAP (right; dark orange: highest, light orange: second highest docking score). (D) Quantification of peak current amplitudes at 3 μM and 10 μM. At both concentrations, DHCAP induced significantly larger responses compared to all the other compounds (one-way ANOVA). Mean ± SEM for 3 μM/10 μM: NO_2_-NV: 0.022 ± 0.004/0.059 ± 0.014, NO_2_-CAP: 0.051 ± 0.014/0.069 ± 0.006, NO_2_-DHCAP: 0.039 ± 0.071/0.071 ± 0.018, and DHCAP: 0.78 ± 0.205/1.13 ± 0.47. (E) Latency from ligand perfusion to the response onset. NO_2_-NV elicited significantly slower responses than DHCAP at both concentrations. Mean onset (s) ± SEM for 3 μM/10 μM. NO_2_-NV: 1.2 ± 0.6/1.2 ± 0.5, NO_2_-CAP: 0.8 ± 0.2/0.7 ± 0.21, NO_2_-DHCAP: 0.97 ± 0.45/0.63 ± 0.12, and DHCAP: 0.35 ± 0.10/0.34 ± 0.14. (F) and (G) Atomistic MD simulations of CAP/NO_2_-CAP in TRPV1 binding pockets. The RMSD of CAP (F) and NO_2_-CAP (G) depicted for the 250 ns simulation time.

To evaluate the potency and activation kinetics of capsaicin derivatives on TRPV1, we performed whole-cell voltage clamp recordings in autaptic culture dorsal root ganglion (DRG) neurons, which endogenously express functional TRPV1 receptors. This preparation allows for the direct measurement of ligand-induced currents following TRPV1 activation by exogeneous application of the capsaicin derivatives (Fig. 4(A)).

Dose–response curves were established for four capsaicin derivatives, *i.e.*DHCAP, NO_2_-CAP, NO_2_-DHCAP, and NO_2_-NV, on TRPV1-mediated currents recorded in DRG neurons (Fig. 4(B)). Despite limited numbers *n* affecting the precision of curve fitting, comparative analysis of the dose–response relationships consistently revealed a shift of EC_50_ values to higher concentrations following the introduction of a nitro group.

This trend was observed for both NO_2_-CAP and NO_2_-DHCAP, and was most pronounced for NO_2_-NV, which required concentrations of up to 300 μM to elicit measurable responses and exhibited a substantially elevated EC_50_ value of 215.4 μM. In contrast, DHCAP elicited significantly larger peak currents than any of the nitro-containing derivatives (Fig. 4(D)).

To further characterize TRPV1 activation kinetics, we measured the latency from compound perfusion to the onset of induced inward current (Fig. 4(D)). Among the tested compounds, DHCAP induced the most rapid activation, whereas all nitro-containing derivatives displayed significantly delayed response onsets. This kinetic lag, together with reduced efficacy, may reflect a lower binding affinity for TRPV1, potentially resulting in transient or partial channel activation and rapid dissociation from the channel, thereby producing smaller and more transient currents. Taken together, the modifications presented on the derivatives show a reduction in TRPV1 binding compared with the previously documented activation kinetics by capsaicin.^36^

### Docking and molecular dynamics simulations

To gain insights into the impact of the NO_2_ group on the binding pose and its stability, we performed an *in silico* structural investigation – namely docking and molecular dynamics simulations. To this end, we first compared the docking results for the two ligands CAP and NO_2_-CAP. For each ligand, the two binding poses with the highest docking scores are depicted in Fig. 4(C). The structures of CAP show good agreement with the CAP binding pose in the crystal structure (Fig. 4(C), left). The NO_2_-CAP binding poses exhibit a comparable positioning of the aromatic ring and the aliphatic tail, respectively, with respect to the CAP pose in the crystal structure (Fig. 4(C), right). However, stronger deviations are clearly visible compared to the docked poses of CAP. As expected, these deviations are predominantly located around the NO_2_ substituent. The successful docking of NO_2_-CAP into the TRPV1 binding pocket supports the experimental observation that the NO_2_ substituent does not fully impede binding to TRPV1.

To gain further insight into the stability of the docked poses, we performed all-atom molecular dynamics (MD) simulations using the CHARMM36m force field.^37^ Each of the two poses was positioned in two binding pockets of the tetrameric TRPV1 structure, and two separate simulations were performed – one for CAP and one for NO_2_-CAP. Fig. 4(F) and (G) show the RMSD of the ligands in their binding pocket calculated with respect to the docked pose after fitting the system to the residues of the respective binding pocket (for details see the ESI†). The ligands show an RMSD in the range of 0.1–0.4 nm. While the RMSD for CAP is mostly between 0.1 and 0.25 nm with an occasional value of 0.3 nm for one replica, and the RMSD of NO_2_-CAP is between 0.15 and 0.4 nm. Fig. S33 in the ESI† provides a visual impression of the different RMSD observed for the two ligands. While, for CAP, the positioning of the ligand remains close to the crystal structure, NO_2_-CAP shows higher mobility and in two cases, the aromatic ring leaves its binding position. This indicates reduced stability and might be an early step towards ligand unbinding, which is expected to occur beyond the time window of 250 ns. Overall, our all-atom MD simulations show that CAP remains closer to its docked pose and exhibits a higher stability in the binding pocket than NO_2_-CAP.

### Illumination of (nitro-)capsaicinoids on DRG neurons and DRG–spinal cord neuron co-culture models

While *in operando* UV-Vis absorption spectroscopy under illumination and HPLC analysis of the samples revealed the successful photochemical bond cleavage of the ONB and DEAC motifs in or on (nitro)capsaicinoids, we now wanted to study whether these effects could be translated to TRPV1 responses in living neurons.

**Fig. 5:**
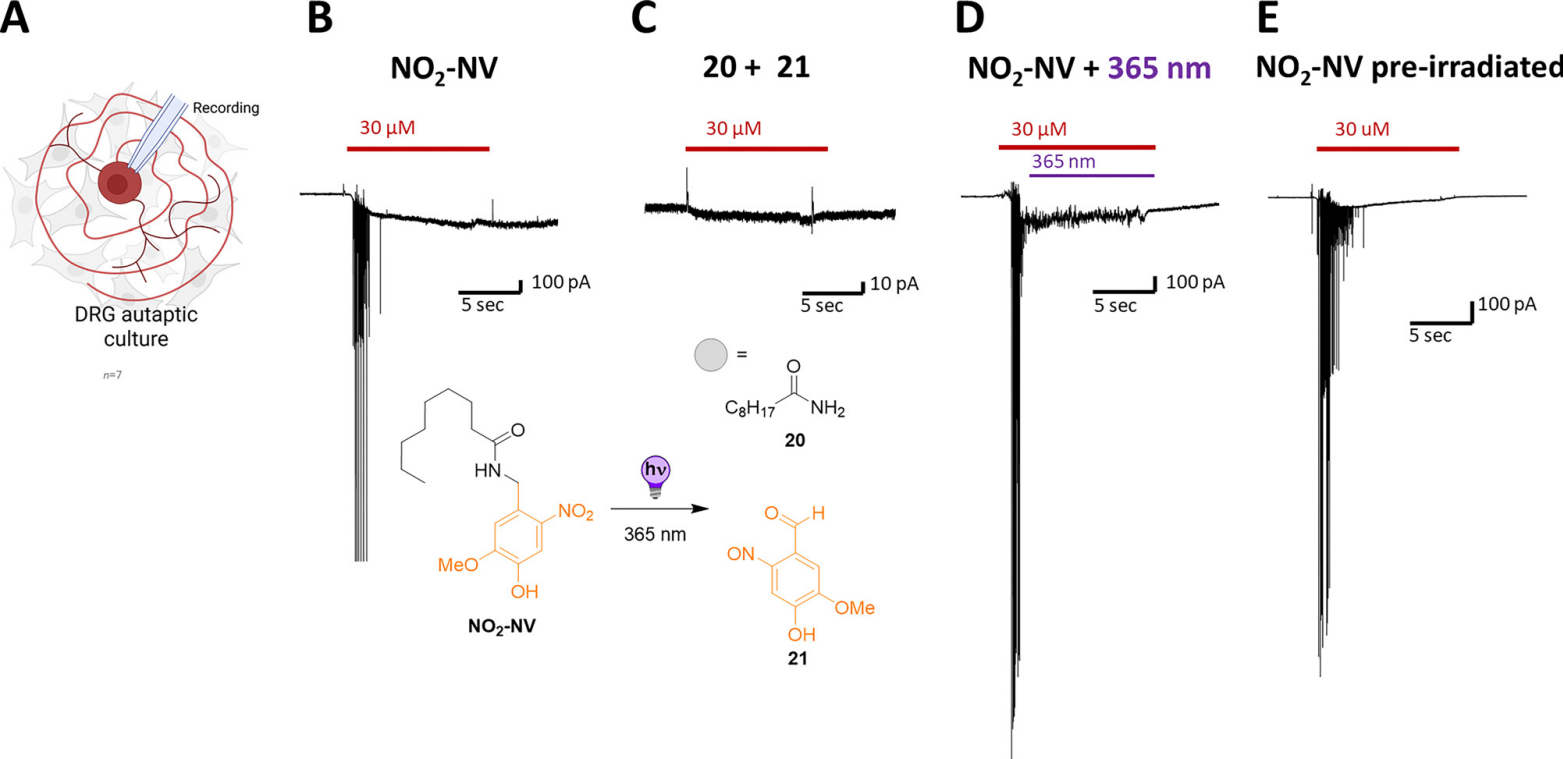
Light-induced modulation of TRPV1 activation by NO_2_-NV in DRG neurons. (A) Schematic diagram of the autaptic culture DRG neuron. (B) Representative trace of the response evoked by NO_2_-NV in the absence of illumination. (C) A mixture of 20 and 21, expected photolysis products, was applied as additional negative control and failed to induce any measurable currents. (D) Representative trace of the NO_2_-NV-induced response in the presence of light. (E) Representative trace of the response to previously irradiated NO_2_-NV used as a negative control; currents persisted despite prior NO_2_-NV inactivation.

First, we examined whether activation of NO_2_-NV responses in DRG neurons (Fig. 5(A)) could be affected by 365 nm light. Thus, we compared the response of NO_2_-NV (Fig. 5(B)) with the predicted photolysis products, 20 and 21 (*vide supra*). As expected, these compounds failed to induce any measurable response (Fig. 5(C)), supporting the idea that these cleaved fragments lack TRPV1 agonist activity and do not have any negative implications on the channel.

Next, we aimed for the *in situ* control of DRG neurons by light. Surprisingly, illumination during drug application continued to elicit robust responses in DRG neurons (Fig. 5(D) and ESI,† Section S5). Furthermore, NO_2_-NV pre-irradiated persisted with 365 nm light (Fig. 5(E)) and retained the response to activate neurons, indicating that small amounts of NO_2_-NV potentially left in solution after irradiation were still sufficient to activate the highly sensitive TRPV1. While LC–MS and ^1^H-NMR analyses indicated quantitative transformation into the photoproducts, the experiment underlined the low conversion rates measured for this compound, pointing to the possibility of leftover NO_2_-NV present during the comparably shorter irradiation times on neurons (*cf.* ESI,† Section S3).

**Fig. 6:**
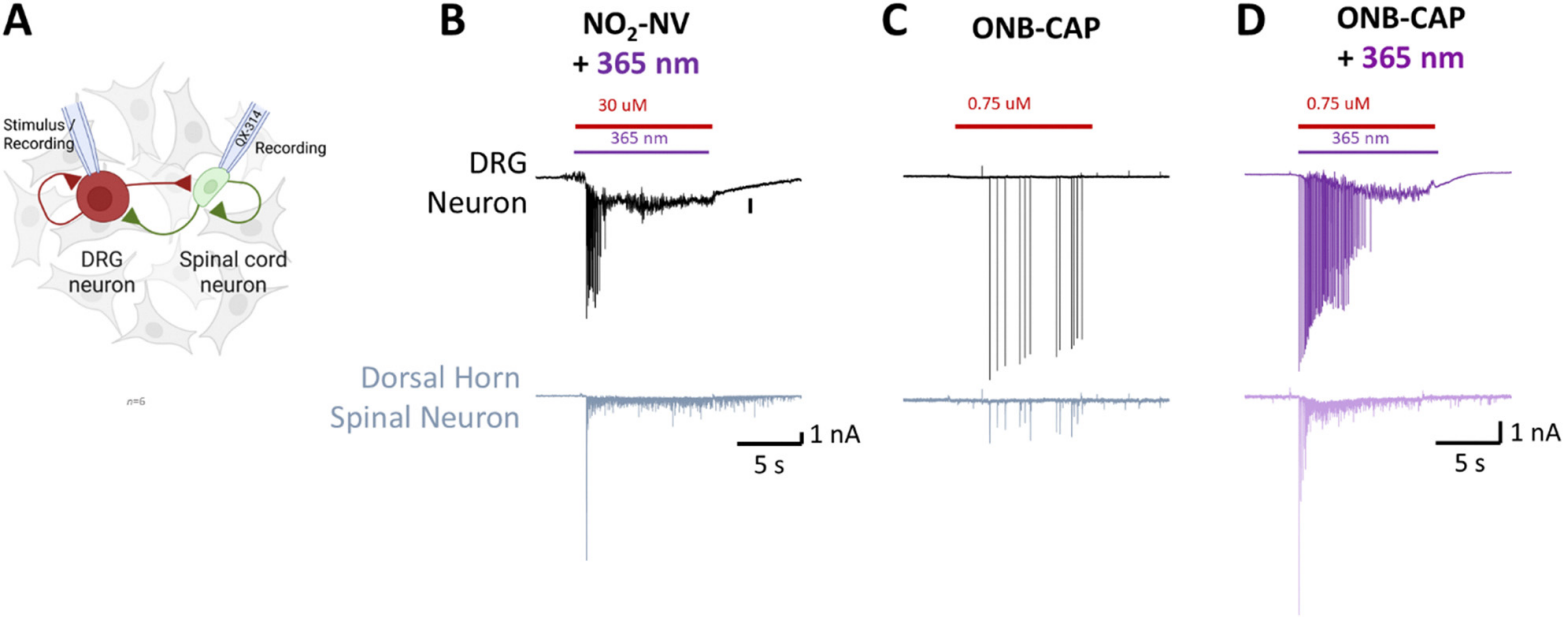
Functional assessment of NO_2_-NV photosensitivity in a DRG–spinal cord neuron co-culture model. (A) Simultaneous dual whole-cell voltage-clamp recordings were performed from DRG and spinal cord neurons co-cultured on a single astrocyte microisland. To prevent action potential firing in the postsynaptic neuron and isolate presynaptic responses from the DRG response, QX-314 was included in the spinal cord pipette solution. (B) Local perfusion of NO_2_-NV induced fast spike currents recorded in both the DRG and the spinal cord neurons, suggesting synaptic transmission. (C) Application of photocaged capsaicin ONB-CAP in the absence of light did not evoke any measurable inward response. (D) Following 365 nm illumination, ONB-CAP triggered currents in both the DRG and spinal cord neurons, indicating light-dependent TRPV1 activation in DRG and subsequent synaptic transmission to the spinal cord.

To investigate the potential photosensitive properties of NO_2_-NV within a synaptically connected system, we next employed a DRG–spinal cord co-culture system combined with dual whole-cell voltage-clamp recordings (Fig. 6(A)). Local application of NO_2_-NV triggered a robust inward current in both DRGs and their postsynaptic spinal cord partners, consistent with synaptic transmission (Fig. 6(B)). Comparable behavior was found for NO_2_-CAP and NO_2_-DHCAP (see the ESI,† Section S5). In contrast, application of photocaged ONB-CAP failed to induce any response in the absence of light. As intended, upon 365 nm illumination, the uncaged compound produced robust currents in both pre- and postsynaptic neurons, indicating light-dependent TRPV1 activation and subsequent synaptic signaling (Fig. 6(C) and (D)). Taking together, we could show in our experiments that the photoproducts 20 and 21 do not trigger DRG neurons, while light-activation of photocaged ONB-CAP showed light-controllable signal transduction between the DRG and spinal cord neurons.

In the case of DEAC-NO_2_-NV and its envisioned role to act as TPOM, the activation step appears plausible; the deactivation, however, can currently not be realized due the inefficient cleavage of nitro-capsaicinoids into their inactive fragments.

## Conclusions and outlook

In this work, we studied whether the introduction of a nitro-group in the *ortho*-position to a benzylic C–X bond can be used as a general strategy to generate photolabile pharmacophores, using the example of capsaicinoids, which act on the transient receptor potential cation channel subfamily V member 1 (TRPV1). We synthesized three different photolabile chromopharmacophores, *i.e.*, NO_2_-CAP, NO_2_-DHCAP, and NO_2_-NV, as well as their parent compounds.

Additionally, we generated the photolabile protected ONB-CAP as a light-responsive control and the photocaged NO_2_-NV and DEAC-NO_2_-NV as potential two-step reversibly activatable molecules. We characterized the light-responsive compounds regarding their photophysical and photochemical behaviours and found that their characteristics were in good agreement with related, literature-known scaffolds underlining the plannable design of such molecules.

The photochemical bond cleavage quantum yields showed strong dependency on the quality of the leaving group, disfavouring cleavage within the nitro-capsaicinoids and favouring the photocaged analogues, including ONB-CAP, which is beneficial for the design of two-step reversible activatable compounds.

Also, LC–MS analysis of light-illuminated samples showed the formation of fragmentized products with a detectable change in size and polarity, which we expected to impact biological activity.

To evaluate the latter, we first performed activity assays using cultured DRG neurons and measured the activation of TRPV1 using *in cellulo* patch clamp experiments. The nitro-capsaicinoids showed a decreased channel response compared to the unmodified parent compound, using DHCAP as an example. Docking and MD simulations underlined this behaviour. Analysing the docking pose of both CAP and NO_2_-CAP, we found a destabilization of the nitrated analogue, which is also reflected in its behaviour over time as revealed by MD simulations.

Next, we investigated whether we see a difference in channel activation between NO_2_-NV and its expected photoproducts and were pleased to see that the latter failed to activate the channel, which speaks for our predicable design of photolabile chromopharmacophores. However, *in situ* irradiation experiments of deactivation did not reflect the same outcome – neither in direct DRG activation, nor in DRG- and DRG–spinal cord neuron co-culture models. We attribute this outcome to two major aspects. On the one hand, the neurons are presented with a large excess of photoresponsive molecules and thus, despite deactivation by light even of the majority of compounds, could leave a sufficient number of active molecules in solution to trigger a channel response. This is supported by a control experiment, in which we first irradiated NO_2_-NV converting most of the molecules into their photoproducts and analysing channel activity, which we could still detect. Secondly, the amide leaving group in NO_2_-capsaicinoids results in a low bond cleavage quantum yield and thus, prolonged irradiation times beyond the timeframe of the channel analysis on neurons are needed to convert the material quantitatively.

From our experiments, we learned that indeed photolabile chromopharmacophores can be rationally designed, when identifying structural mergers of photocleavable motifs and pharmacophores and show a pronounced difference between chromopharmacophores and the photoproducts. Moreover, if an additional PPG can be attached to the structure, a photocaged pharmacophore with a classical off → on response to light can be generated. This approach could pave the way to predictably design two-PPGs-one-molecule systems (TPOMs), such as the here presented DEAC-NO_2_-NV, eventually leading to compounds with off → on → off reactivity.

However, the direct structural merger of ONB and CAP to generate NO_2_-CAP and its analogues limited us in the optimization of the leaving group quality and further structural features potentially improving the sensitivity of NO_2_-CAP to light. These aspects led to a photochemical bond cleavage rate which is too slow to fully unleash the temporal control of light on the time scale of the neuron experiment resulting in too much background activity of the unreacted material to not trigger the highly sensitive TRPV1. On the other hand, our compounds might still be interesting for applications in which the timescale is slow like it is for applications in cremes, in which a thin layer of the compound on the skin surface could be irradiated and deactivated prior to TRPV1 binding. This could help to reduce the effect of overdoses. Once bound to TRPV1, however, irradiation of the current compounds does not result in unbinding and immediate ligand release. Therefore, the photochemical responsivity of the compounds needs to be improved in future work by combining computer-aided drug design with synthesis and biological testing.^38^ We strongly believe that photolabile chromopharmacophores and, based on these, TPOMs can inspire novel approaches in photopharmacology in the future.

## Author contributions

NI and NAS conceptualized the project. NI performed chemical synthesis and photochemical experiments. LR conduced biological testing. CGH and ST performed *in silico* experiments. NAS, JR, and ST supervised the work and acquired funding. All authors contributed to the writing and editing of the manuscript and agreed to the final version.

## Conflicts of interest

There are no conflicts to declare.

## Supplementary Material

CB-OLF-D5CB00124B-s001

CB-OLF-D5CB00124B-s002

## Data Availability

The data supporting this article have been included as part of the ESI.†
