# Supplementary material for: Photoactivatable and photolabile pharmacophores: lessons learned from capsaicin
Source: RSC Chem Biol. 2025 Jul 19;6(9):1473–82. doi: 10.1039/d5cb00124b (PMC12314799; doi:10.1039/d5cb00124b)
Supplement: CB-006-D5CB00124B-s001 [file CB-006-D5CB00124B-s001.pdf]

Supplementary Information (SI) for RSC Chemical Biology.
This journal is © The Royal Society of Chemistry 2025

Electronic Supporting Information

For

Photoactivatable and Photolabile Pharmacophores: Lessons Learned from Capsaicin

Nils Imse,[a,†] Lucia Rojas,[b,†] Cristina Gil Herrero,[c,d] Sebastian Thallmair,[c] JeongSeop Rhee,*[b] Nadja A. Simeth*[a,e,f]

a. Institute for Organic and Biomolecular Chemistry, Department of Chemistry, University of Göttingen, Tammannstr.2, 37077Göttingen, Germany; E-mail: nadja.simeth@uni-goettingen.de.

b. Max Planck Institute for Multidisciplinary Sciences, Hermann-Rein-Str. 3, 37075 Göttingen, Germany.

c. Frankfurt Institute for Advanced Studies, Ruth-Moufang-Str. 1, 60438 Frankfurt a. M., Germany.

d. Faculty of Biochemistry, Chemistry and Pharmacy, Goethe University Frankfurt, 60438 Frankfurt am Main, Germany.

e. Cluster of Excellence "Multiscale Bioimaging: from Molecular Machines to Networks of Excitable Cells" (MBExC), University of Göttingen, 37075 Göttingen, Germany.

f. Department of Chemistry – Ångström laboratory, Uppsala University, Box 523, 751 20 Uppsala, Sweden.

† These authors contributed equally.

Table of Contents

1. Organic Synthesis and Purification: Materials and Methods 3
   1.1. General annotation 3
   1.2. Reagents 3
   1.3. Chromatography 3
   1.4. Melting point 3
   1.5. Nuclear magnetic resonance spectroscopy (NMR) 3
   1.6. Mass spectrometry (MS) 4
   1.7. Infrared spectroscopy (IR) 4
   1.8. Liquid chromatography–mass spectrometry (LC–MS) 4
2. Organic Synthesis Procedures and Compound Characterization 5
   2.1. Fatty Acid Chains 5
   2.2. Synthesis of Parent Capsaicinoids 13
   2.3. Synthesis of Nitro-Capsaicinoids 18
3. Photophysical and Photochemical Characterization of Light-Sensitive Capsaicinoids 37
   3.1. NO_2-CAP 38
   3.2. NO_2-DHCAP 40
   3.4. ONB-CAP 44
   3.5. HPLC Trances and NMR Spectra of the Photochemical Conversion of NO_2-NV 46
   3.6. HPLC Trances of thePhotochemical Conversion of DEAC-NO_2-NV 47
4. Computational Chemistry 48
5. Biological Assays 51
6. X-Ray Analysis: Structure Tables 55
7. References 68

1. Organic Synthesis and Purification: Materials and Methods

1.1. General annotation

All reactions, involving air or moisture sensitive reagents have been handled under standard *Schlenk* technique.

1.2. Reagents

All chemicals were obtained from the chemical supplier with purities of >95%.

1.3. Chromatography

Thin-layer-chromatography (TLC) has been performed on 0.25 mm Silica-Gel 60 *F* plates with a 254 nm fluorescence indicator from *Merck*. The substance detection took place by light with the wavelength of 254 nm und 360 nm. Non-UV-active substances have been visualized by staining the plates with a potassium permanganate solution and gentle heating afterwards. For the column chromatography Merck Silica gel of the type Geduran® Si 60 (40-63 µm) was used.

1.4. Melting point

The melting point was determined with a Cole-Parmer™ Digitales Stuart™ Melting Point Apparatus SMP20.

1.5. Nuclear magnetic resonance spectroscopy (NMR)

Recordings of NMR spectra have been performed on a Bruker Avance III HD 400 at frequencies of 400 MHz (^{1}H-NMR), 101 MHz (^{13}C-NMR) and 162 MHz (^{31}P-NMR) and on a Bruker Avance III HD 300 at frequencies of 300 MHz (^{1}H-NMR) and 75 MHz (^{13}C-NMR). The chemical shift δ is shown in ppm, based on the standard tetramethyl silane. The solvent signal was used as an internal reference signal. The coupling constant *J* is shown in Hertz (Hz). For characterization the following abbreviations have been used: s (singlet), d (doublet) and m (multiplett). The solvent signals are shown in the following table S1.

Table S1: Solvent signals of Chloroform-d_1, dimethyl sulfoxide-d_6 and methanol-d_4.

**Solvent**	**^{1}H-NMR**	**^{13}C-NMR**
Chloroform-*d* ($CDCl_3$)	7.26 ppm	77.2 ppm
Dimethyl sulfoxide-d_6 (DMSO-d_6)	2.50 ppm	39.5 ppm
Methanol-d_4 (CD_3OD)	3.31 ppm	49.0 ppm

The evaluation of the NMR-spectra was done with *MestReNova* 14.3.3-33362 from *Mestrelab Research S.L.*

1.6. Mass spectrometry (MS)

ESI and ESI-HRMS spectra have been recorded on a Time-of-flight spectrometer (microTOF) from *Bruker*. Shown is the ratio between mass and charge and the relative intensities in relation to the basic peak (I = 100).

1.7. Infrared spectroscopy (IR)

The infrared spectra have been recorded on a spectrometer from *Bruker* of the type Alpha-P ATR. Liquid samples have been measured as film, solid samples were placed as pure substance. The evaluation of the spectra was done with Opus 6.5 from *Bruker*. Thr spectral range from 4000 cm^{-1} to 400 cm^{-1} was recorded.

1.8. Liquid chromatography–mass spectrometry (LC–MS)

LC-MS was performed on an Agilent 1260 Infinity II combined with an InfinityLab LC/MSD iQ mass detector (ESI) using an InfinityLab Poroshell 120 EC-C18 column at 40 °C eluted with MeCN/H_2O + 0.1% formic acid.

2. Organic Synthesis Procedures and Compound Characterization

2.1. Fatty Acid Chains

(5-Carboxypentyl)triphenylphosphonium bromide (2)

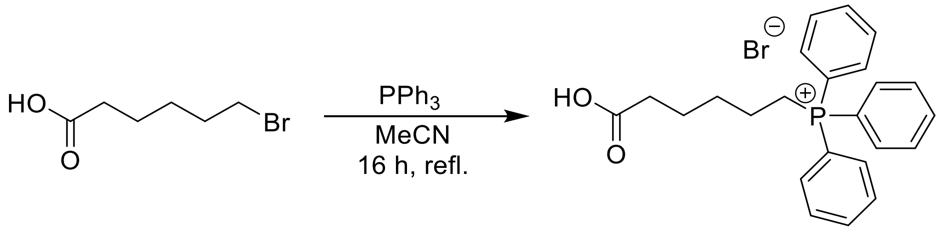

The synthesis with adapted from a literature-known procedure.[1] ω-Bromohexanoic acid (5.70 g, 29.2 mmol, 1.0 eq.) and triphenylphosphine (8.30 g, 31.4 mmol, 1.1 eq.) were added to MeCN (40 mL) and the mixture was refluxed for 16 h. Afterwards, the solvent was removed under reduced pressure, the solid residue was ground to a fine powder and washed with Et_2O (3 x 40 mL) to yield the title compound (13.30 g, 29.20 mmol, quant.) as colorless solid.

^{1}H-NMR (400 MHz, MeOD) δ = 7.98 – 7.71 (m, 15H), 3.55 – 3.40 (m, 2H), 2.38 – 2.26 (m, 2H), 1.83 – 1.58 (m, 6H).
^{13}C-NMR (101 MHz, MeOD) δ = 175.7, 134.89 (d, $^3J_{C\text{-}P}$ = 3.0 Hz), 133.44 (d, $^2J_{C\text{-}P}$ = 10.0 Hz), 130.15 (d, $^2J_{C\text{-}P}$ = 12.6 Hz), 118.56 (d, $^1J_{C\text{-}P}$ = 86.4 Hz), 33.0, 29.60 (d, $^2J_{C\text{-}P}$ = 16.4 Hz), 23.7, 21.90 (d, $^3J_{C\text{-}P}$ = 4.3 Hz), 21.25 (d, $^1J_{C\text{-}P}$ = 51.3 Hz).
^{31}P-NMR (162 MHz, MeOD) δ = 23.81.
HR-MS (ESI): m/z predicted for $C_{24}H_{26}O_2P$ $[M+H]^+$: 377.1665, found: 377.1666.
MS (ESI): m/z (%) = 377.2 (100) $[M\text{-}Br]^+$.
IR (ATR) [cm^{-1}]: 2923, 1698, 1432, 1377, 1213, 1188, 1111, 744, 717, 683.
Mp.: 196–198 °C.

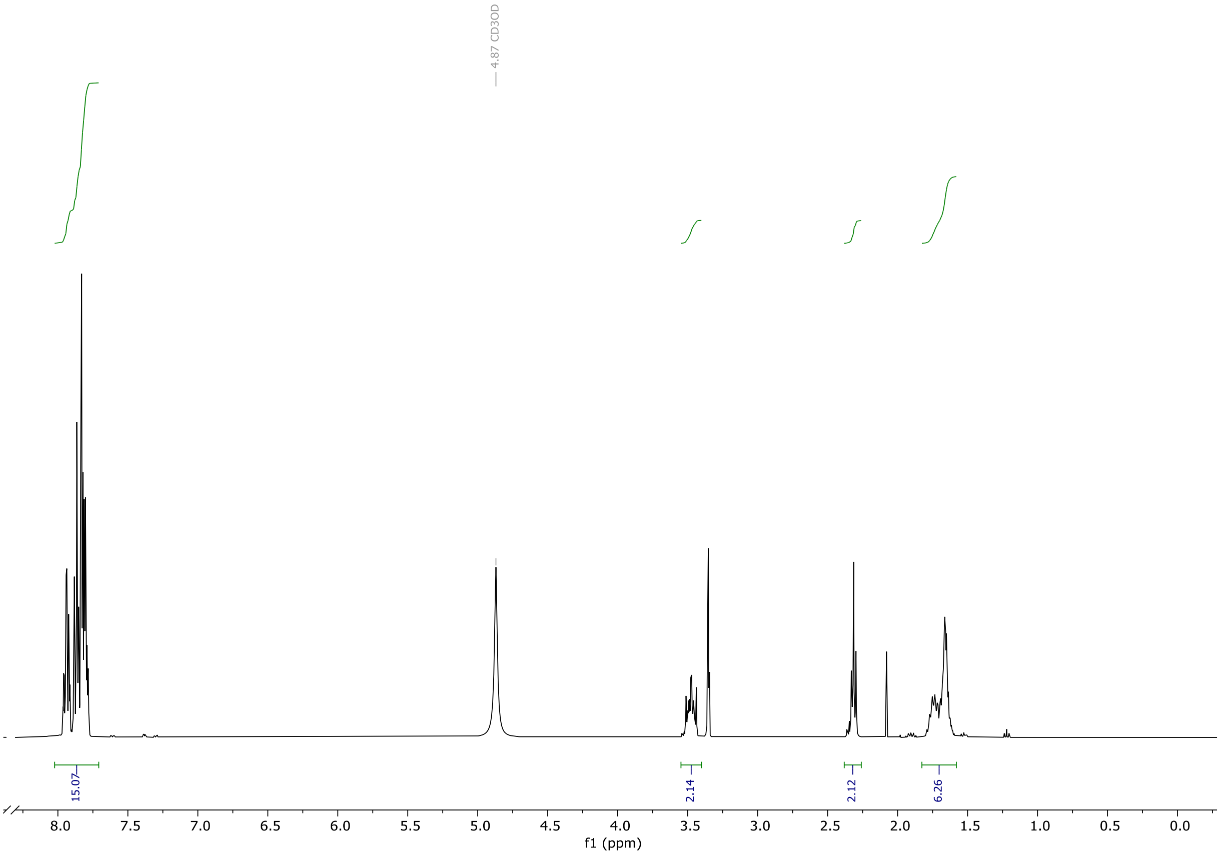

*Figure S1: ^{1}H-NMR of **2** in MeOD.*

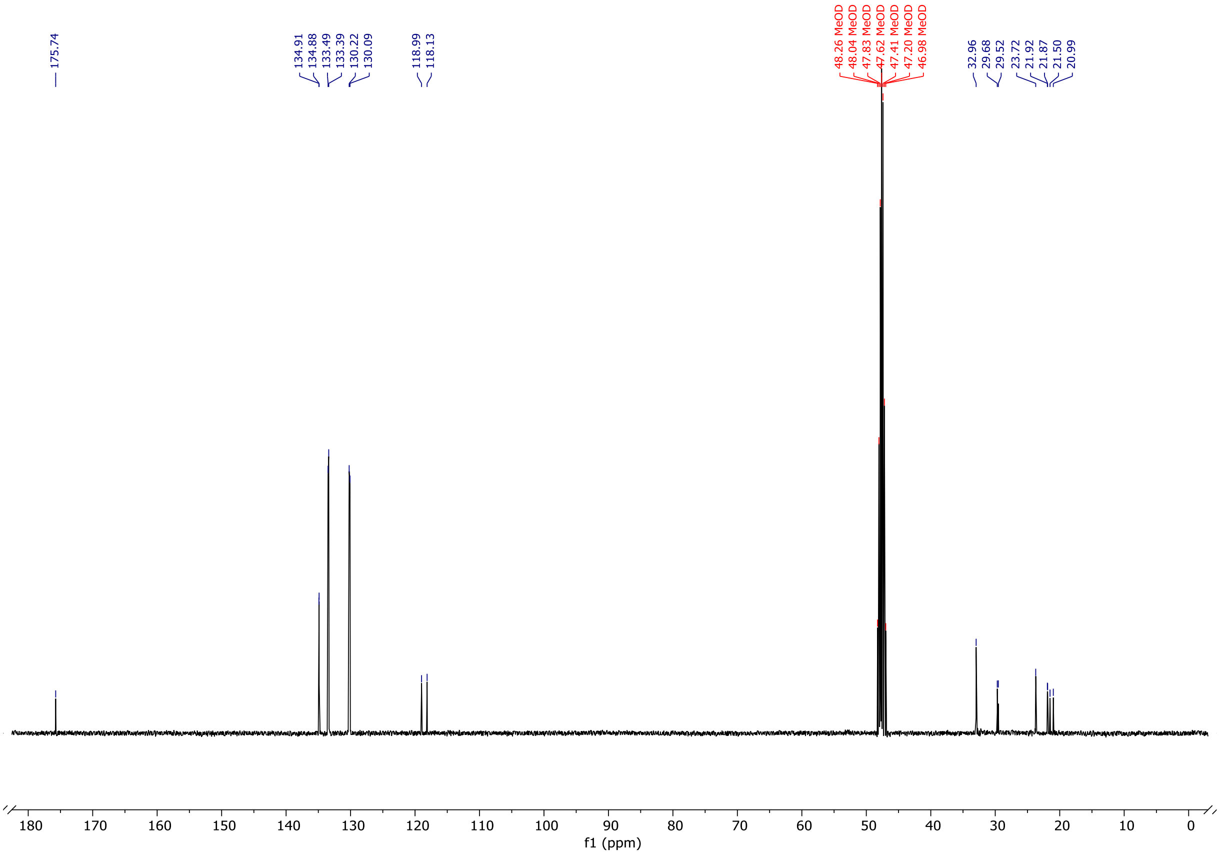

*Figure 1: ^{13}C-NMR of **2** in MeOD.*

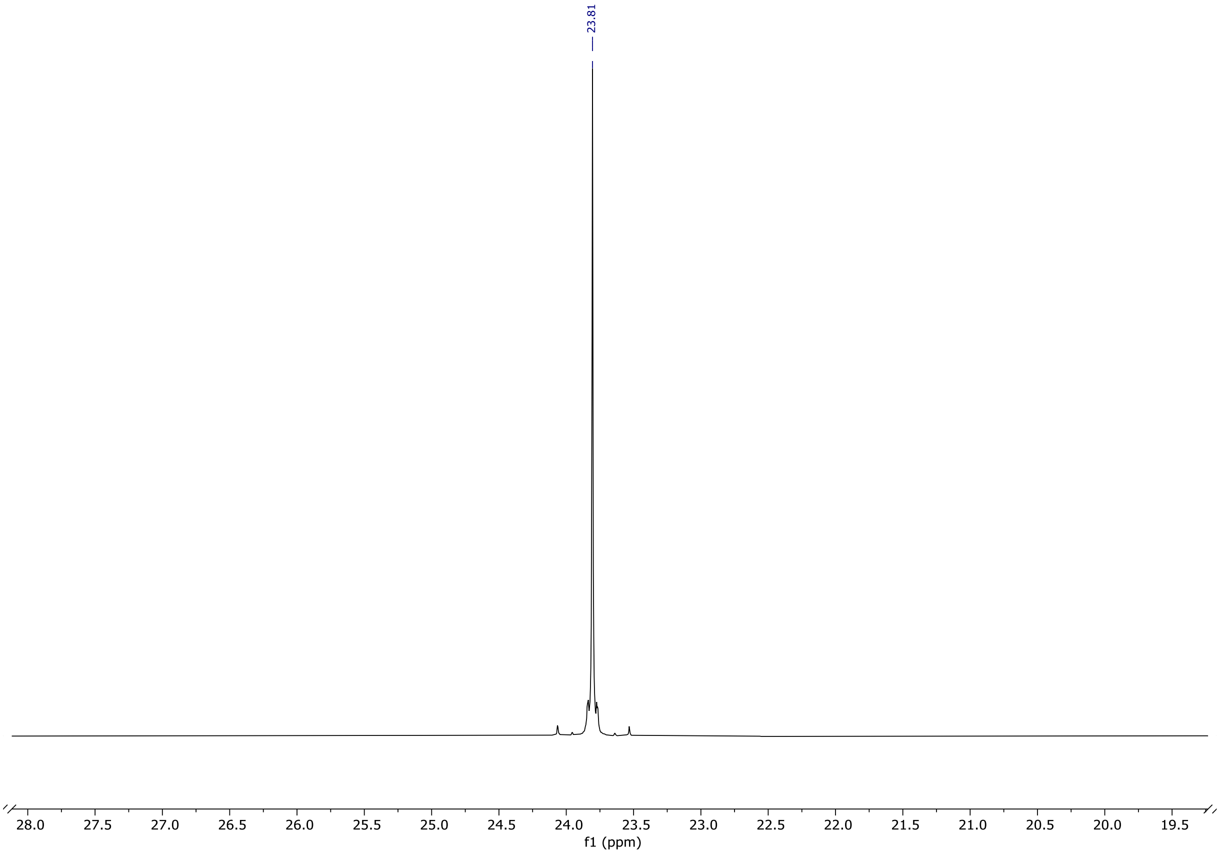

*Figure S2: ^{31}P-NMR of **2** in MeOD.*

(*E*)-8-Methylnon-6-enoic acid (5)

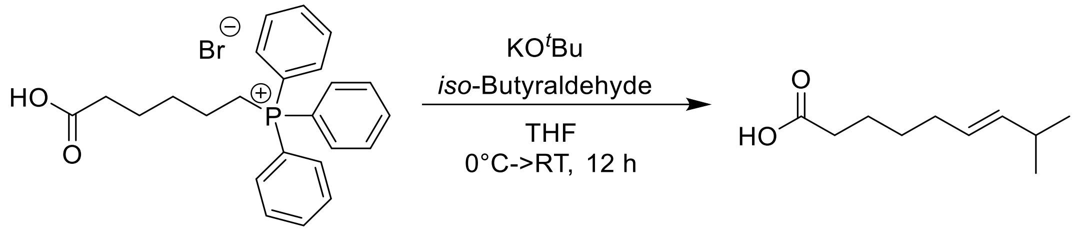

The synthesis with adapted from a literature-known procedure.[2] (5-Carboxypentyl)triphenylphosphonium bromide (3.00 g, 6.57 mmol, 1.0 eq.) was dissolved in dry THF (80 mL) and at 0°C potassium *tert*-butoxide (1.53 g, 13.89 mmol, 2.0 eq.) was added slowly, upon which the mixture turns orange. After 5 minutes, *iso*-butyraldehyde (720 µL, 7.89 mmol, 1.2 eq.) was added accompanied by a decolorization of the solution and the mixture was stirred for 16 h at RT. Afterwards, water (80 mL) was added at 0°C and the solution was acidified with 6 M HCl to a pH of 2. The Solution was extracted with EtOAc (3 x 80 mL), the combined organic layers were dried over magnesium sulfate and the solvent was removed under reduced pressure. The crude material was purified via column chromatography on silica (EtOAc: pentane 1:4 + 0.5 % HOAc) to yield the intermediate compound (660 mg) as colorless oil.

To obtain the *E*-configuration of the acid, the intermediate (660 mg) was mixed with nitric acid (6 M, 200 µL) and sodium nitrite solution (2 M, 300 µL) and the mixture was heated to 75 °C for 40 minutes. After cooling down, water (10 mL) was added, and the solution was extracted with Et_2O (3 x 20 mL). After drying of the organic layer with magnesium sulfate and removal of the solvent under reduced pressure, the crude material was purified via column chromatography on silica (EtOAc: pentane 1:4 +0.5% HOAc) to yield the title compound (484 mg, 2.8 mmol, 43%) as colorless oil. Further purification is needed, due to impurities, but the product is shown via ESI-MS and it is a mixture between the *E*-and the *Z*-conformation (4:1, via NMR and comparison with the literature).The isomeric mixture was used for further synthesis.

^{1}H-NMR (400 MHz, $CDCl_3$) δ = 5.42 – 5.28 (m, 2H), 2.37 – 2.32 (m, 2H), 2.28 – 2.17 (m, 1H), 2.10 – 1.95 (m, 2H), 1.70 – 1.58 (m, 2H), 1.45 – 1.35 (m, 2H), 1.01 – 0.91 (m, 6H).
^{13}C-NMR (101 MHz, $CDCl_3$) δ = 180.1, 138.2, 126.4, 34.0, 32.1, 31.0, 29.0, 24.1, 22.7.
HR-MS (ESI): m/z predicted for $C_{10}H_{18}O_2$[M-H]$^-$: 169.1234, found: 169.1235.
MS (ESI): m/z (%) = 169.1 (100) [M-H]$^-$.
IR (ATR) [cm^{-1}]: 2956, 2929, 2877, 1705, 1466, 1414, 1280, 1234, 971, 739.

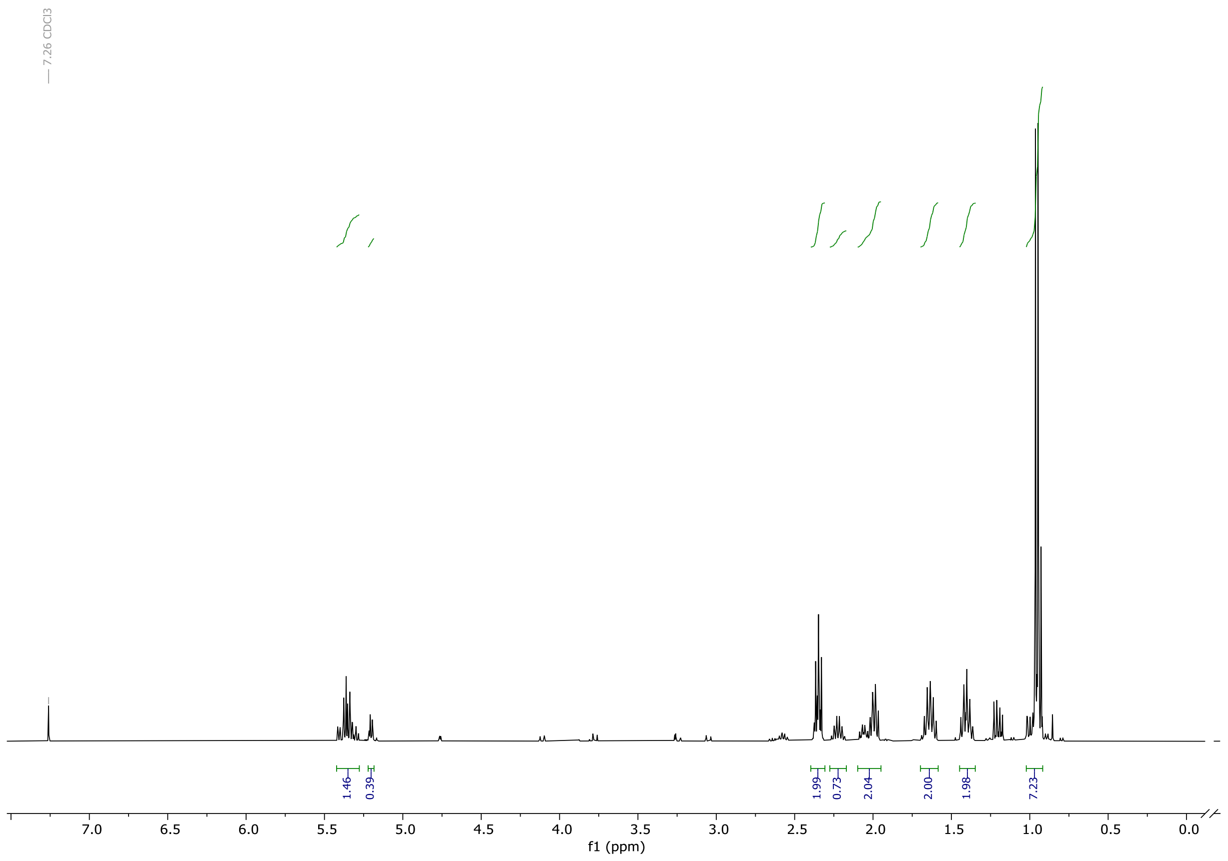

*Figure S3: ^{1}H-NMR of **5** in $CDCl_3$.*

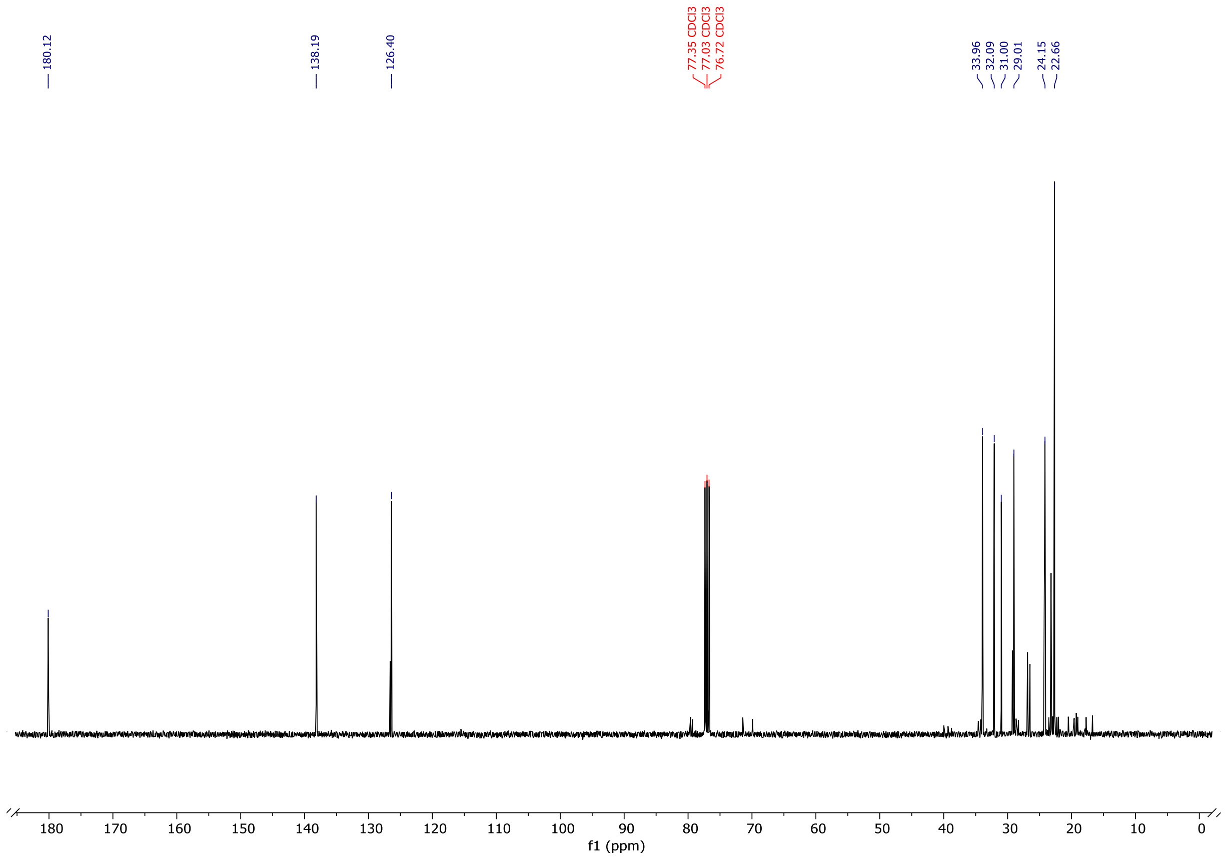

*Figure S4: ^{13}C-NMR of **5** in $CDCl_3$.*

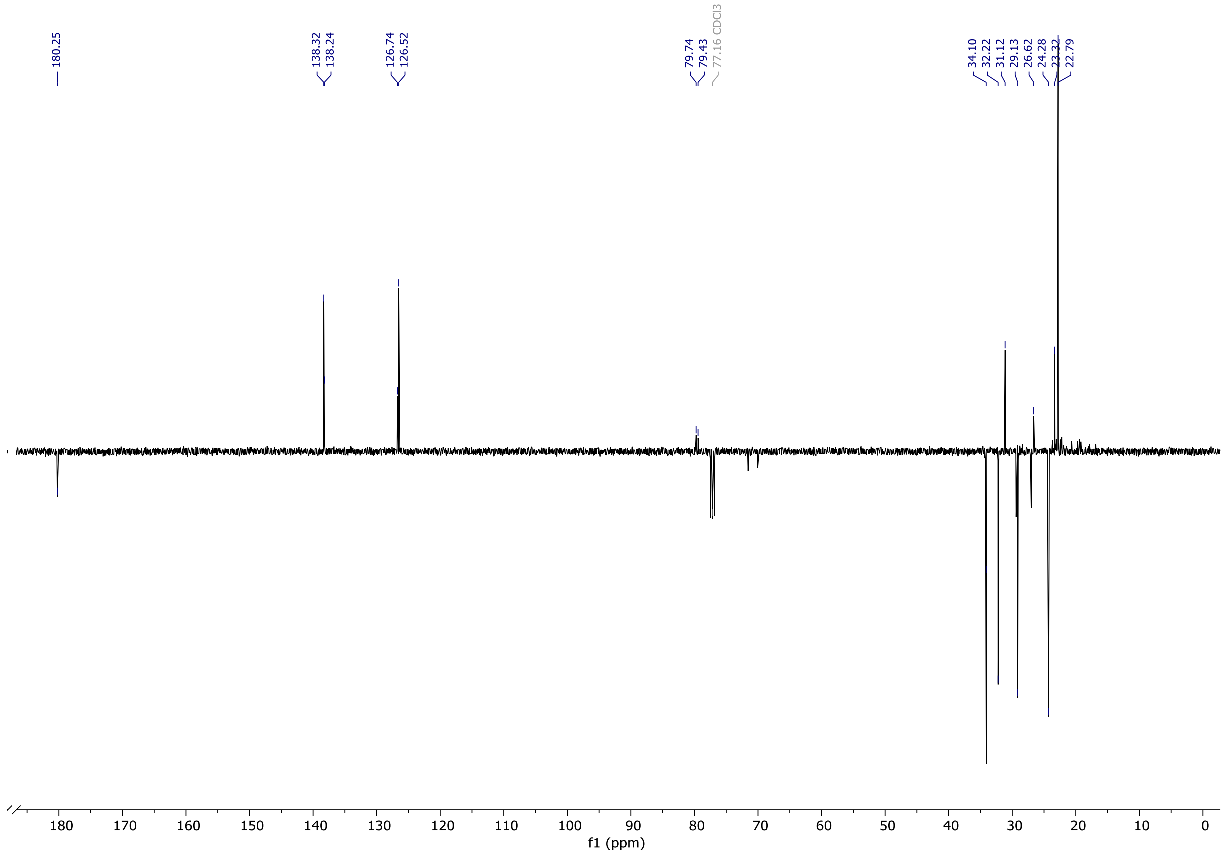

*Figure S5: ^{13}C-APT-NMR of **5** in $CDCl_3$.*

8-Methylnonanoic acid (6)

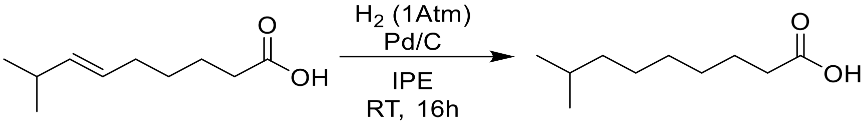

(*E*)-8-Methylnon-6-enoic acid (6.10 g, 35.80 mmol, 1.0 eq.) was dissolved in diisopropylether (100 mL) and palladium on carbon (10% Pd, 3.8 g, 10 mol%) was added. The mixture was stirred under a hydrogen atmosphere (1 atm) for 16 h and was afterwards filtered through a celite pad to remove the catalyst. The obtained product was purified via column chromatography on silica (EtOAc: pentane 1:4+0.5% HOAc) to yield the title compound (3.9 g, 22.7 mmol, 64 %) as clear oil.

^{1}H-NMR (400 MHz, $CDCl_3$) δ 2.34 (t, *J* = 7.5 Hz, 2H), 1.68 – 1.58 (m, 2H), 1.51 (dp, *J* = 13.2, 6.6 Hz, 1H), 1.39 – 1.10 (m, 11H), 1.05 – 0.87 (m, 6H), 0.86 (d, *J* = 6.6 Hz, 6H).
^{13}C-NMR (101 MHz, $CDCl_3$) δ 179.9, 39.0, 34.1, 29.5, 29.1, 28.0, 27.2, 24.7, 22.7.
HR-MS (ESI): m/z calc. for $C_{10}H_{19}O_2$ $[M-H]^-$: 171.1391, found: 171.1395.
MS (ESI): m/z (%) = 171.1 (100) $[M-H]^-$.

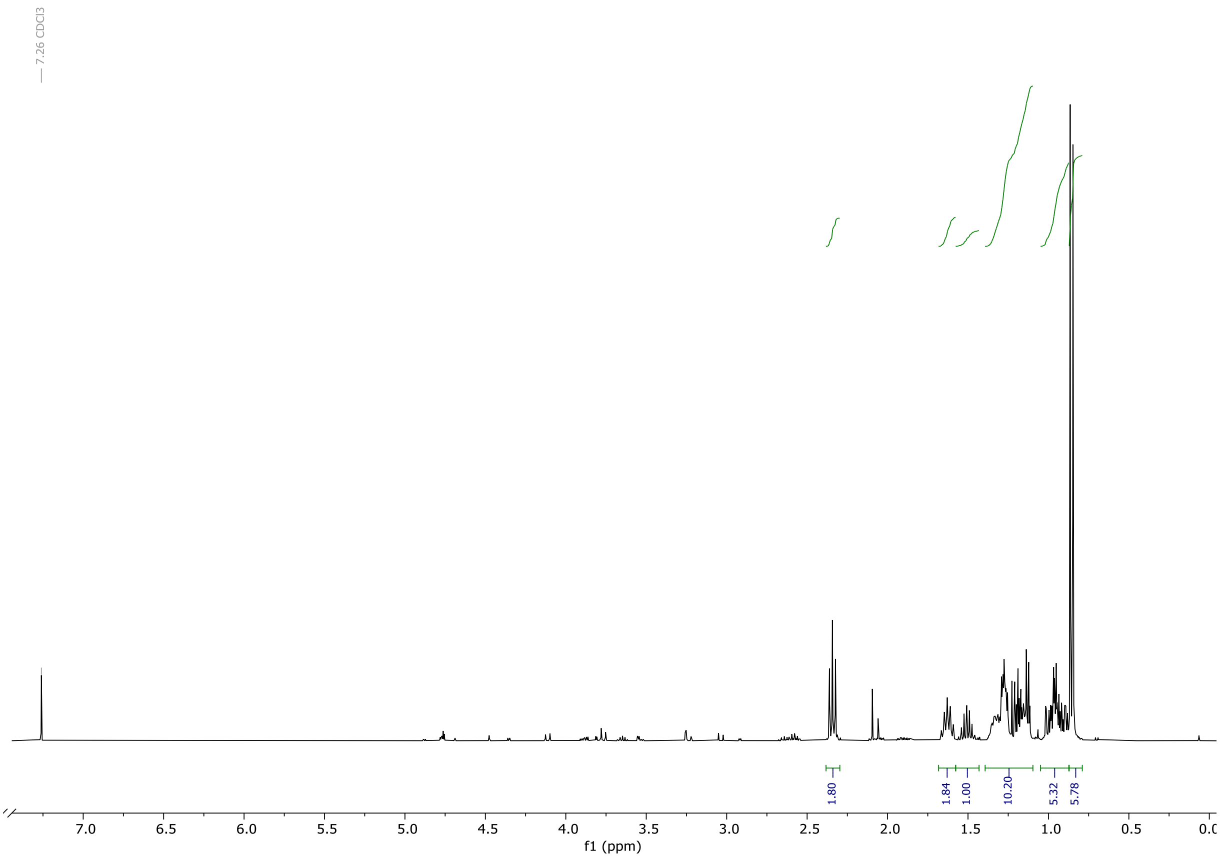

*Figure S6: ^{1}H-NMR of compound **6** in $CDCl_3$.*

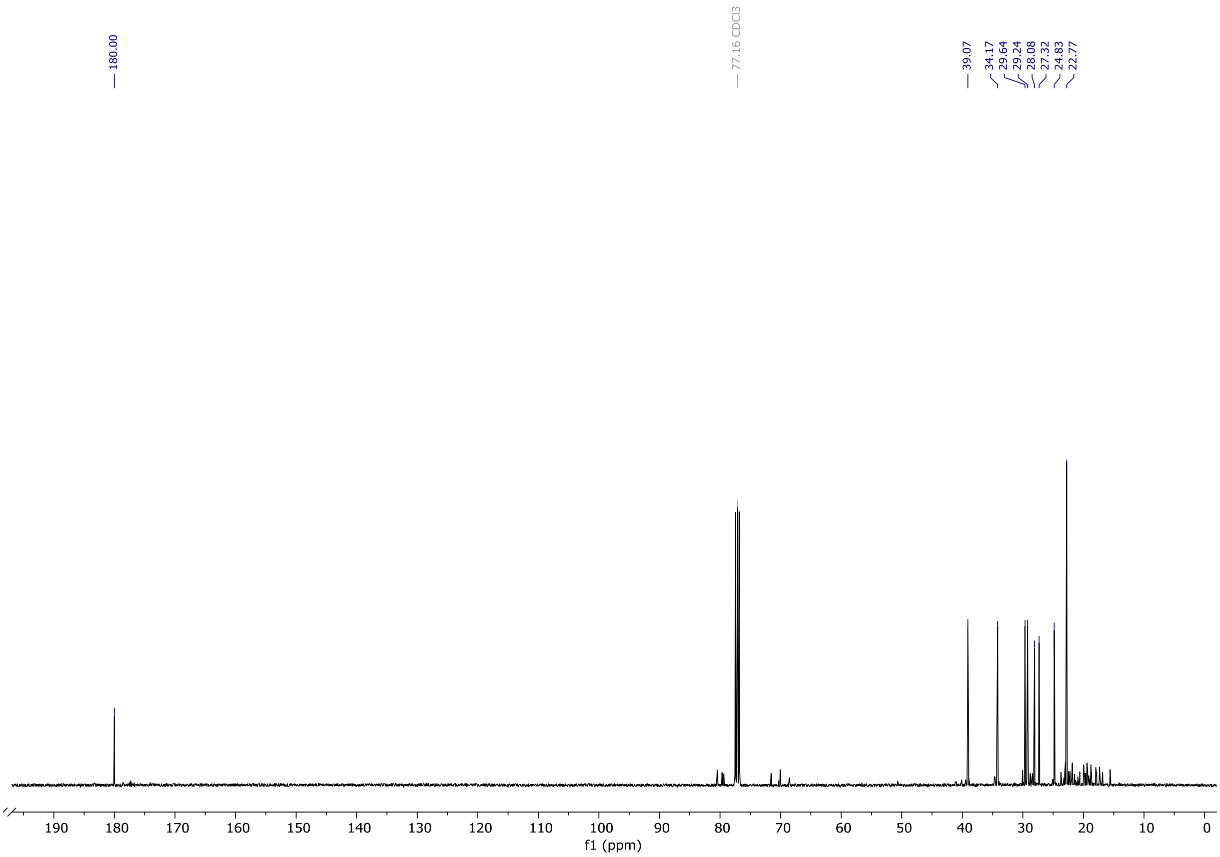

Figure S7: ^{13}C-NMR of compound **6** in $CDCl_3$.

2.2. Synthesis of Parent Capsaicinoids

Capsaicin (CAP)

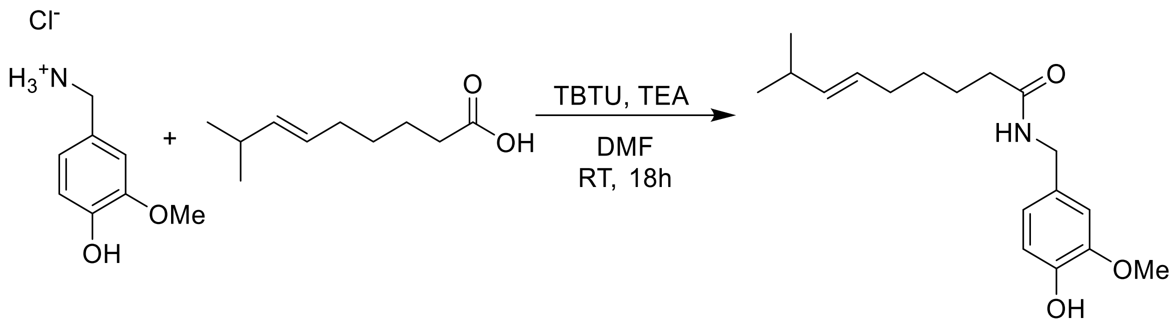

The synthesis with adapted from a literature-known procedure.[2] In dry DMF (50 mL), (*E*)-8-methylnon-6-enoic acid (1.50 g, 8.82 mmol, 1.0 eq.), vanillyl amine (2.0 g, 10.58 mmol, 1.2 eq.), TBTU (3.4 g, 10.58 mmol, 1.2 eq.) and TEA (3.7 mL, 26.44 mmol, 3.0 eq.) were dissolved at 0°C. The reaction mixture was stirred for 16 hat RT. Afterwards, the mixture was quenched with brine (50 mL) and the pH was changed to slightly acidic with aq. HCl (6 M). To the solution, water (50 mL) was added and the mixture was extracted with EtOAc (3 x 100 mL). The organic phase was dried over magnesium sulfate and the solvent was removed under reduced pressure. The crude material was further purified via column chromatography on silica (Pentane: EtOAc 1:1) to yield the title product (1.26 g, 4.13 mmol, 47%) as waxy solid.

^{1}H-NMR (400 MHz, $CDCl_3$) δ 6.86 (d, *J* = 8.0 Hz, 1H), 6.81 – 6.71 (m, 2H), 6.13 (t, *J* = 5.7 Hz, 1H), 5.28 – 5.12 (m, 2H), 4.34 (d, *J* = 5.6 Hz, 2H), 3.86 (s, 3H), 2.66 – 2.47 (m, 1H), 2.23 (t, *J* = 7.6 Hz, 2H), 2.10 – 2.02 (m, 3H), 1.77 – 1.59 (m, 2H), 1.39 (tt, *J* = 10.2, 6.4 Hz, 2H), 0.95 (d, *J* = 6.7 Hz, 6H).

^{13}C-NMR (101 MHz, $CDCl_3$) δ 173.3, 146.9, 145.2, 138.0, 130.2, 126.7, 120.7, 114.5, 110.8, 55.9, 43.6, 36.7, 29.6, 27.0, 26.5, 25.5, 23.2.
HR-MS (ESI): m/z calc. for $C_{18}H_{27}NO_3Na$ $[M+Na]^+$: 328.1883, found: 328.1885.
MS (ESI): m/z (%) = 328.2 (100) $[M+Na]^+$.

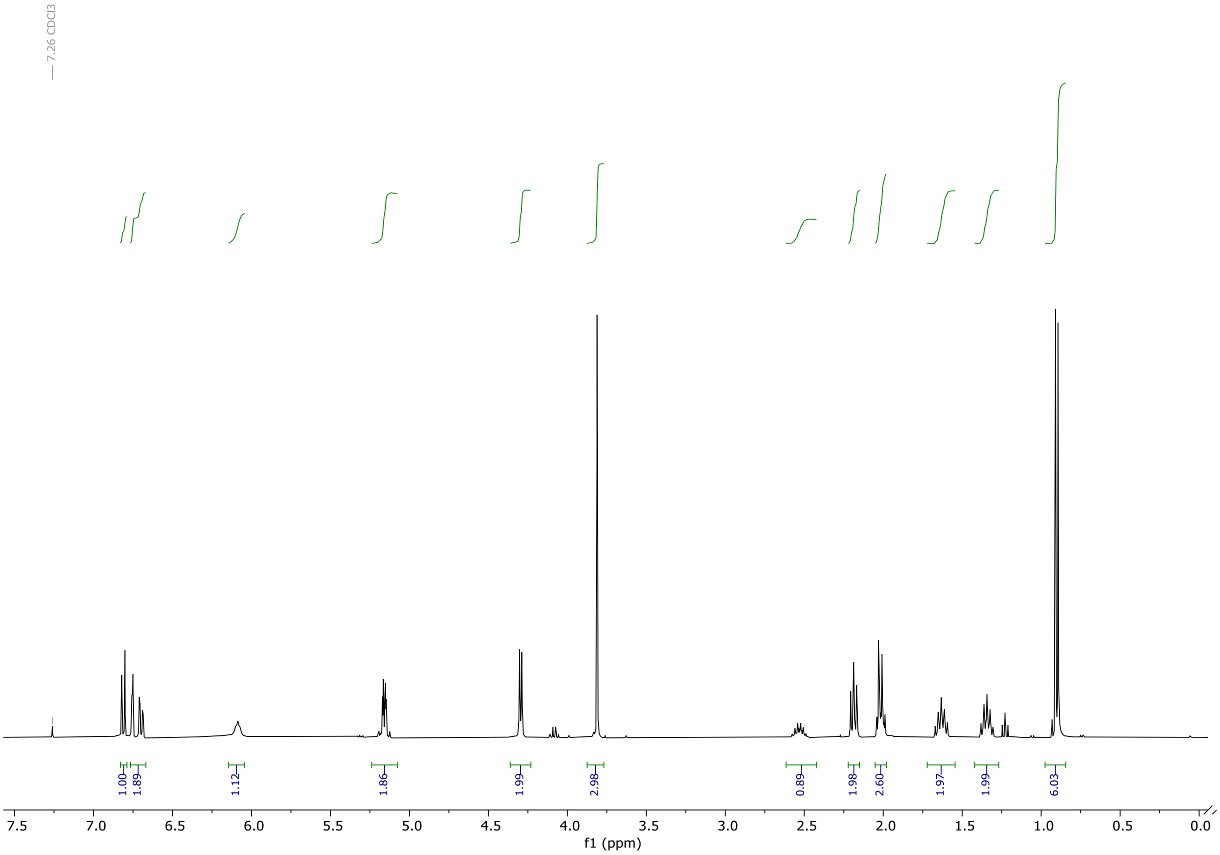

*Figure S8: ^{1}H-NMR of compound **CAP** in $CDCl_3$.*

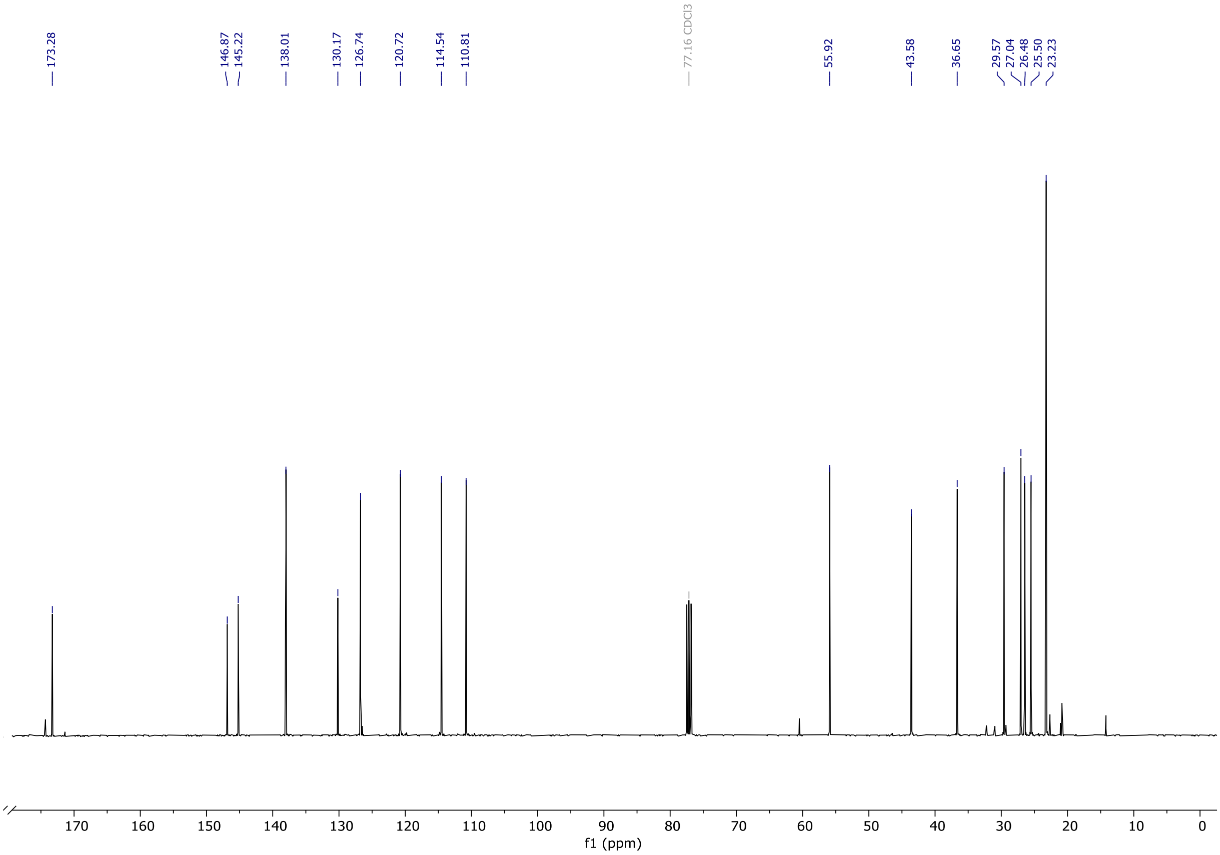

*Figure S9: ^{13}C-NMR of compound **CAP** in $CDCl_3$.*

Dihydrocapsaicin (DHCAP)

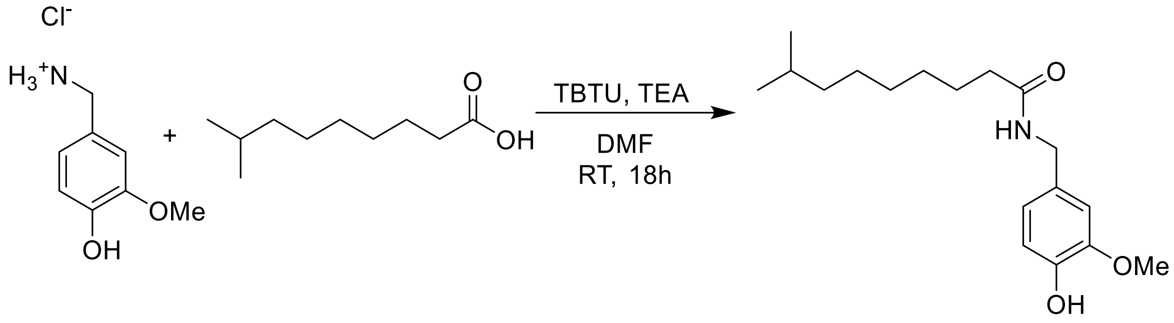

The synthesis with adapted from a literature-known procedure.[2] In dry DMF (50 mL), 8-methylnonanoic acid (1.52 g, 8.82 mmol, 1.0 eq.), vanillyl amine (2.00 g, 10.58 mmol, 1.2 eq.), TBTU (3.4 g, 10.58 mmol, 1.2 eq.) and TEA (3.7 mL, 26.44 mmol, 3.0 eq.) were dissolved at 0°C. The reaction mixture was stirred for 16 h at RT. Afterwards, the mixture was quenched with brine (50 mL) and the pH was changed to slightly acidic with aq. HCl (6 M). To the solution, water (50 mL) was added and extracted with EtOAc (3 x 100 mL). The organic phase was dried over magnesium sulfate and the solvent was removed under reduced pressure. The crude material was further purified via column chromatography on silica (Pentane: EtOAc 1:1) to yield the title product (1.20 g, 3.91 mmol, 44 %) as clear oil.

^{1}H-NMR (400 MHz, $CDCl_3$) δ 6.85 (d, J = 8.0 Hz, 1H), 6.81 – 6.72 (m, 2H), 5.80 (s, 1H), 4.35 (d, J = 5.5 Hz, 2H), 3.87 (s, 3H), 2.24 – 2.16 (m, 2H), 2.08 (s, 1H), 1.69 – 1.59 (m, 2H), 1.56 – 1.42 (m, 1H), 1.36 – 1.21 (m, 6H), 1.18 – 1.08 (m, 2H), 0.85 (d, J = 6.6 Hz, 6H).
^{13}C-NMR (101 MHz, $CDCl_3$) δ 173.3, 146.7, 145.2, 130.2, 120.8, 114.4, 110.7, 56.0, 43.7, 39.0, 36.8, 29.6, 29.4, 28.0, 27.3, 25.9, 22.7, 20.7.
HR-MS (ESI): m/z calc. for $C_{18}H_{29}NO_3Na$ $[M+Na]^+$: 330.2047, found: 330.2040.
MS (ESI): m/z (%) = 330.2 (100) $[M+Na]^+$.

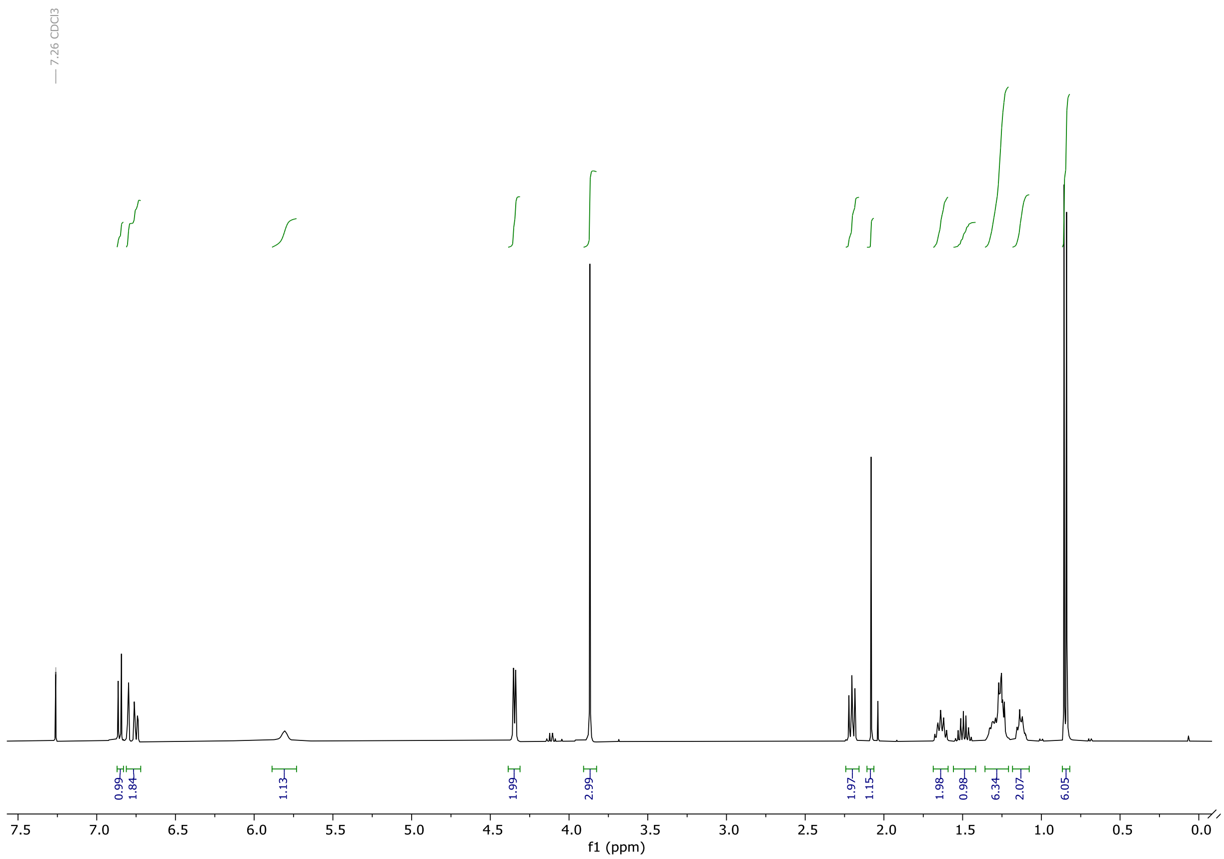

*Figure S10: ^{1}H-NMR of compound **DHCAP** in $CDCl_3$.*

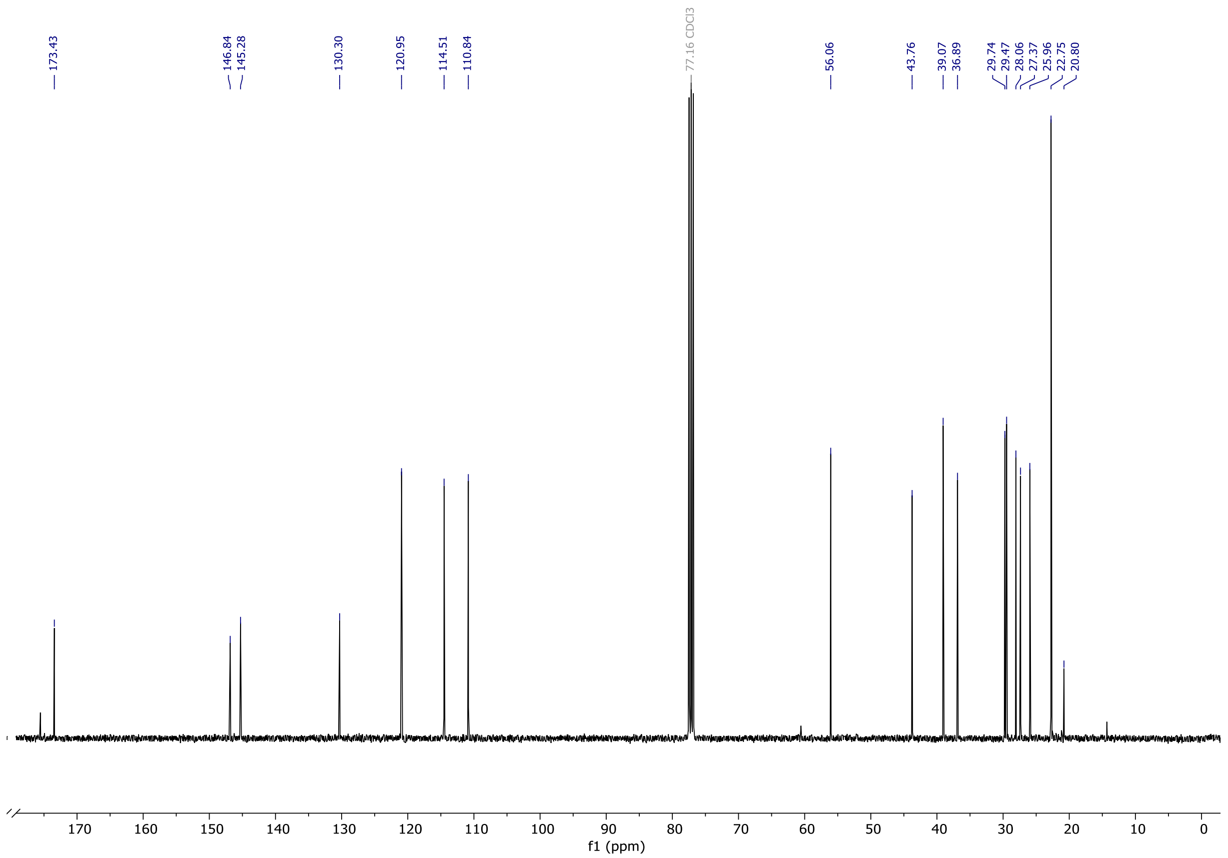

*Figure S11: ^{13}C-NMR of compound **DHCAP** in $CDCl_3$.*

N-(4-hydroxy-3-methoxybenzyl)nonanamide (NV)

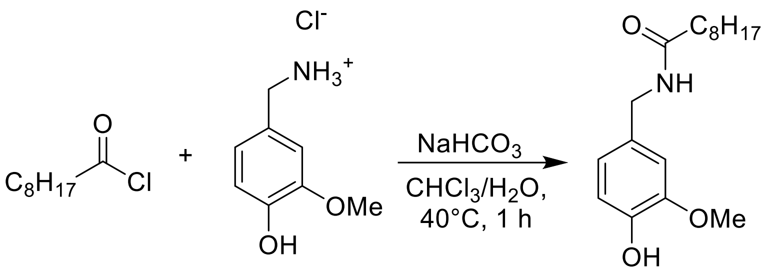

The synthesis was adapted from a known procedure.[3] Vanillyl amine hydrochloride (2.00 g, 10.50 mmol, 1.0 eq.) was dissolved with sodium bicarbonate (3.78 g, 45 mmol, 4.3 eq.) in water (30 mL) and stirred for 10 minutes. Then, chloroform (40 mL) was added, and the mixture was stirred for another 15 minutes. Pelargonyl chloride (2.50 mL, 2.45 g, 13.9 mmol, 1.3 eq.), dissolved in chloroform (10 mL) was added slowly over 30 minutes at RT. The reaction mixture was heated to 40°C for 1 h and afterwards extracted with chloroform (3 x 20 mL). The organic phase was washed with 1 M HCl (2 x 50 mL) and then the solvent was removed under reduced pressure. The crude product was purified via column chromatography on silica (pentane: EtOAc 3:1 → 1:1 → 1:1 + 10% MeOH). The title compound was yielded as yellowish oily substance (2.80, 9.57 mmol, 91%).

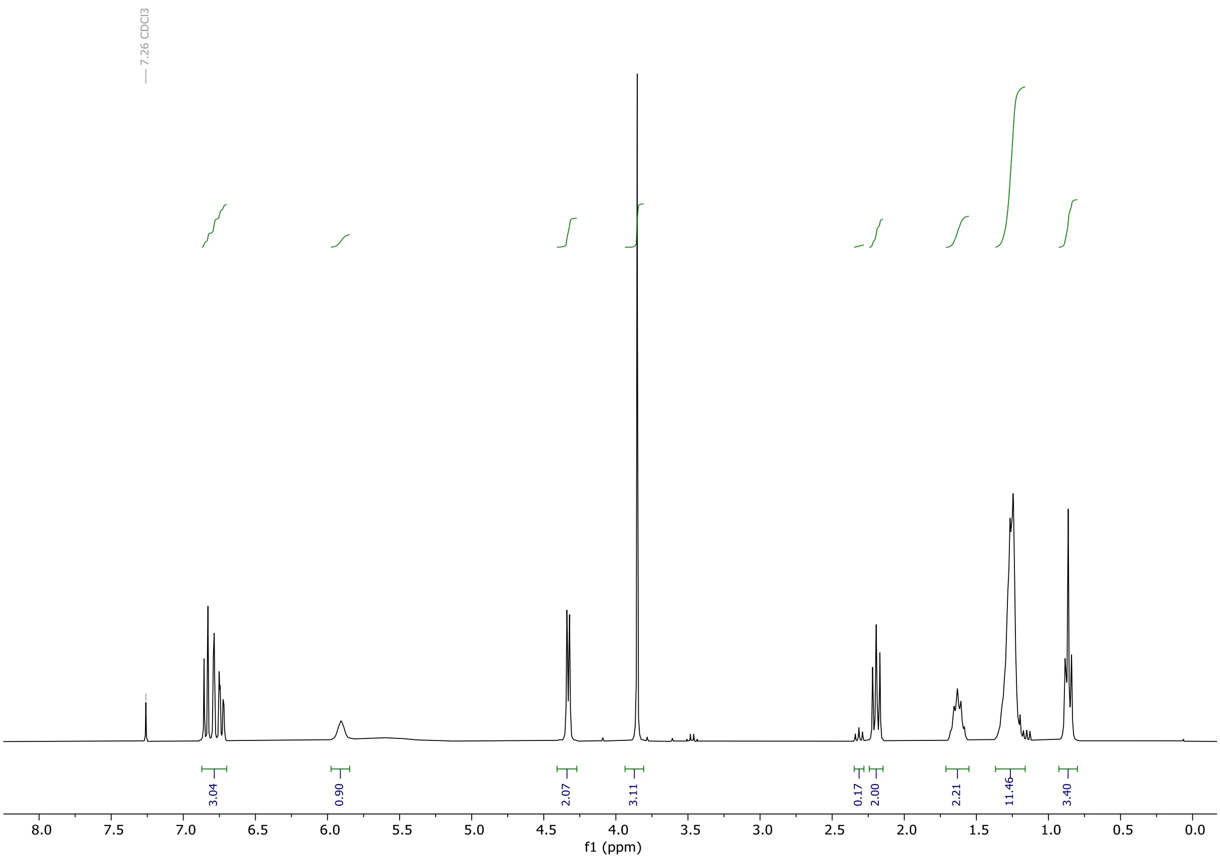

*Figure S12: ^{1}H-NMR of compound **NV** in $CDCl_3$.*

2.3. Synthesis of Nitro-Capsaicinoids

4-(Hydroxymethyl)-2-methoxy-5-nitrophenol (13)

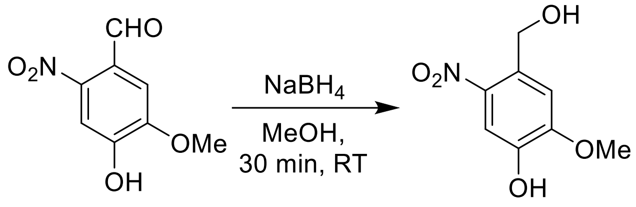

12 **13**

Compound **12**[4] (6.27 g, 31.86 mmol, 1.0 eq.) was dissolved in MeOH (250 mL) and sodium borohydride (2.90 g, mmol, 2.0 eq.) was added slowly. The reaction mixture was stirred for 30 minutes and afterwards, HCl (6 M) was added until a pH of 7 was reached. The solvent was mostly removed, and the suspension was filtered off and washed with water. The filter cake was dried *in vacuo*, to yield the title compound (5.10 g, 25.6 mmol, 80%) as yellowish orange solid.

^{1}**H-NMR** (300 MHz, DMSO): δ = 9.92 (s, 1H), 7.76 (s, 1H), 7.34 (s, 1H), 4.79 (d, *J* = 5.3 Hz, 2H), 3.90 (s, 3H).
^{13}**C-NMR** (75 MHz, DMSO): δ = 153.3, 145.4, 138.9, 132.9, 111.7, 110.5, 60.6, 56.4.
HR-MS (ESI): m/z calc. for C_8H_9NO5 $[M-H]^-$: 198.0408, found: 198.0415.
MS (ESI): m/z (%) = 198.0 (100) $[M-H]^-$, 397.1 (70) $[2M-H]^-$.
IR (ATR) [cm^{-1}]: 2360, 2157, 2005, 1583, 1525, 1494, 1289, 1213, 1151, 1066, 1035.
Mp.: 186–189 °C.

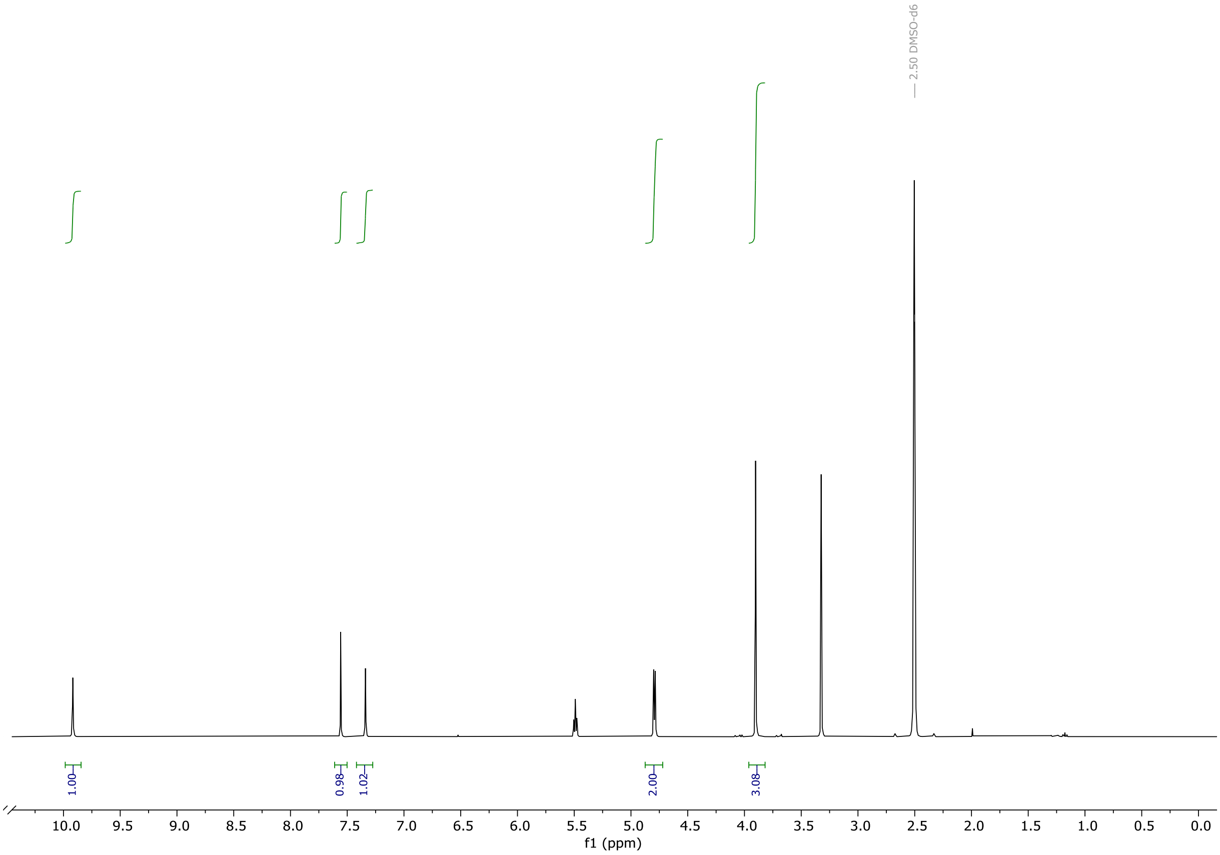

*Figure S13: ^{1}H-NMR of compound **13** in $CDCl_3$.*

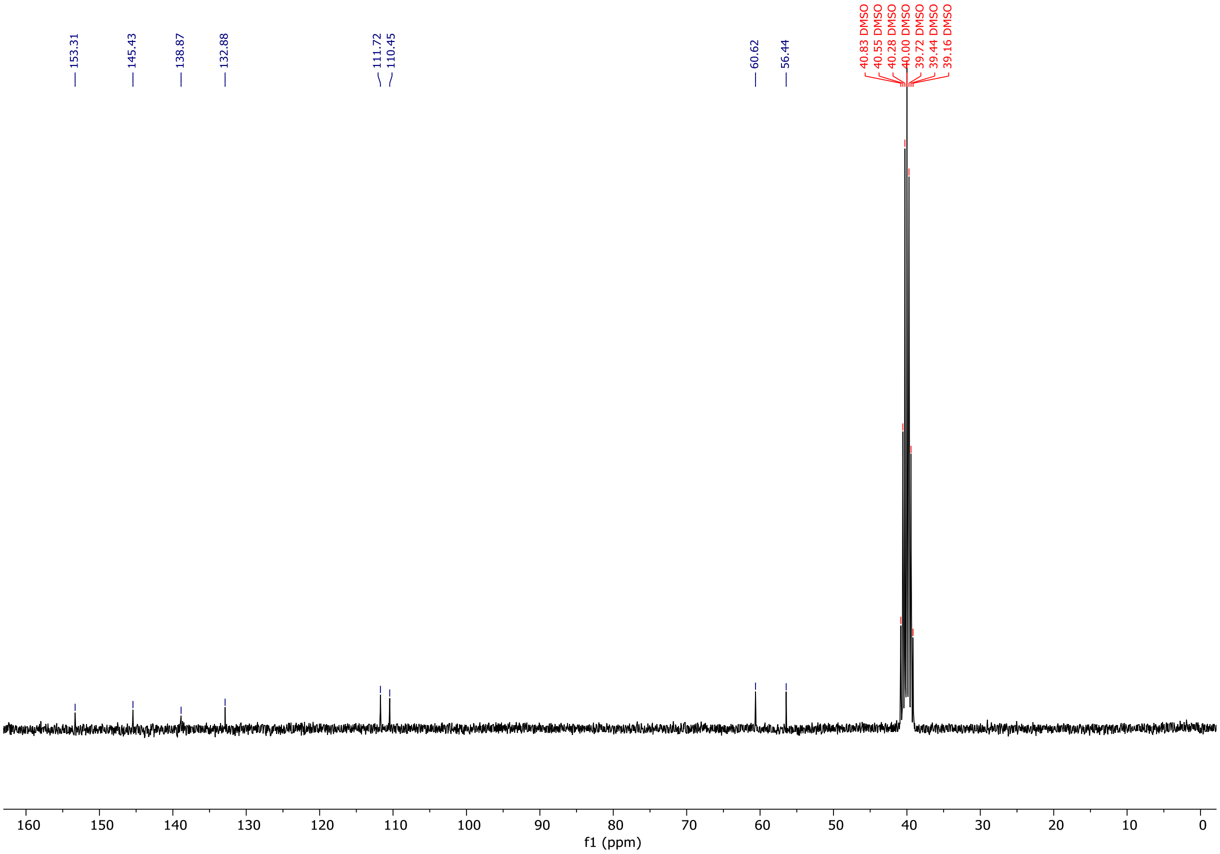

*Figure S14: ^{13}C-NMR of compound **13** in $CDCl_3$.*

4-(Bromomethyl)-2-methoxy-5-nitrophenol (14)

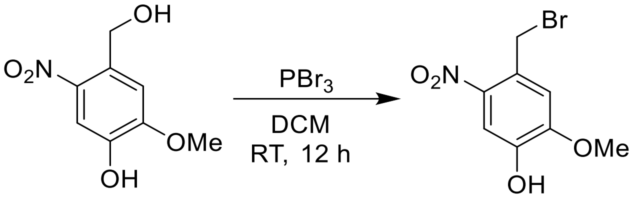

13 **14**

Compound **14** (2.50 g, 12.56 mmol, 1.0 eq.) was dissolved in DCM (80 mL) and the reaction mixture was chilled with an ice bath. Afterwards, PBr_3 (4.0 mL, 42 mmol, 3.3 eq.) was added slowly and the mixture was stirred for 2 d while reaching room temperature. Water (100 mL) was added, the organic phase was separated, and the aqueous phase was extracted with EtOAc (3 x 80 mL). The combined organic phases were dried with magnesium sulfate and the solvent was removed *in vacuo* to yield the crude product, which was purified by column chromatography (20% EtOAc/Pentane) on silica to yield the title compound (3.22 g, 12.29 mmol, 98%) as yellowish solid.

^{1}**H-NMR** (300 MHz, DMSO): δ = 7.56 (s, 1H), 7.30 (s, 1H), 4.94 (s, 2H), 3.90 (s, 3H).
^{13}**C-NMR** (75 MHz, DMSO): δ = 152.6, 147.5, 140.4, 125.9, 115.4, 112.6, 56.8, 31.9.
HR-MS (ESI): m/z calc. for $C_8H_8NO_4$ $[M+H]^+$: 182.0448, found: 182.0458.
MS (ESI): m/z (%) = 182.1 (100) [M-Br]+.
IR (ATR) [cm^{-1}]: 3485, 2360, 2339, 1518, 1497, 1332, 1283, 1215, 1056, 897, 806..
Mp.: 118–121 °C.

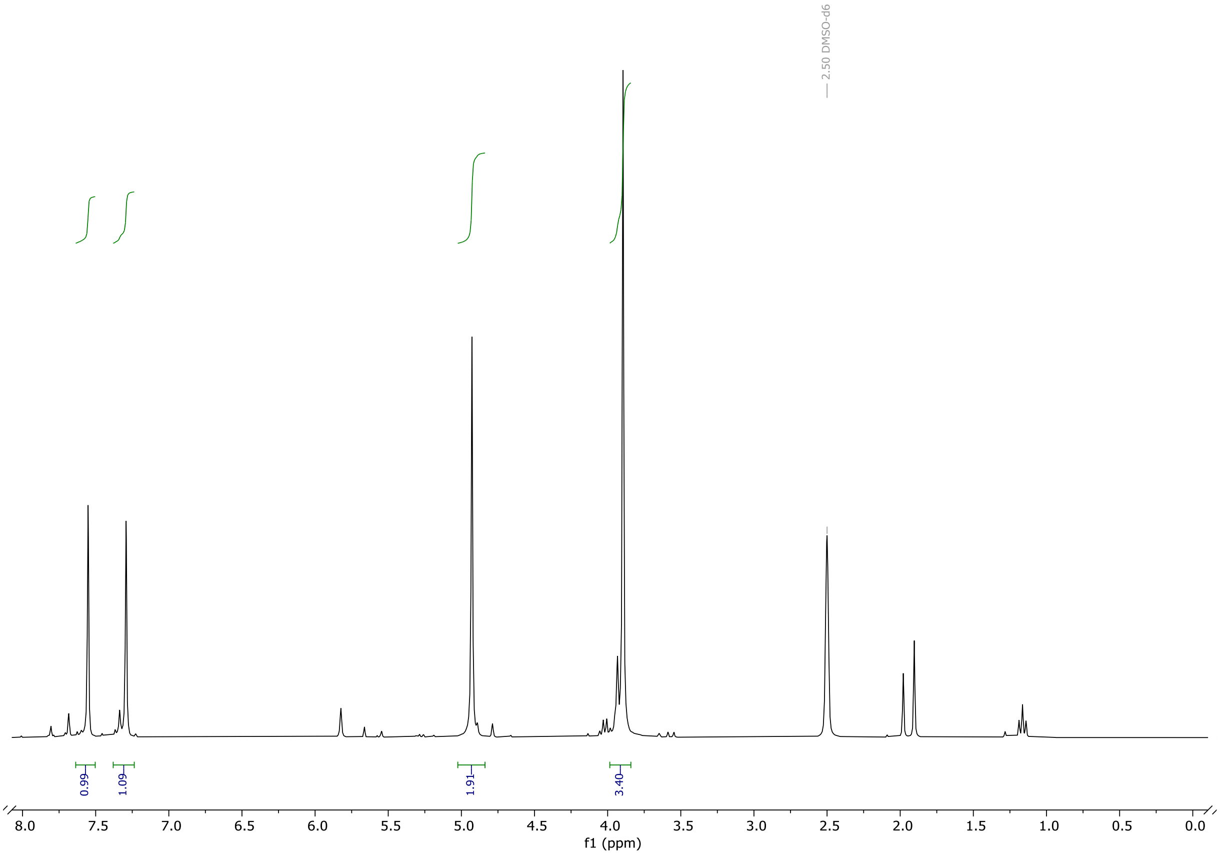

*Figure S15: ^{1}H-NMR of compound **14** in $CDCl_3$.*

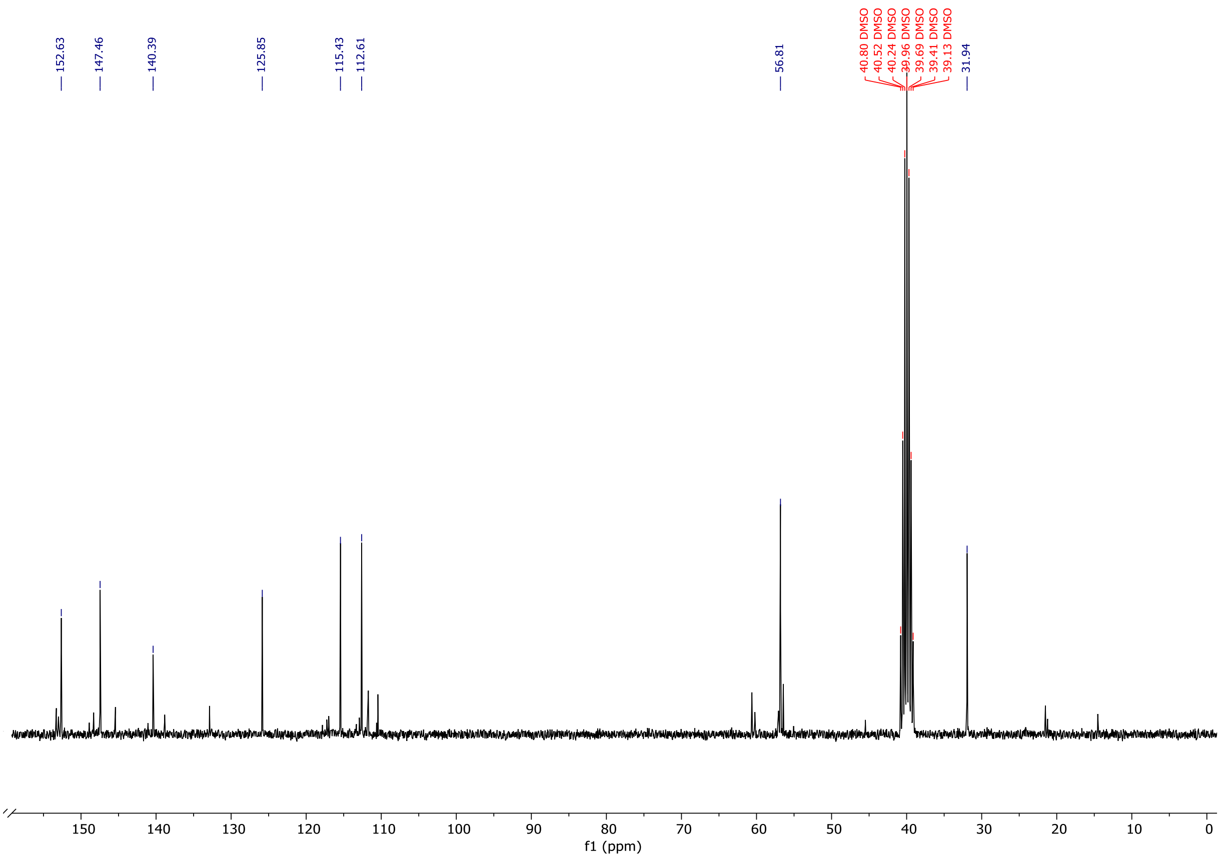

*Figure S16: ^{13}C-NMR of compound **14** in $CDCl_3$.*

4-(Aminomethyl)-2-methoxy-5-nitrophenol (15)

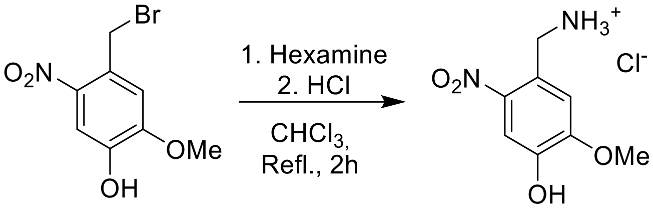

14 **15**

Hexamine (1.81 g, 12.9 mmol, 1.1 eq.) and **14** (3.22 g, 12.3 mmol, 1.0 eq.) were dissolved in chloroform (80 mL) and the mixture was refluxed for 2 h. After the mixture cooled down, the white precipitate was filtered off and washed with chloroform. The intermediate was dried and refluxed in a mixture of ethanol (25 mL), water (3.4 mL), and conc. hydrochloric acid (7.20 mL) until the solution became clear. The solution was chilled to 0 °C and the precipitated ammonium chloride was filtered off. The solvent of the so-obtained solution was removed *in vacuo* to give the title compound (2.66 g, 11.40 mmol, 92%) as hydrochloride salt.

^{1}**H-NMR** (300 MHz, MeOD): δ = 7.69 (s, 1H), 7.27 (s, 1H), 4.39 (s, 2H), 4.03 (s, 3H).
^{13}**C-NMR** (75 MHz, MeOD): δ = 154.0, 149.2, 142.8, 122.0, 116.3, 113.5, 57.4, 42.4.
HR-MS (ESI): m/z calc. for $C_8H_{10}N_2O_4$ [M-H]$^+$: 199.0713, found: 199.0726.
MS (ESI): m/z (%) = 182.0 (100) [M-NH_3]$^+$, 199.1 (50) [M+H]$^+$.
IR (ATR) [cm^{-1}]: 2360, 2339, 1518, 1457, 1332, 1286, 1207, 1056, 999, 882, 842, 806.
Mp.: decomposition at ~ 185 °C.

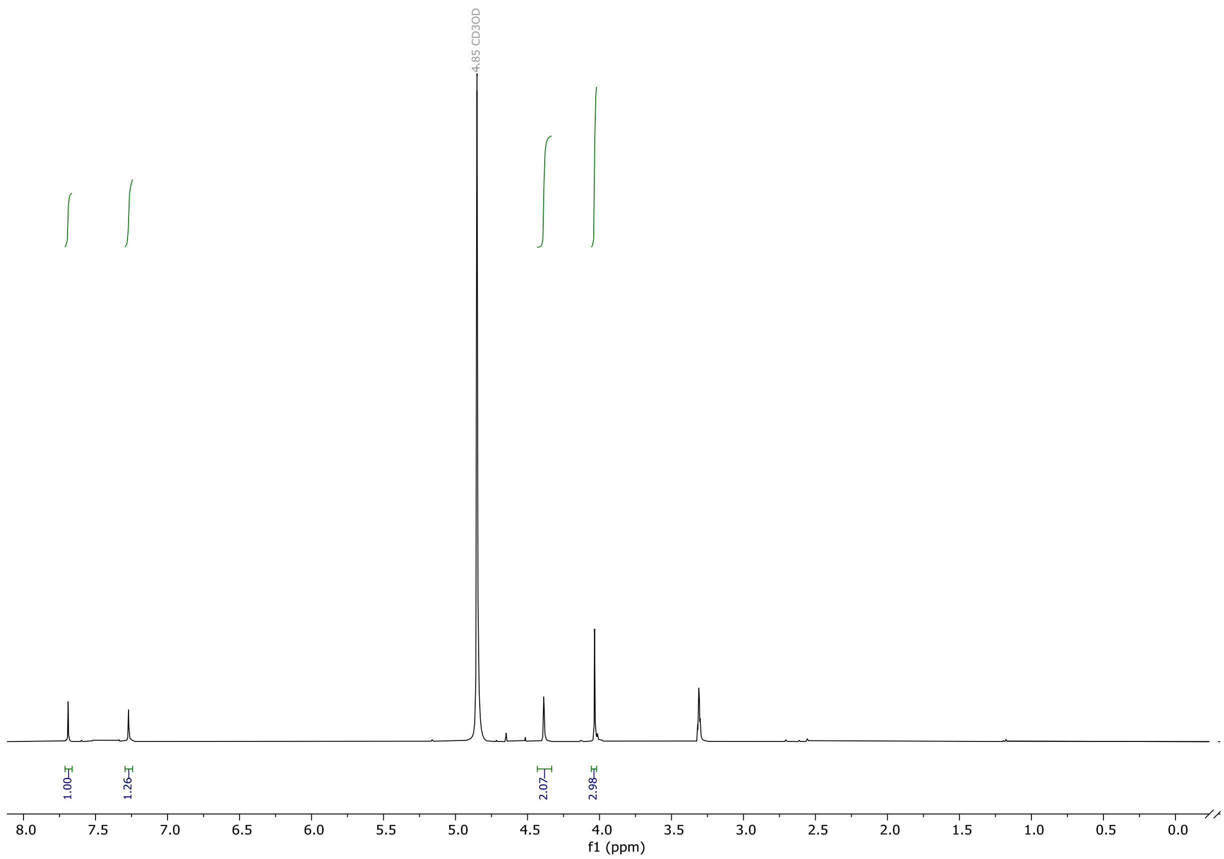

*Figure S17: ^{1}H-NMR of compound **15** in $CDCl_3$.*

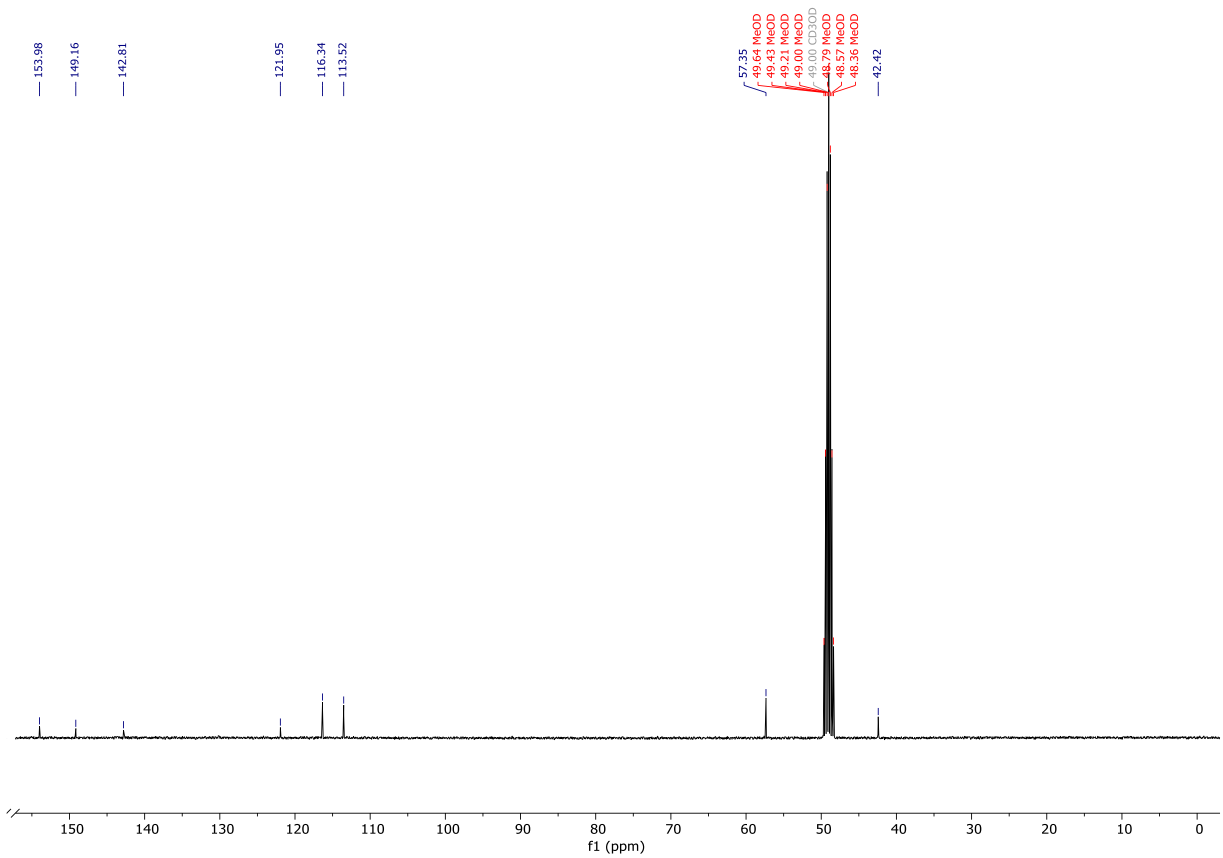

*Figure S18: ^{13}C-NMR of compound **15** in $CDCl_3$.*

(*E*)-N-(4-hydroxy-5-methoxy-2-nitrobenzyl)-8-methylnon-6-enamide (NO_2-CAP)

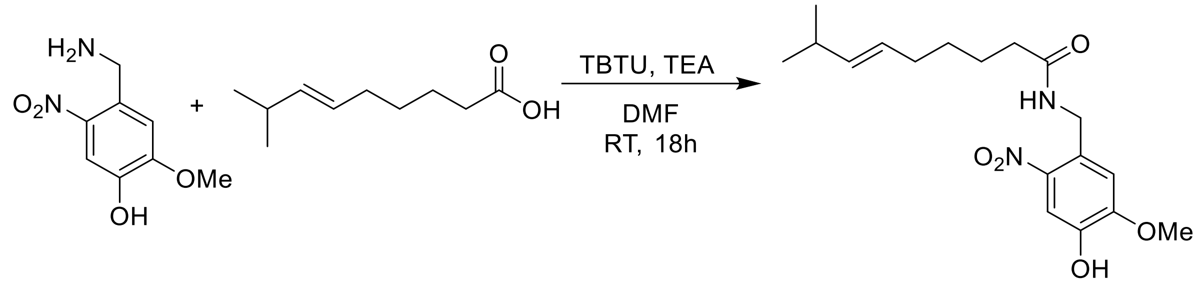

In dry DMF (40 mL), (*E*)-8-methylnon-6-enoic acid (1.13 g, 6.7 mmol, 1.0 eq.), 4-(aminomethyl)-2-methoxy-5-nitrophenol (1.87 g, 8.0 mmol, 1.2 eq.), TBTU (2.57 g, 8.0 mmol, 1.2 eq.) and TEA (2.8 mL, 20.0 mmol, 3.0 eq.) were dissolved at 0°C. The reaction mixture was stirred for 16 h. Afterwards, the mixture was quenched with brine (50 mL) and the pH was changed to slightly acidic with aq. HCl (6 M). To the solution, water (50 mL) was added and extracted with EtOAc (3 x 100 mL). The organic phase was dried over magnesium sulfate and the solvent was removed under reduced pressure. The crude material was further purified via column chromatography on silica (pentane: EtOAc 1:1) and recrystallisation (2:1 hexane: Et_2O) to yield the title compound (490 mg, 1.4 mmol, 22%) as shiny yellow crystals.

^{1}H-NMR (400 MHz, $CDCl_3$) δ 7.69 (s, 1H), 7.07 (s, 1H), 6.54 (t, *J* = 6.5 Hz, 1H), 5.23 – 5.06 (m, 2H), 4.59 (d, *J* = 6.5 Hz, 2H), 3.94 (s, 3H), 2.53 (ddt, *J* = 13.2, 8.7, 6.6 Hz, 1H), 2.29 – 2.14 (m, 2H), 2.01 (dddd, *J* = 7.4, 5.7, 4.4, 1.3 Hz, 2H), 1.70 – 1.54 (m, 2H), 1.42 – 1.25 (m, 2H), 0.90 (d, *J* = 6.6 Hz, 6H).

^{13}C-NMR (101 MHz, $CDCl_3$) δ 173.5, 151.3, 145.3, 141.2, 138.0, 127.6, 126.6, 114.1, 111.8, 56.6, 41.7, 36.6, 29.5, 27.0, 26.5, 25.3, 23.2.

HR-MS (ESI): m/z calc. for $C_{18}H_{26}N_2O_5Na$ $[M+Na]^+$: 373.1734 found: 373.1746.

MS (ESI): m/z (%) = 373.2 (100) $[M+Na]^+$.

IR (ATR) [cm^{-1}]: 1644, 1591, 1525, 1460, 1322, 1280, 1237, 1207, 1057, 879.

Mp.: 92.8 – 93.2°C.

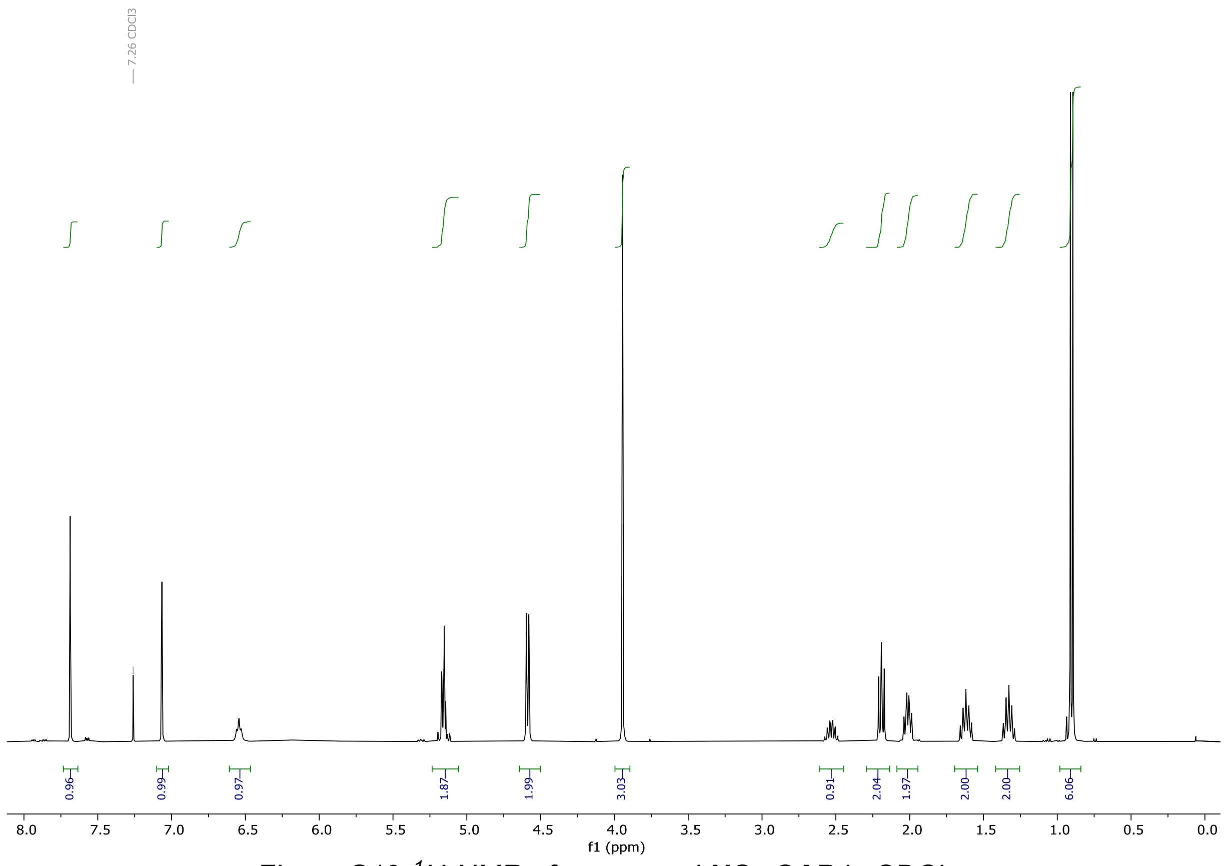

Figure S19: ^{1}H-NMR of compound **NO$_2$-CAP** in $CDCl_3$.

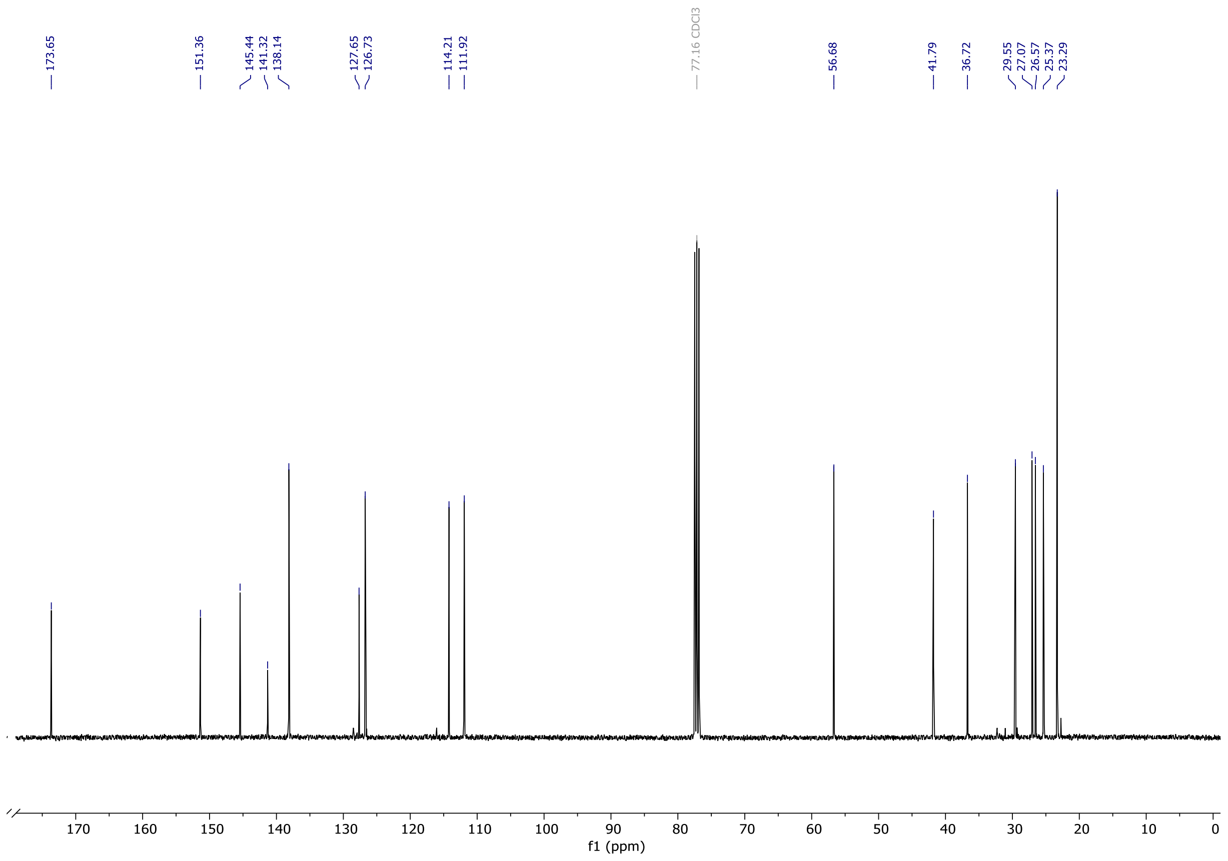

Figure S20: ^{13}C-NMR of compound **NO$_2$-CAP** in $CDCl_3$.

N-(4-Hydroxy-5-methoxy-2-nitrobenzyl)-8-methylnonanamide (NO_2-DHCAP)

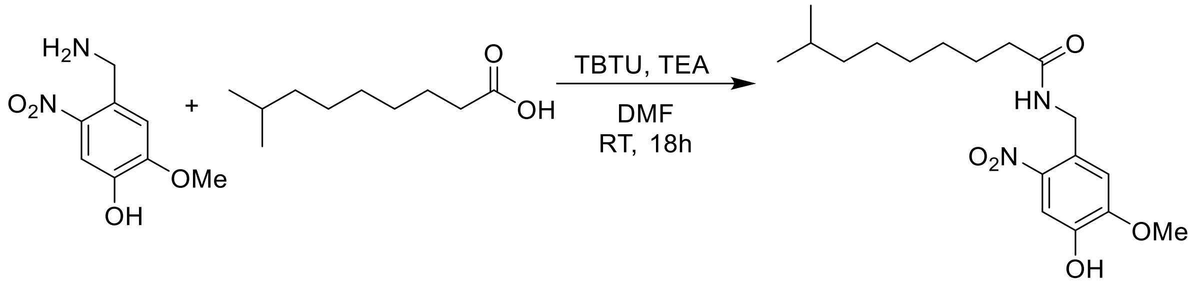

In dry DMF (40 mL), 8-methylnonanoic acid (1.52 g, 8.82 mmol, 1.0 eq.), 4-(aminomethyl)-2-methoxy-5-nitrophenol (2.0 g, 10.58 mmol, 1.2 eq.), TBTU (3.4 g, 10.58 mmol, 1.2 eq.) and TEA (3.7 mL, 26.44 mmol, 3.0 eq.) were dissolved at 0°C. The reaction mixture was stirred for 16 h. Afterwards, the mixture was quenched with brine (50 mL) and the pH was changed to slightly acidic with aq. HCl (6 M). To the solution, water (50 mL) was added and extracted with EtOAc (3 x 100 mL). The organic phase was dried over magnesium sulfate and the solvent was removed under reduced pressure. The crude material was further purified via column chromatography on silica (pentane: EtOAc 1:1) and recrystallisation (2:1 Hexane: Et_2O) to yield the final product (380 mg, 1.1 mmol, 16%) as slightly yellow solid.

^{1}H-NMR (400 MHz, $CDCl_3$) δ 7.69 (s, 1H), 7.07 (s, 1H), 6.53 (t, *J* = 6.5 Hz, 1H), 4.59 (d, *J* = 6.5 Hz, 2H), 3.94 (s, 3H), 2.24 – 2.13 (m, 2H), 1.59 (dd, *J* = 15.6, 6.3 Hz, 2H), 1.48 (dp, *J* = 13.2, 6.6 Hz, 1H), 1.32 – 1.18 (m, 6H), 1.15 – 1.06 (m, 2H), 0.83 (d, *J* = 6.6 Hz, 6H).
^{13}C-NMR (101 MHz, $CDCl_3$) δ 173.7, 151.3, 145.4, 141.2, 127.6, 114.2, 111.8, 56.6, 41.7, 38.9, 36.7, 29.6, 29.3, 27.9, 27.2, 25.7, 22.6.
HR-MS (ESI): m/z calc. for $C_{18}H_{28}N_2O_5Na$ $[M+Na]^+$: 375.1890, found: 375.1898.
MS (ESI): m/z (%) = 375.2 (100) $[M+Na]^+$.
IR (ATR) [cm^{-1}]: 2920, 1644, 1592, 1521, 1497, 1325, 1273, 1207, 1054, 882, 809.

Mp.: 100.3–101.5 °C

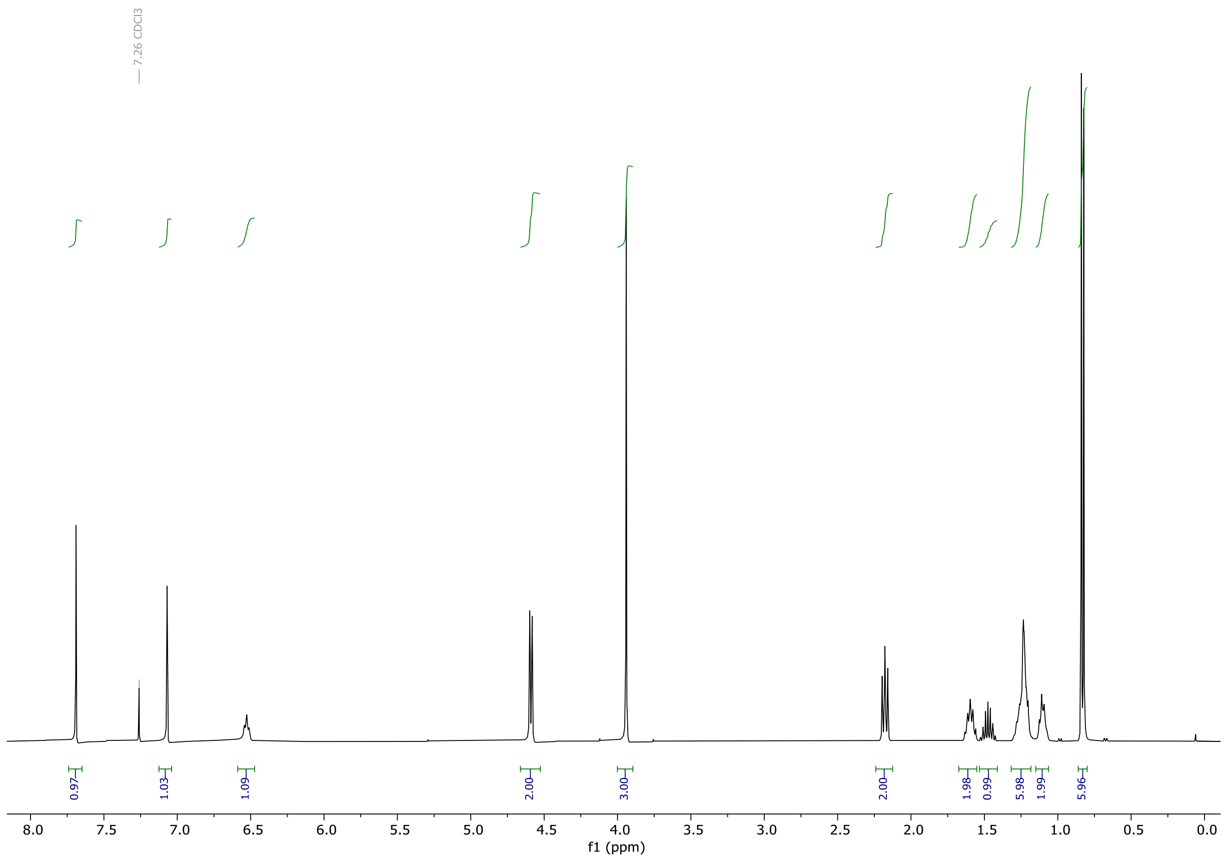

Figure S21: ^{1}H-NMR of compound **NO_2-DHCAP** in $CDCl_3$.

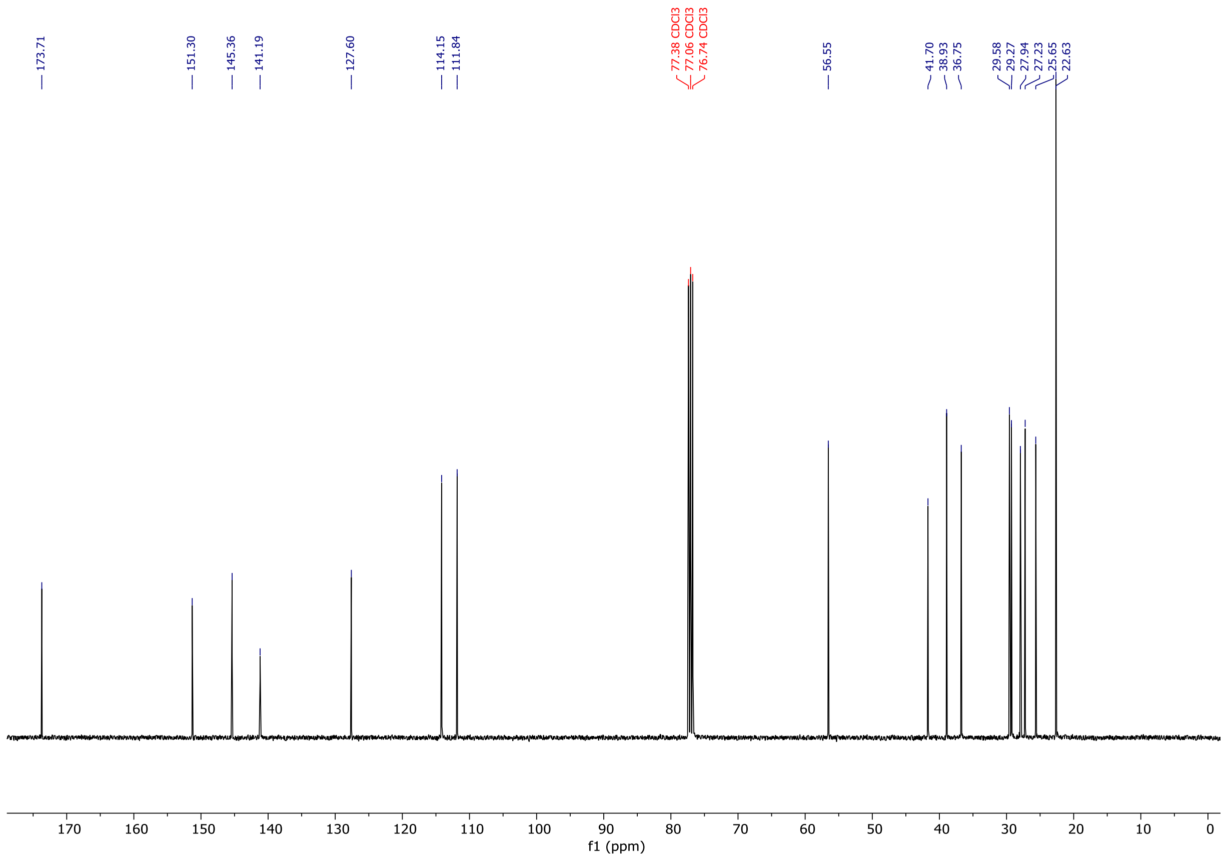

Figure S22: ^{13}C-NMR of compound **NO_2-DHCAP** in $CDCl_3$.

N-(4-Hydroxy-5-methoxy-2-nitrobenzyl)nonanamide (NO_2-NV)

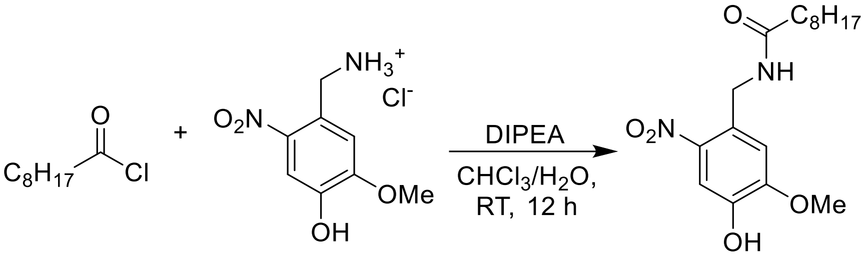

15 **NO_2-NV**

To dry DMF (5 mL), **15** (468 mg, 2.0 mmol, 1.0 eq.) and DIPEA (680 µL, 4.0 mmol, 2.0 eq.) were added and stirred for 15 minutes. Afterwards, nonanoyl chloride (360 µL, 2.0 mmol, 1.0 eq.) was added and the reaction mixture was stirred for 12 h. The reaction mixture was diluted with water (30 mL) and extracted with DCM (3 x 30 mL). The organic phase was dried with magnesium sulfate and the solvent was removed *in vacuo* to yield the crude product, which was purified by recrystallisation from MTBE/ heptane (8:1) to yield the title product (190 mg, 0.56 mmol, 28%) as yellow crystals.

^{1}H-NMR (300 MHz, $CDCl_3$): δ = 7.69 (s, 1H), 7.07 (s, 1H), 6.52 (t, *J* = 6.5 Hz, 1H), 4.59 (d, *J* = 6.5 Hz, 2H), 3.94 (s, 3H), 2.17 (dd, *J* = 8.3 Hz) 1.64 - 1.55 (m, 2H), 1.31 - 1.17 (m, 10H), 0.85 (t, *J* = 6.9 Hz, 3H).

^{13}C-NMR (75 MHz, $CDCl_3$): δ = 173.7, 151.3, 145.4, 141.2, 127.6, 114.2, 111.8, 77.3, 56.6, 41.7, 36.8, 31.8, 29.3, 29.2, 29.1, 25.6, 22.6, 14.1.

HR-MS (ESI): m/z calc. for $C_{17}H_{26}N_2O_5$ $[M-H]^-$: 337.1769, found: 337.1770.

MS (ESI): m/z (%) = 337.2 $[M-H]^-$.

IR (ATR) [cm^{-1}]: 2919, 2849, 2363, 2339, 1650, 1521, 1322, 1273, 1240, 1210.

Mp.: 92–95 °C.

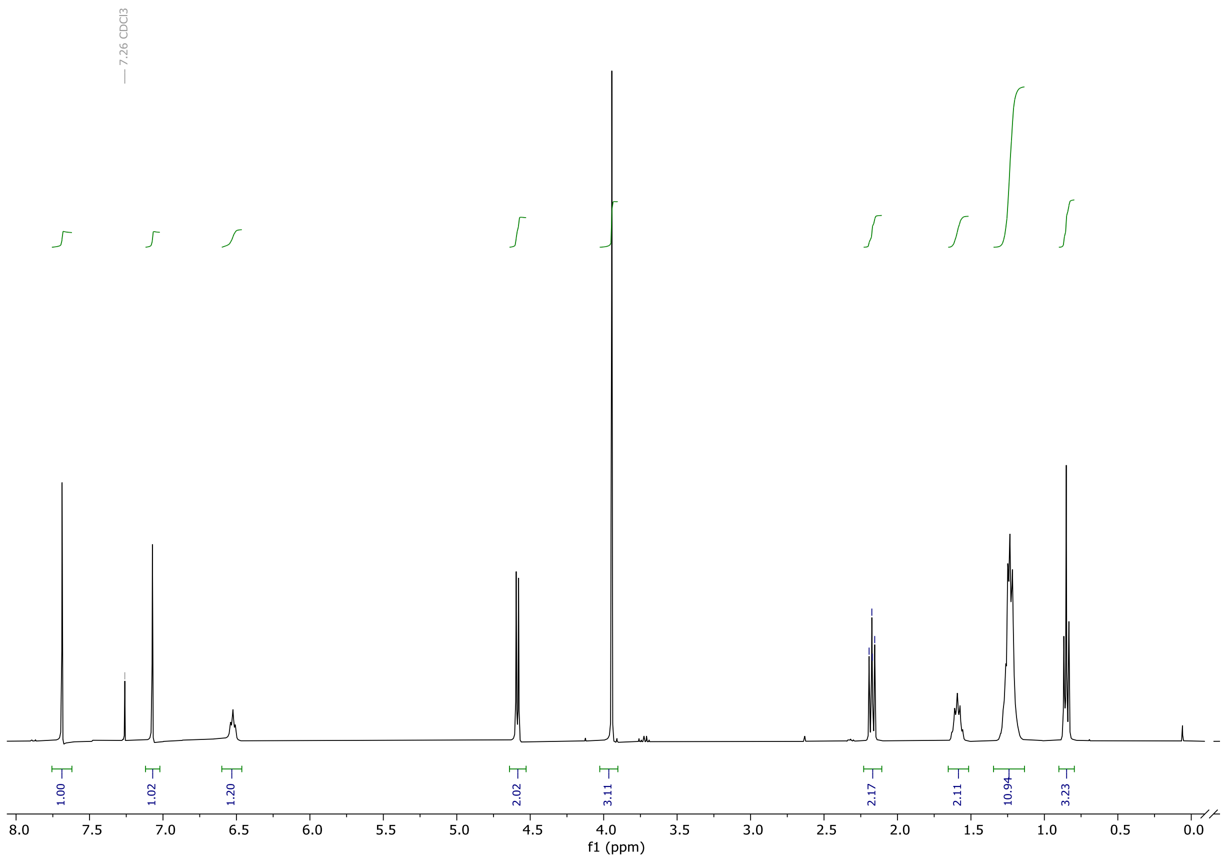

*Figure S23: ^{1}H-NMR of compound **NO_2-NV** in $CDCl_3$.*

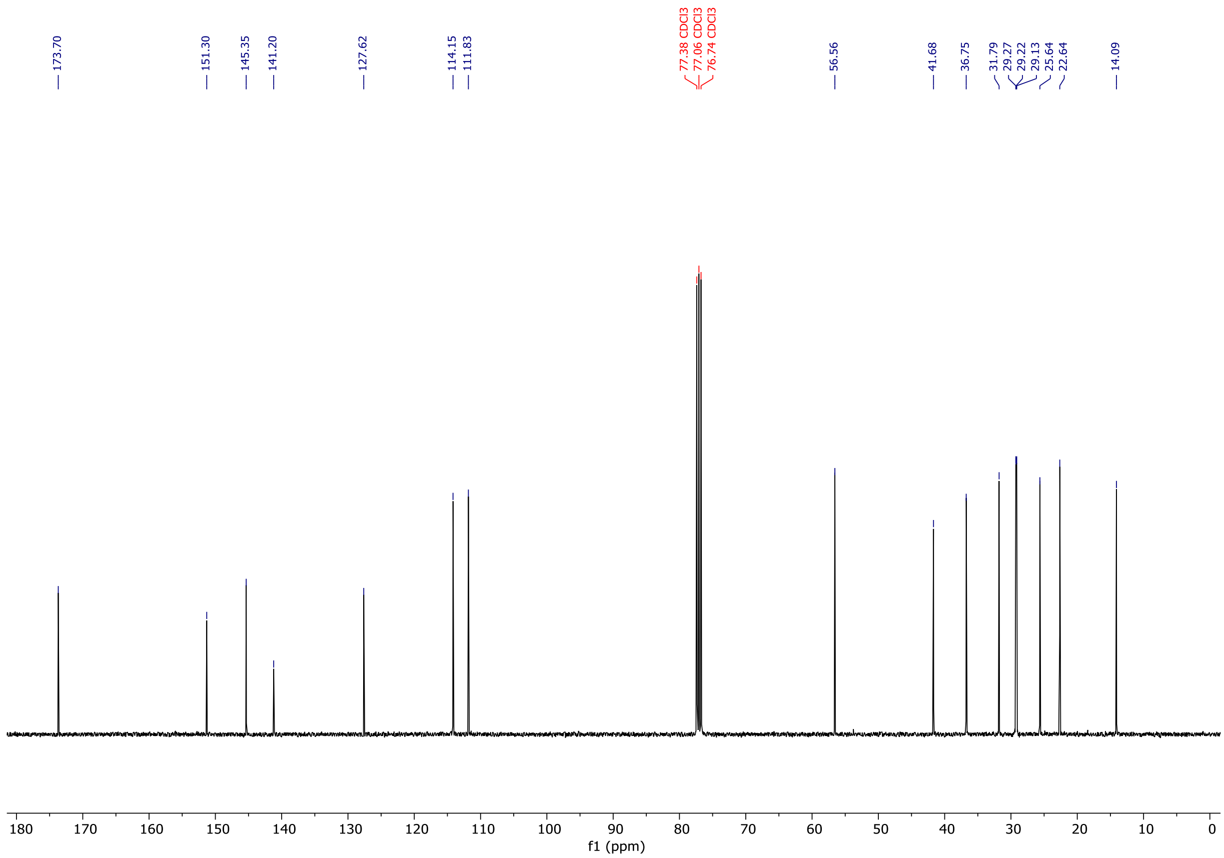

*Figure S24: ^{13}C-NMR of compound **NO_2-NV** in $CDCl_3$.*

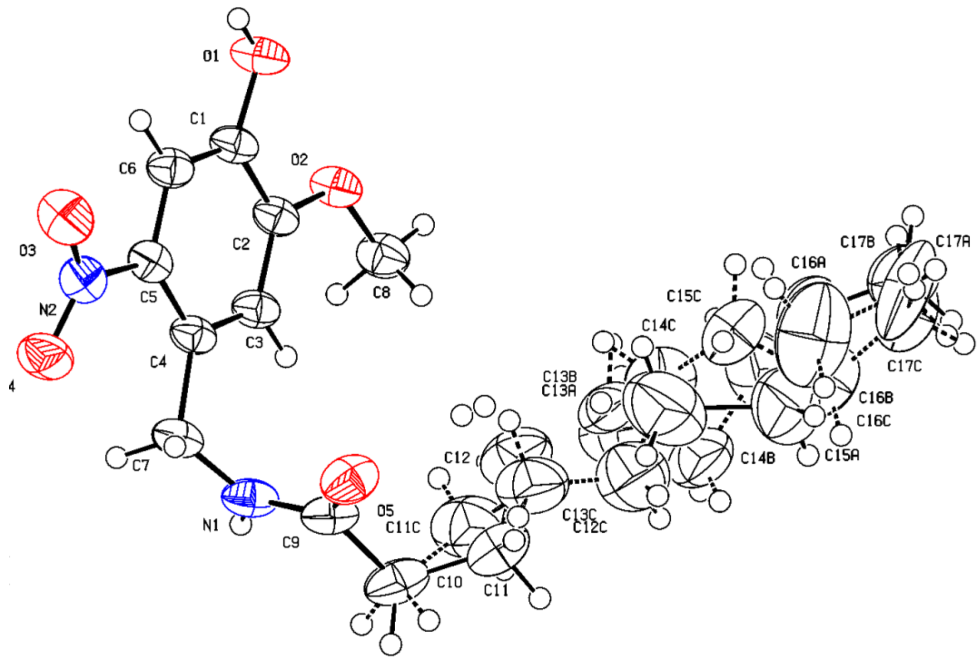

*Figure S25: ORTEP plot of the X-ray structure of **NO_2-NV** at 50% probability of ellipsoid level.*

(E)-N-(4-hydroxy-5-methoxy-2-nitrobenzyl)-8-methylnon-6-enamide (NO_2-CAP)

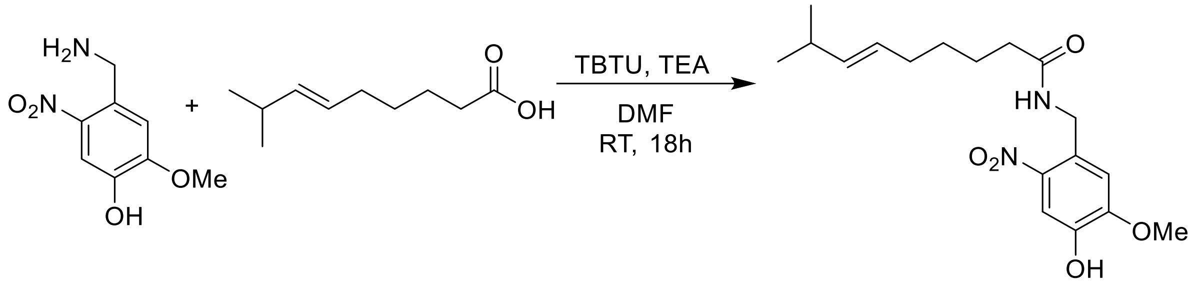

In dry DMF (40 mL), (*E*)-8-methylnon-6-enoic acid (1.13 g, 6.7 mmol, 1.0 eq.), 4-(aminomethyl)-2-methoxy-5-nitrophenol (1.87 g, 8.0 mmol, 1.2 eq.), TBTU (2.57 g, 8.0 mmol, 1.2 eq.) and TEA (2.8 mL, 20.0 mmol, 3.0 eq.) were dissolved at 0°C. The reaction mixture was stirred for 16 h. Afterwards, the mixture was quenched with brine (50 mL) and the pH was changed to slightly acidic with aq. HCl (6 M). To the solution, water (50 mL) was added and extracted with EtOAc (3 x 100 mL). The organic phase was dried over magnesium sulfate and the solvent was removed under reduced pressure. The crude material was further purified via column chromatography on silica (pentane: EtOAc 1:1) and recrystallisation (2:1 hexane: Et_2O) to yield the title compound (490 mg, 1.4 mmol, 22%) as shiny yellow crystals.

^{1}H-NMR (400 MHz, $CDCl_3$) δ 7.69 (s, 1H), 7.07 (s, 1H), 6.54 (t, *J* = 6.5 Hz, 1H), 5.23 – 5.06 (m, 2H), 4.59 (d, *J* = 6.5 Hz, 2H), 3.94 (s, 3H), 2.53 (ddt, *J* = 13.2, 8.7, 6.6 Hz, 1H), 2.29 – 2.14 (m, 2H), 2.01 (dddd, *J* = 7.4, 5.7, 4.4, 1.3 Hz, 2H), 1.70 – 1.54 (m, 2H), 1.42 – 1.25 (m, 2H), 0.90 (d, *J* = 6.6 Hz, 6H).

^{13}C-NMR (101 MHz, $CDCl_3$) δ 173.5, 151.3, 145.3, 141.2, 138.0, 127.6, 126.6, 114.1, 111.8, 56.6, 41.7, 36.6, 29.5, 27.0, 26.5, 25.3, 23.2.

HR-MS (ESI): m/z calc. for $C_{18}H_{26}N_2O_5Na$ $[M+Na]^+$: 373.1734 found: 373.1746.

MS (ESI): m/z (%) = 373.2 (100) $[M+Na]^+$.

IR (ATR) [cm^{-1}]: 1644, 1591, 1525, 1460, 1322, 1280, 1237, 1207, 1057, 879.

Mp.: 92.8 – 93.2°C

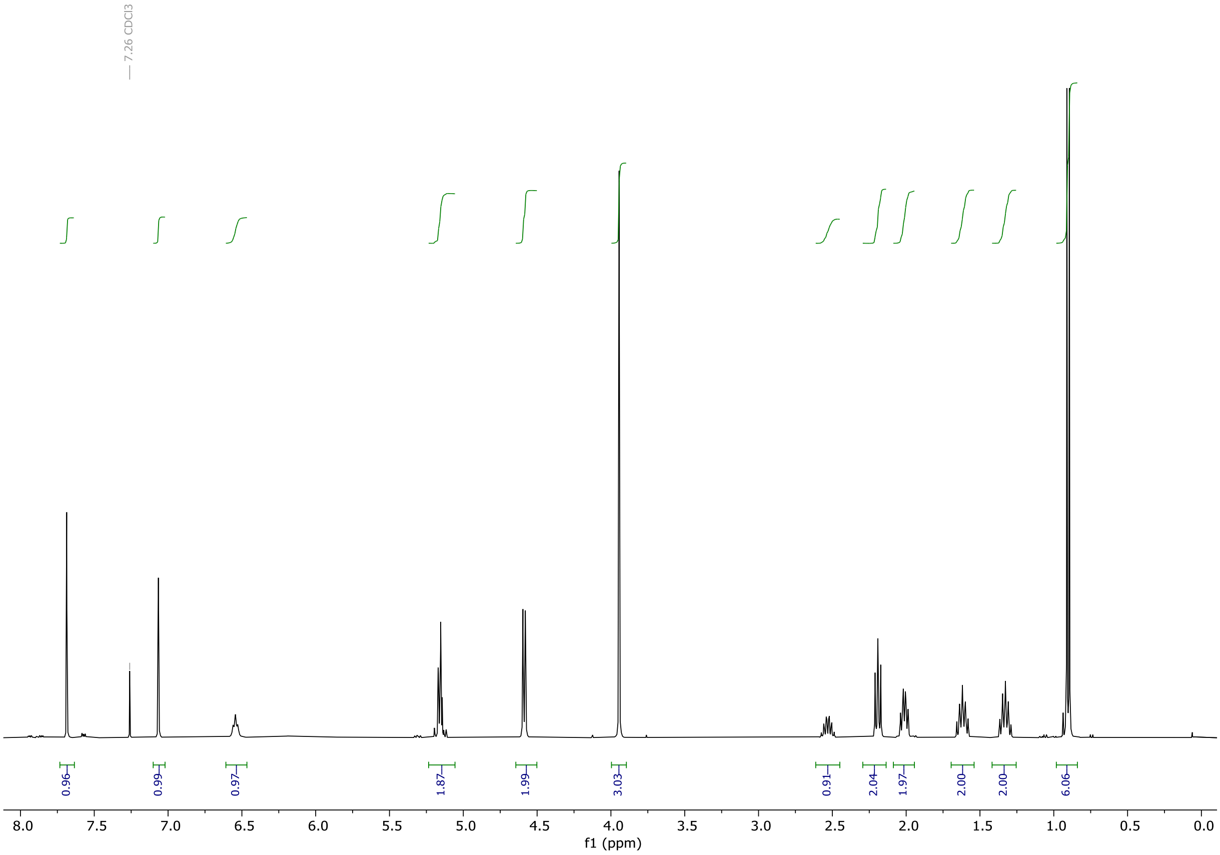

*Figure S28: ^{1}H-NMR of compound **CAP** in $CDCl_3$.*

(*E*)-*N*-(4-((4,5-Dimethoxy-2-nitrobenzyl)oxy)-3-methoxybenzyl)-8-methylnon-6-enamide (ONB-CAP)

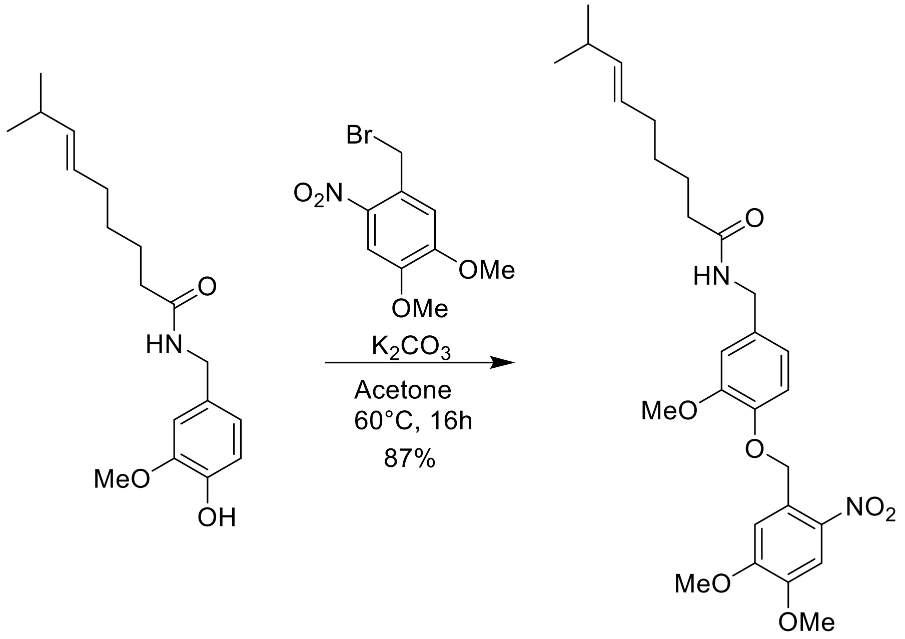

CAP (300 mg, 1 mmol, 1.0 eq.) and 1-(bromomethyl)-4,5-dimethoxy-2-nitrobenzene (324 mg, 1.2 mmol, 1.2 eq.) were dissolved in acetone (5 mL) and potassium carbonate (280 mg, 2.0 mmol, 2 eq.) was added. The mixture was stirred at 60°C for 16 h. Afterwards, the solvent was removed under reduced pressure and the residue was dissolved in water (10 mL) and ethyl acetate (20 mL). The aqueous layer was extracted with ethyl acetate (3 x 10 mL) and the combined organic layer was dried over magnesium sulfate. The solvent was removed under reduced pressure and the crude material was purified via column chromatography (silica, 50→100% EtOAc in pentane). The final compound (474 mg, 87%, 0.87 mmol) was yielded as yellowish solid.

^{1}H-NMR (400 MHz, $CDCl_3$) δ 7.72 (d, *J* = 4.1 Hz, 1H), 7.46 (d, *J* = 2.6 Hz, 1H), 6.87 – 6.81 (m, 2H), 6.75 (m, 1H), 5.98 – 5.80 (m, 1H), 5.55 – 5.45 (m, 2H), 5.25 – 5.04 (m, 2H), 4.35 (dd, *J* = 5.8, 3.8 Hz, 2H), 3.96 – 3.91 (m, 6H), 3.86 (d, *J* = 3.5 Hz, 3H), 2.65 – 2.43 (m, 1H), 2.20 (td, *J* = 7.7, 2.2 Hz, 2H), 2.10 – 1.95 (m, 2H), 1.73 – 1.54 (m, 2H), 1.44 – 1.31 (m, 2H), 0.90 (dd, *J* = 6.7, 2.7 Hz, 6H).

^{13}C-NMR (101 MHz, $CDCl_3$) δ 172.9, 154.0, 149.9, 149.9, 147.8, 147.0, 147.0, 138.9, 138.0, 132.5, 129.8, 126.7, 120.2, 114.5, 114.5, 111.8, 109.5, 107.9, 68.4, 68.4, 56.4, 56.0, 43.4, 43.3, 36.7, 29.6, 27.0, 26.5, 25.5, 23.2.
HR-MS (ESI): m/z calc. for $C_{27}H_{36}N_2O_7Na$ $[M+Na]^+$: 523.2415 found: 523.2421.
MS (ESI): m/z (%) = 523.3 (100) $[M+Na]^+$.

Mp: 123.1 °C

IR (ATR) [cm^{-1}]:2162, 1635, 1512, 1457, 1325, 1273, 1216, 1133, 1069, 1033, 794.

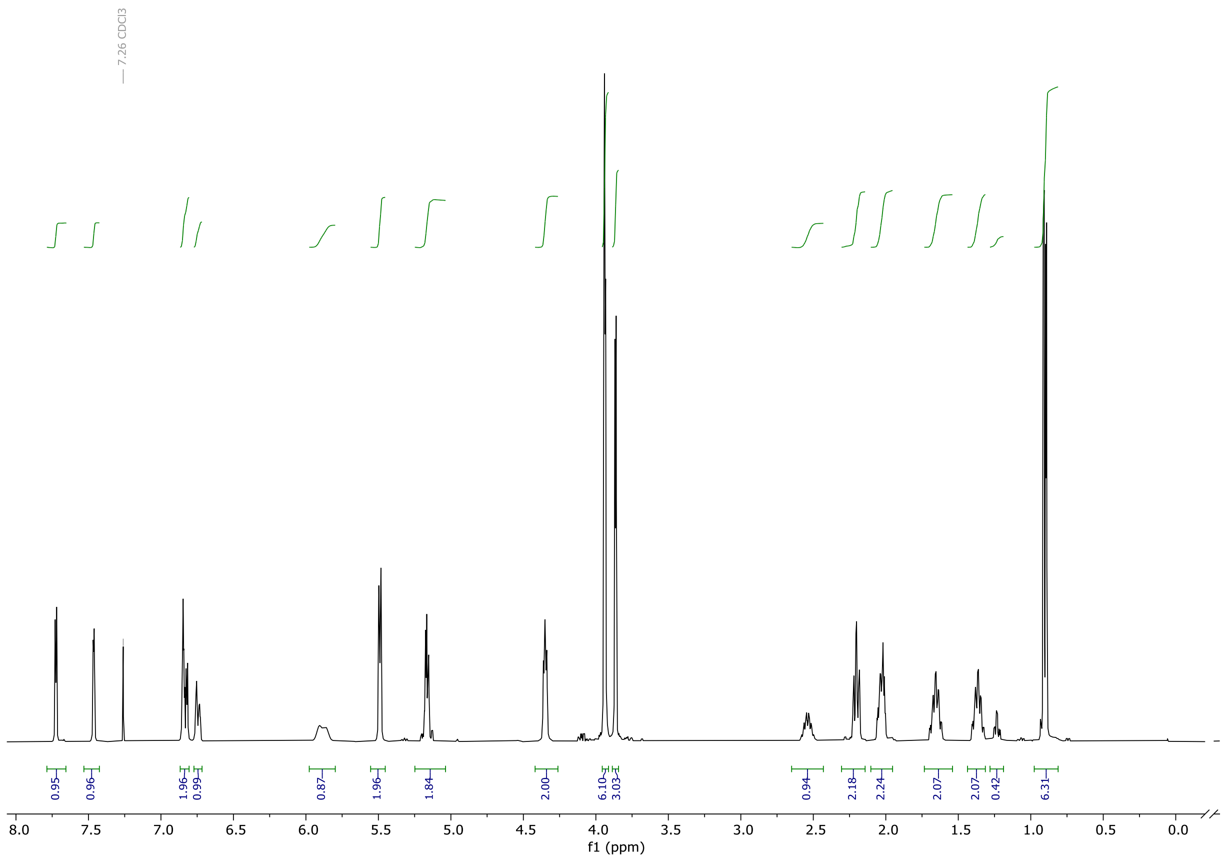

Figure S26: ^{1}H-NMR of compound ***ONB-CAP*** *in $CDCl_3$.*

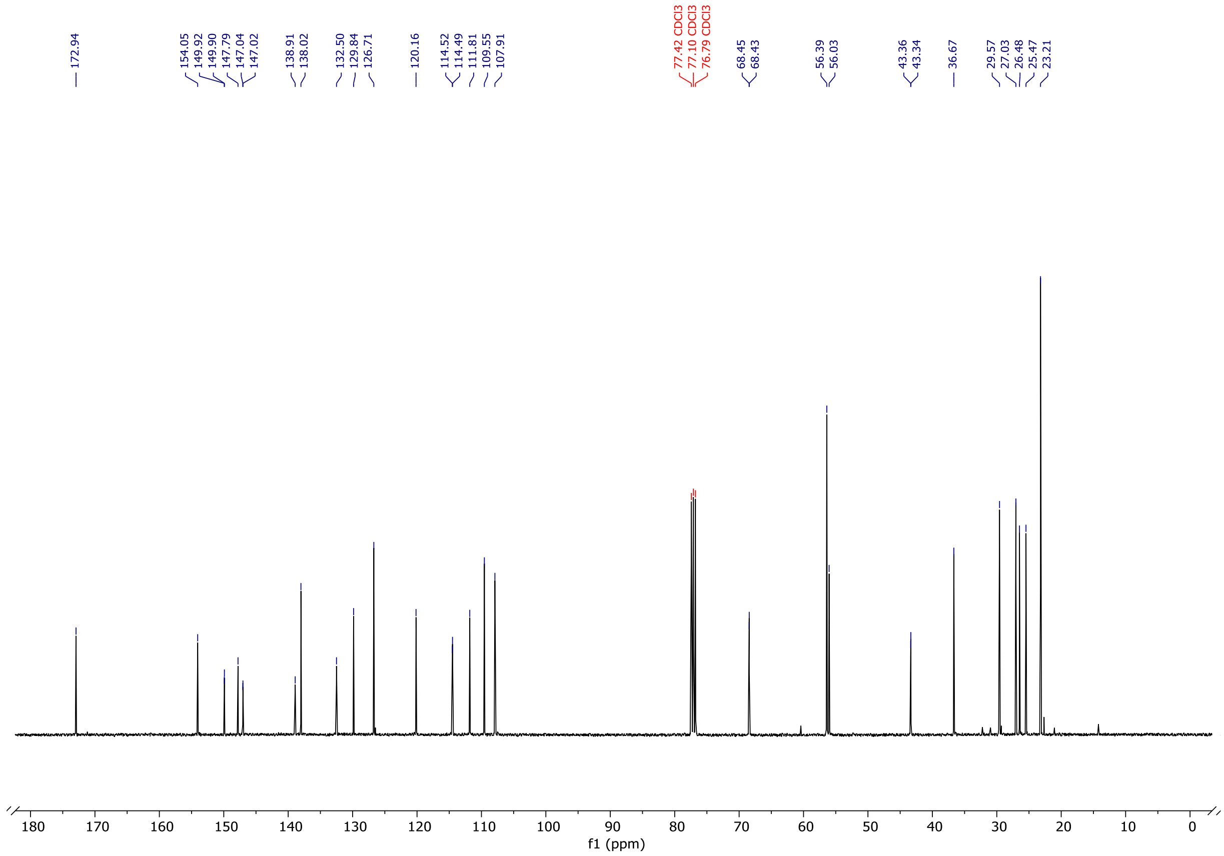

Figure S27: ^{1}H-NMR of compound ***ONB-CAP*** *in $CDCl_3$*

***N*-(4-((7-(diethylamino)-2-oxo-2H-chromen-4-yl)methoxy)-5-methoxy-2-nitrobenzyl)-nonanamide (DEAC-NO_2-NV)**

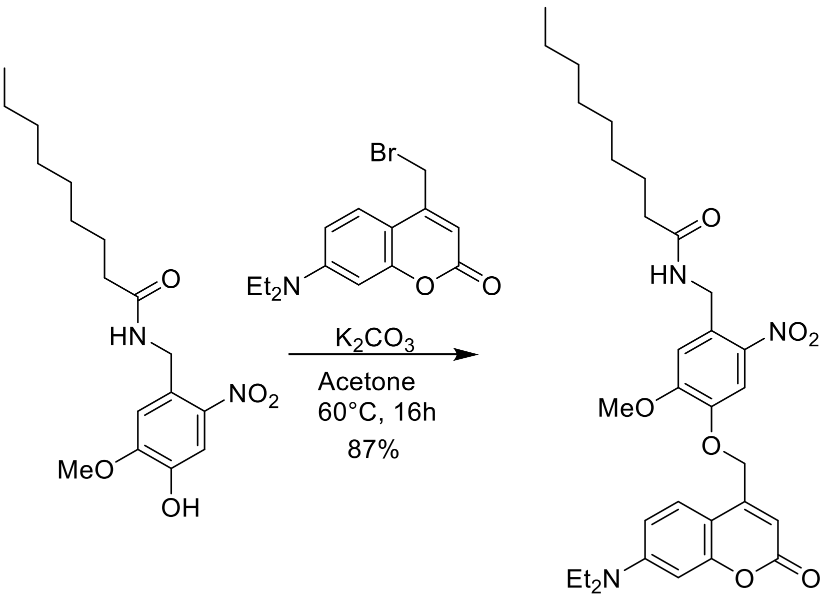

The title compound was synthesized analogously to **ONB-CAP** (*vide supra*) starting from **NO_2-NV** (30 mg, 1.0 eq.) and 4-(bromomethyl)-7-(diethylamino)-2H-chromen-2-one (1.2 eq.). An analytical fraction was purified via column chromatography (silica, 50→100% EtOAc in pentane) to give a yellow solid. The yield was not determined.

^{1}H-NMR (400 MHz, $CDCl_3$) δ 7.72 (s, 1H), 7.42 (d, *J* = 9.0 Hz, 1H), 7.31 (s, 2H), 7.20 (s, 1H), 6.70 (d, *J* = 9.1 Hz, 1H), 6.62 (d, *J* = 2.6 Hz, 1H), 6.44 (t, *J* = 6.7 Hz, 1H), 6.34 (s, 1H), 5.30 (s, 2H), 4.68 (d, *J* = 6.4 Hz, 2H), 4.05 (s, 3H), 3.48 (q, *J* = 7.1 Hz, 4H), 2.22 (t, *J* = 7.7 Hz, 2H), 1.29 (dq, *J* = 13.7, 5.7 Hz, 19H), 0.91 (t, *J* = 6.6 Hz, 3H).

^{13}C-NMR (101 MHz, $CDCl_3$) δ 173.4, 161.8, 156.3, 154.4, 149.0, 146.4, 140.4, 130.3, 124.4, 115.0, 110.4, 66.9, 56.7, 45.2, 41.6, 36.7, 31.8, 29.3, 29.3, 29.2, 25.6, 22.7, 14.1, 12.4.

HR-MS (ESI): m/z calc. for $C_{31}H_{41}N_3O_7$ $[M+H]^+$: 568.3017 found: 568.3024.
m/z calc. for $C_{31}H_{40}N_3O_7Na$ $[M+Na]^+$: 590.2837 found: 590.2840.

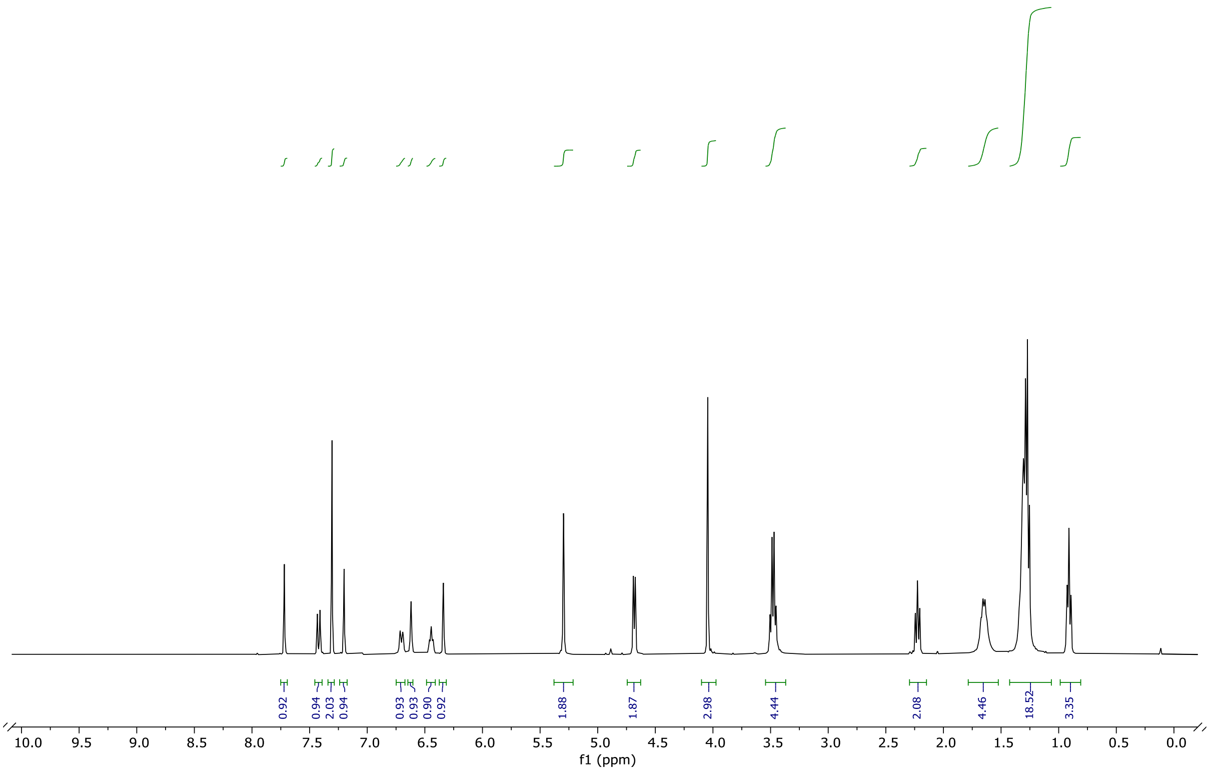

*Figure S28: ^{1}H-NMR of compound **DEAC-NO$_2$-NV** in CDCl$_3$.*

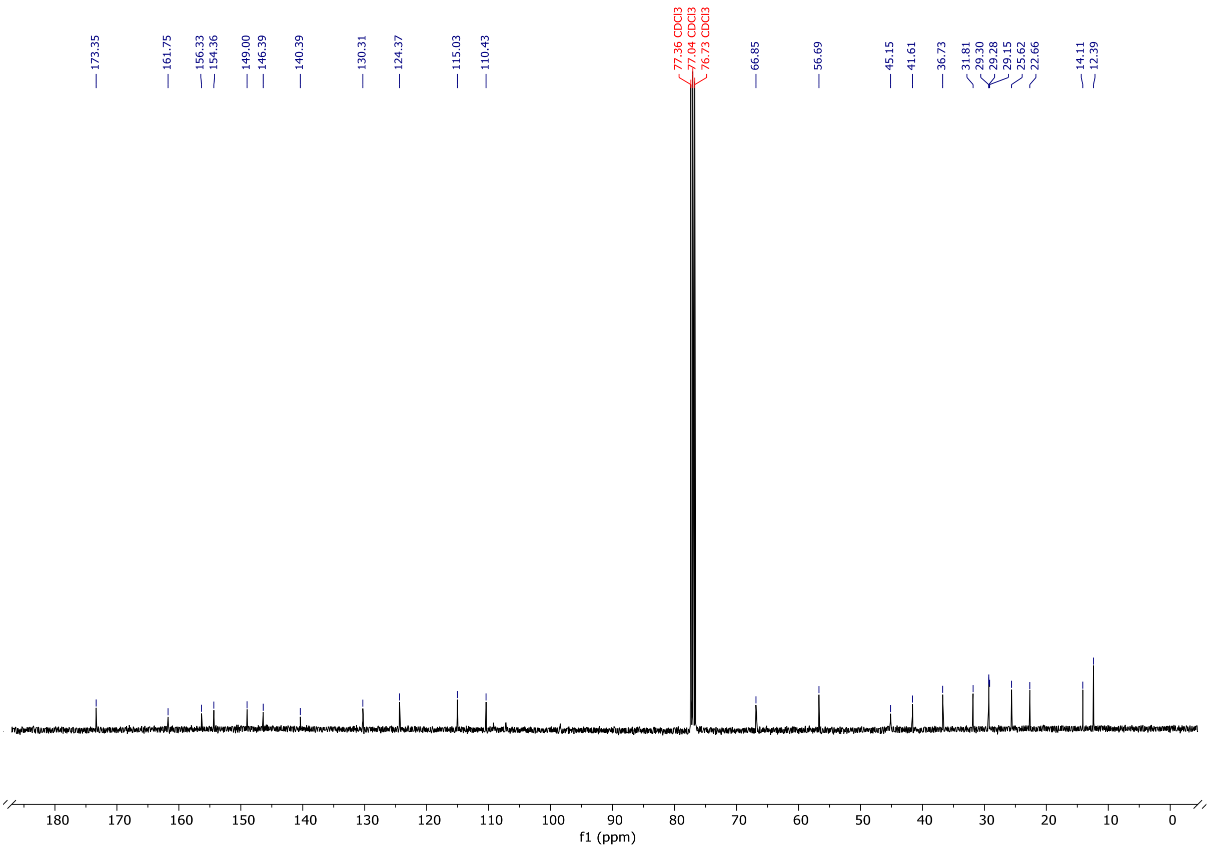

Figure S29: ^{1}H-NMR of compound **DEAC-NO$_2$-NV** in CDCl$_3$.

3. Photophysical and Photochemical Characterization of Light-Sensitive Capsaicinoids

Chemical actinometry

A modification of a standard protocol was applied for the determination of the photon flux.[5] An aqueous H_2SO_4 solution (0.05 M) containing freshly recrystallized $K_3[Fe(C_2O_4)_3]$ (41 mM, 2 mL) was irradiated at 20 °C for a given period of time in the dark with a 365 nm and 405 nm LED at a controlled, fixed, low intensity. The solution was then diluted with 1.0 mL of an aqueous H_2SO_4 solution (0.5 M) containing phenanthroline (1 g/L) and NaOAc (122.5 g/L) and left to react for 10 min. The absorption at 510 nm was measured and compared to an identically prepared non-irradiated sample. The concentration of $[Fe(phenanthroline)_3]^{2+}$ complex was calculated using its molar absorptivity (ε = 11100 M^{-1} cm^{-1}) and considering the dilution. The quantity of Fe^{2+} ions expressed in mol was plotted against time and the slope, obtained by linear fitting the data points to the equation y = ax +b, equals the rate of formation of the Fe^{2+} ion at the given wavelengths. The rate can be converted into the photon flux (I) by dividing it by the quantum yield of $[Fe(phenanthroline)_3]^{2+}$ complex (Φ_{365nm} = 1.29). The obtained photon fluxes were I_{365nm} = 2.38122 x 10^{-5} mE s^{-1}.

Quantum yield

The quantum yield of the photochemical ring opening is determined using an initial slope method. The sample is irradiated with an LED and the natural logarithm of the absorbance data is plotted against time. From equation 1,[5] we can determine the quantum yield of the photochemical ring opening.

Eq. 1 $$\phi = \frac{-k\,[X]_0\;\;V}{I\;\;(1-10^{-Abs(0)})}$$

Where ϕ is the quantum yield; -*k* is the reaction rate; [X] is the concentration of the closed form in M^{-1}; *V* is the volume in mL and *I* is the photon flux in mE s^{-1}. Applying first order kinetics, the rate -*k* can be taken from the slope of the natural logarithm of absorbance vs. time.

3.1. NO_2-CAP

<table>
<tr><th>Absorption spectra in DMSO at different concentrations</th><th>Plot of the absorption against the concentration at λ_{360nm}</th></tr>
<tr><td>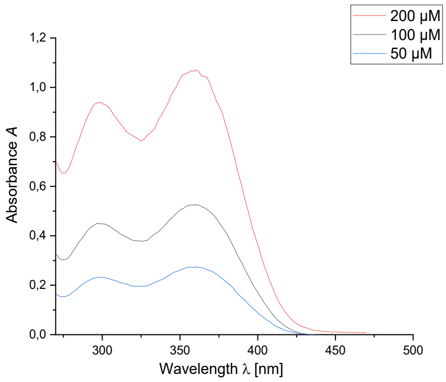
</td><td>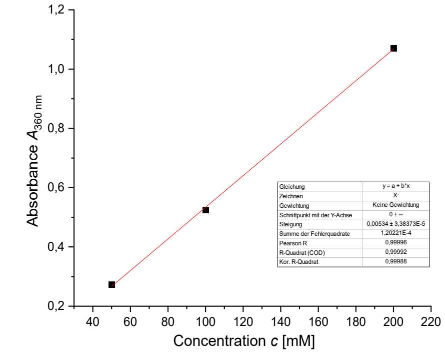
</td></tr>
<tr><td>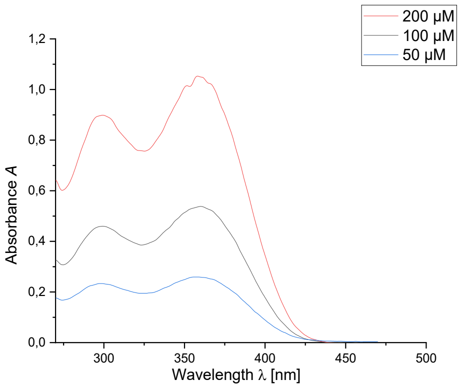
</td><td>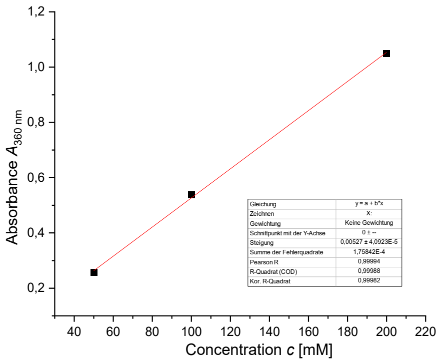
</td></tr>
<tr><td>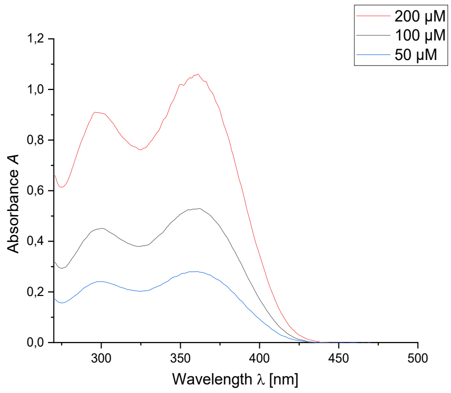
</td><td>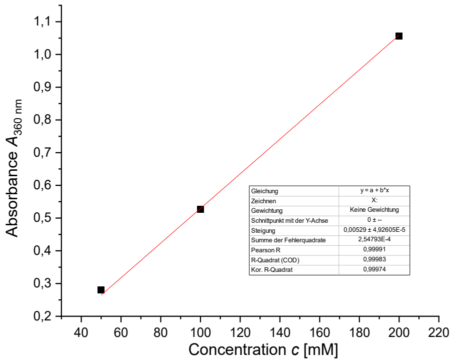
</td></tr>
</table>

Absorption spectra in DMSO at a concentration of c = 100 µM under irradiation with a 365 nm LED, measured every second	Plot of ln (A (λ_{365nm})) against the time t

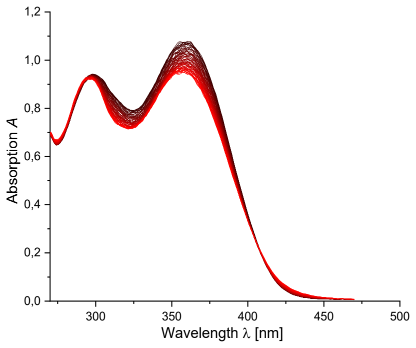

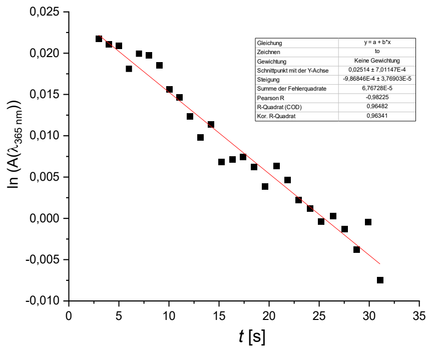

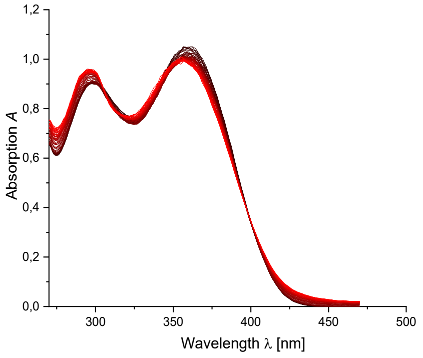

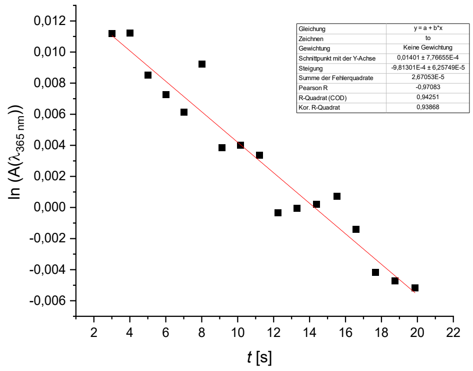

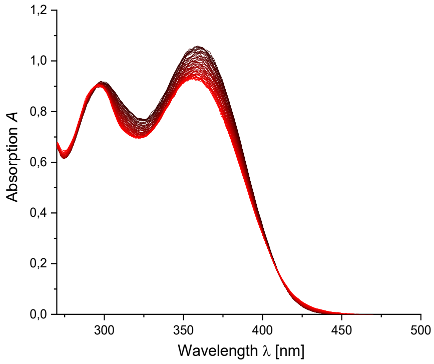

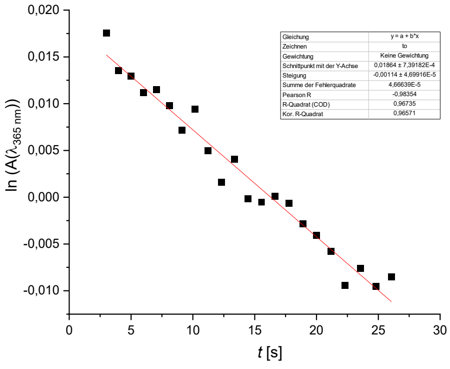

3.2. NO_2-DHCAP

Absorption spectra in DMSO at different concentrations	*Plot of the absorption against the concentration at λ_{360nm}*

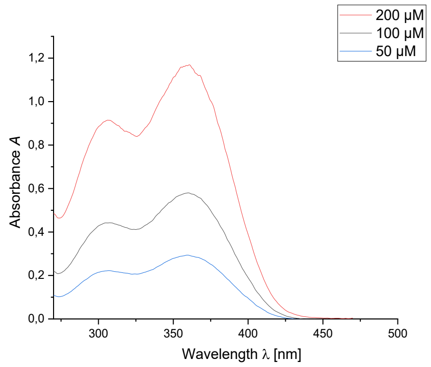

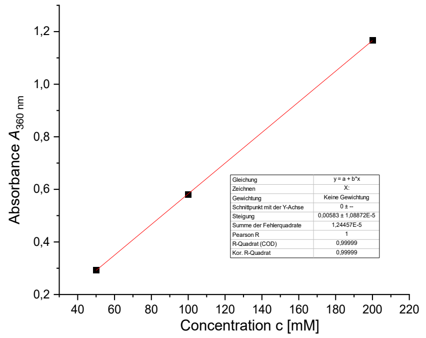

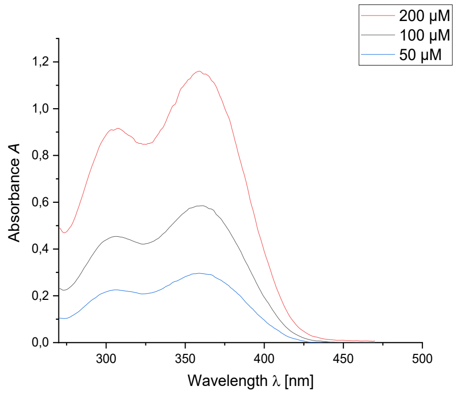

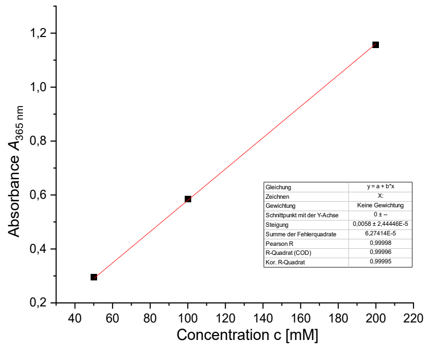

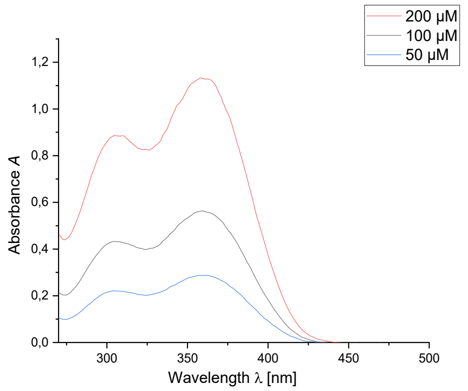

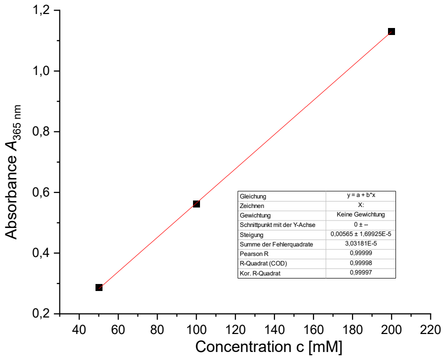

<table>
<tr><th>Absorption spectra in DMSO at a concentration of c = 100 μM under irradiation with a 365 nm LED, measured every second</th><th>Plot of ln (A (λ_{365nm})) against the time t</th></tr>
<tr><td>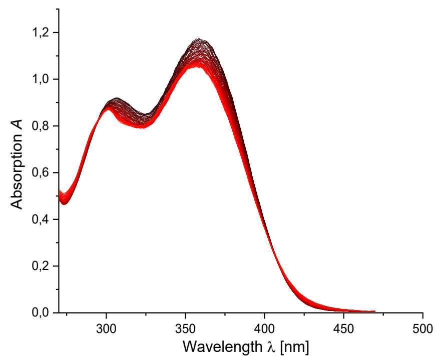
</td><td>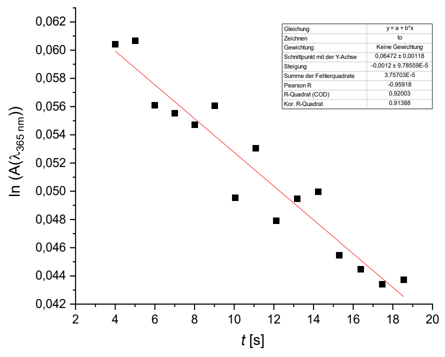
</td></tr>
<tr><td>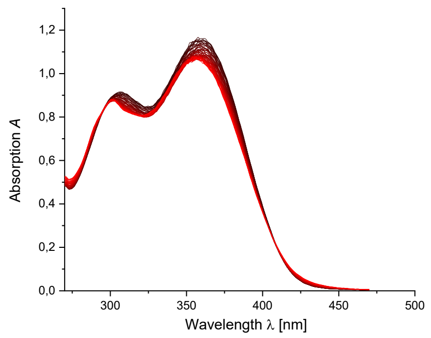
</td><td>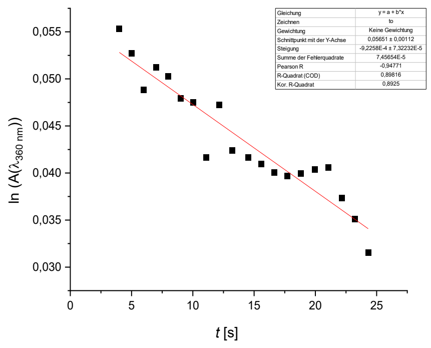
</td></tr>
<tr><td>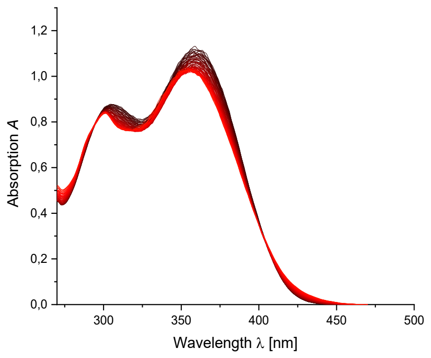
</td><td>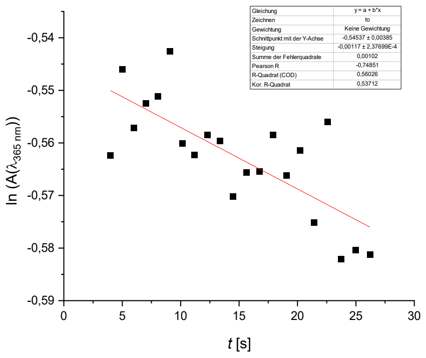
</td></tr>
</table>

3.3. NO_2-NV

<table>
<tr><th>Absorption spectra in DMSO at different concentrations</th><th>Plot of the absorption against the concentration at λ_{360nm}</th></tr>
<tr><td>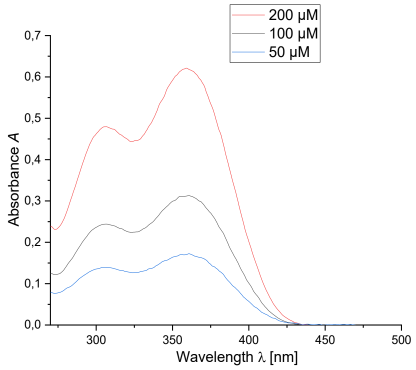
</td><td>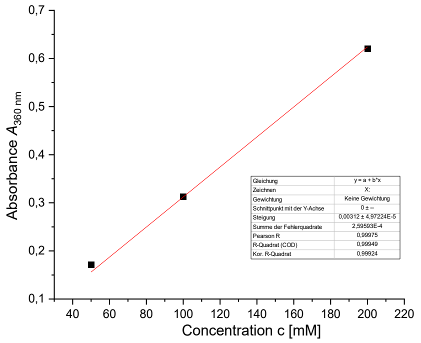
</td></tr>
<tr><td>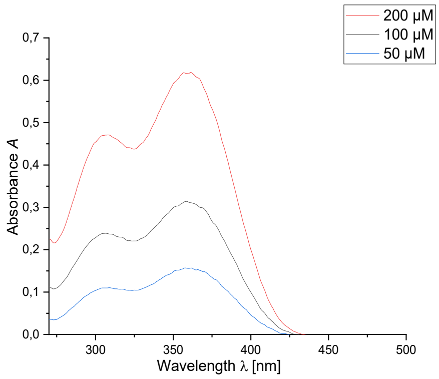
</td><td>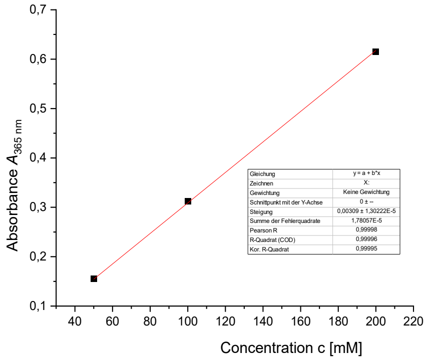
</td></tr>
<tr><td>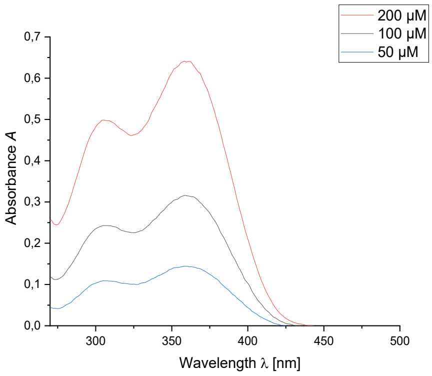
</td><td>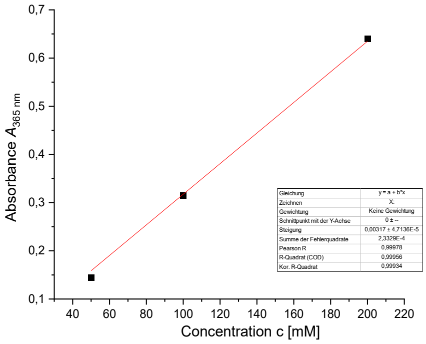
</td></tr>
</table>

<table>
<tr><th>Absorption spectra in DMSO at a concentration of c = 200 µM under irradiation with a 365 nm LED, measured every second</th><th>Plot of ln (A (λ_{365nm})) against the time t</th></tr>
<tr><td>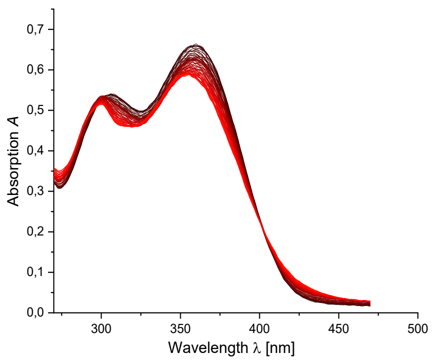
</td><td>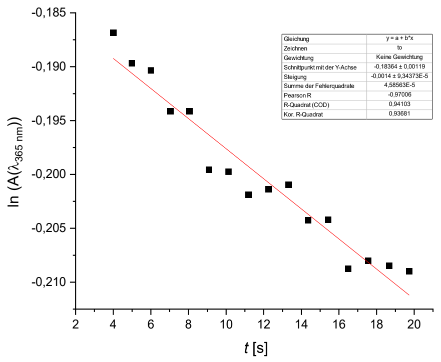
</td></tr>
<tr><td>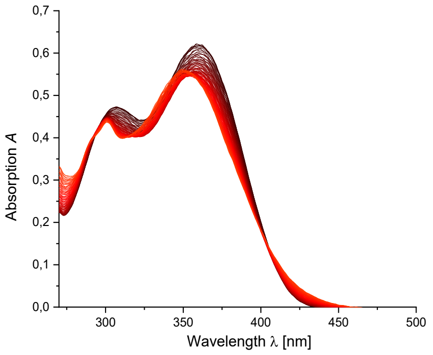
</td><td>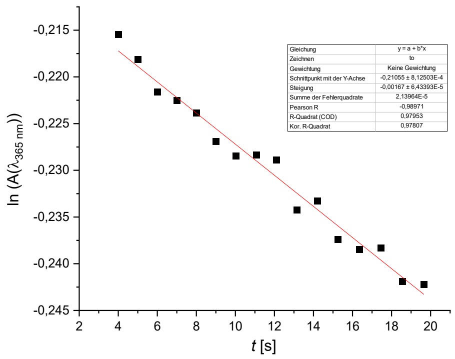
</td></tr>
<tr><td>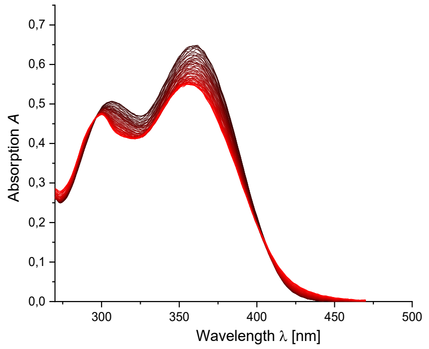
</td><td>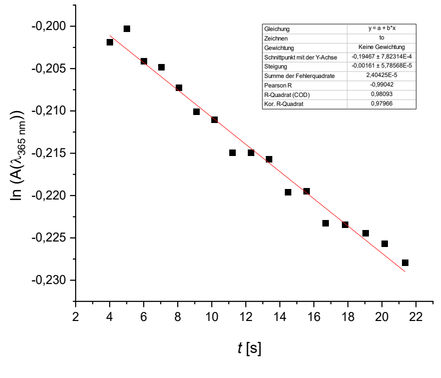
</td></tr>
</table>

3.4. ONB-CAP

<table>
<tr><th>Absorption spectra in DMSO at different concentrations</th><th>Plot of the absorption against the concentration at λ_{352nm}</th></tr>
<tr><td>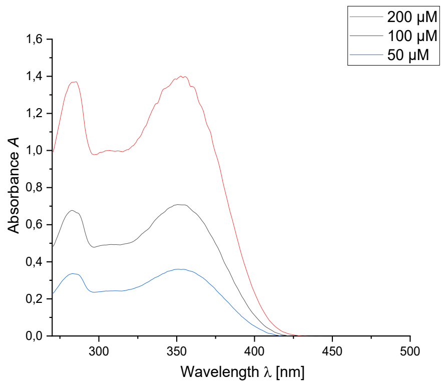
</td><td>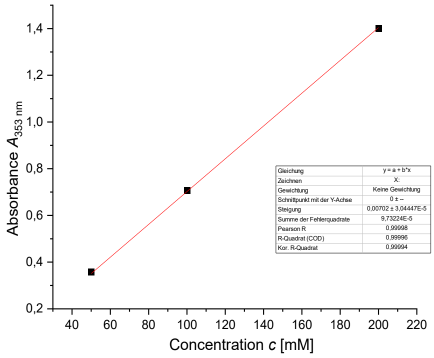
</td></tr>
<tr><td>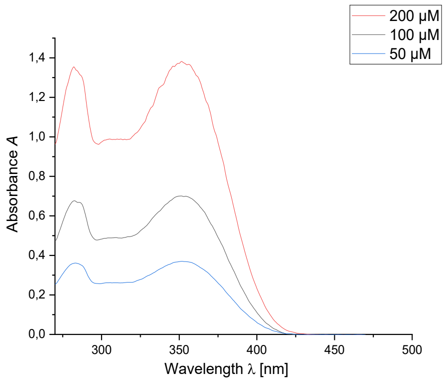
</td><td>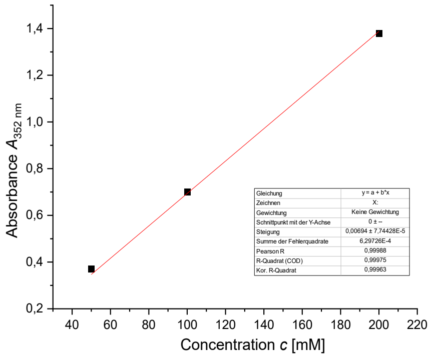
</td></tr>
<tr><td>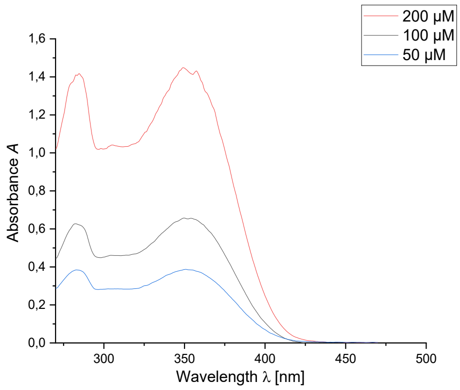
</td><td>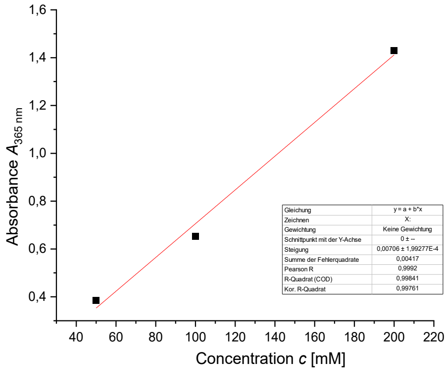
</td></tr>
</table>

<table>
<tr><th>Absorption spectra in DMSO at a concentration of c = 100 µM under irradiation with a 365 nm LED, measured every second</th><th>Plot of ln (A (λ_{400nm})) against the time t</th></tr>
<tr><td>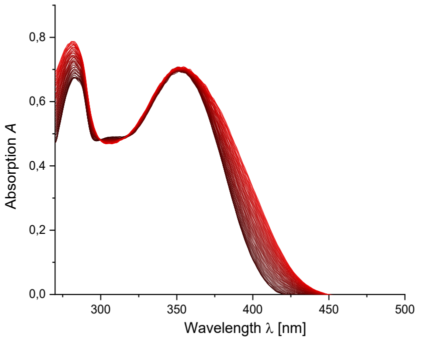
</td><td>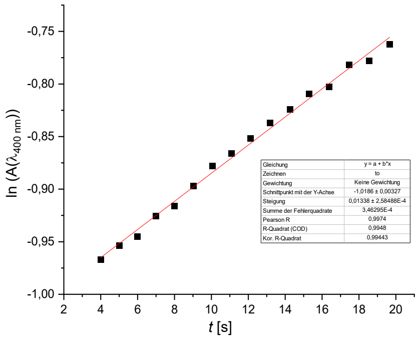
</td></tr>
<tr><td>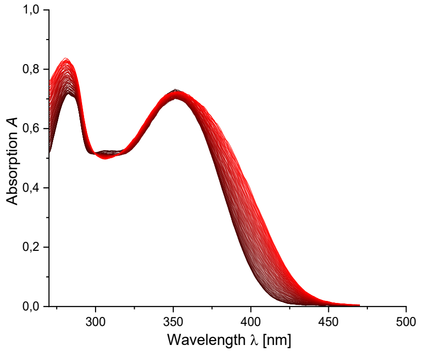
</td><td>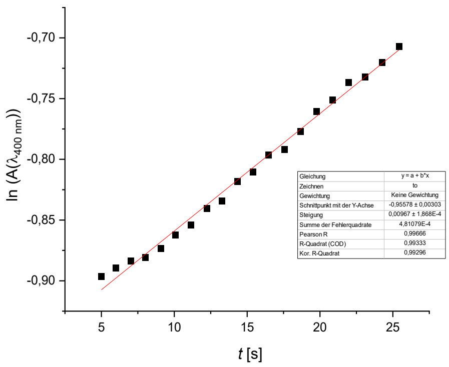
</td></tr>
<tr><td>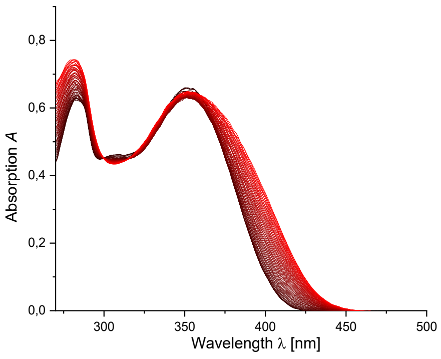
</td><td>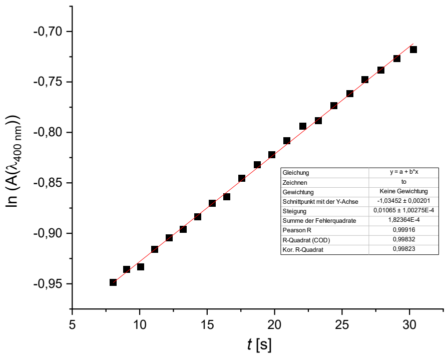
</td></tr>
</table>

3.5. HPLC Trances and NMR Spectra of the Photochemical Conversion of NO_2-NV

In Figure S30, HPLC-traces of **NO_2-NV** before and after irradiation with 365 nm light (ThorLabs 365 nm LED, full power) in a transparent HPLC vial for *ca*. 60 min (recoded at 220 nm in a gradient of 5→95% MeCN in water with 0.1% formic acid using an LC-MS).

Analysis of the fractions showed:

3.5–4.0 min: 230.1 (MH^+, 100%), 180.0 (90%) indicative for the ONB fragments (calculated exact mass of nitrosoaldehyde species: 181.04).

5.6 min: 158.1 (MH^+, 100%), indicative for nonivamide (calculated exact mass: 157.15).

Also, the MH^+ signal of **NO_2-NV** could still be detected; however, a qualification was due to the overlap of signals not feasible. Thus, we decided to also follow the reaction by ^{1}H-NMR spectroscopy.

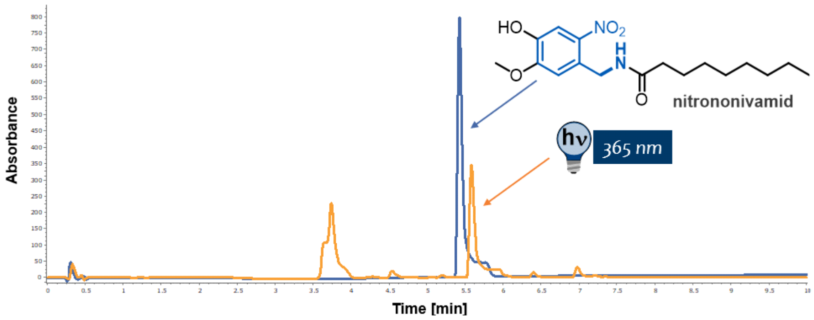

*Figure S30: HPLC traces of **NO_2-NV** before (blue) and after (orange) 365 nm irradiation.*

In Figure S31, ^{1}H-NMR spectra (400 MHz) of **NO_2-NV** before and at different times of irradiation with 365 nm light (10W, handheld torch) in a standard NMR tube (*ca*. 1.5 mg/mL). The figures show that **NO_2-NV** could be converted quantitatively into photoproducts. However, long irradiation times were required, due to the comparable high concentrations needed for NMR spectroscopy, the low quantum yield of the reaction (*cf.* 3.4% in DMSO), and the increasingly yellow coloring of the sample. The latter can be ascribed to the known formation of nitroso and azo-species and lead to an inner filter effect, slowing down the conversion further. From the final NMR, we can conclude that the conversion can be driven to completion, however, the timescales required are rather long, pointing to the possibility of leftover **NO_2-NV** in solution at shorter irradiation times, even at significantly lower concentrations.

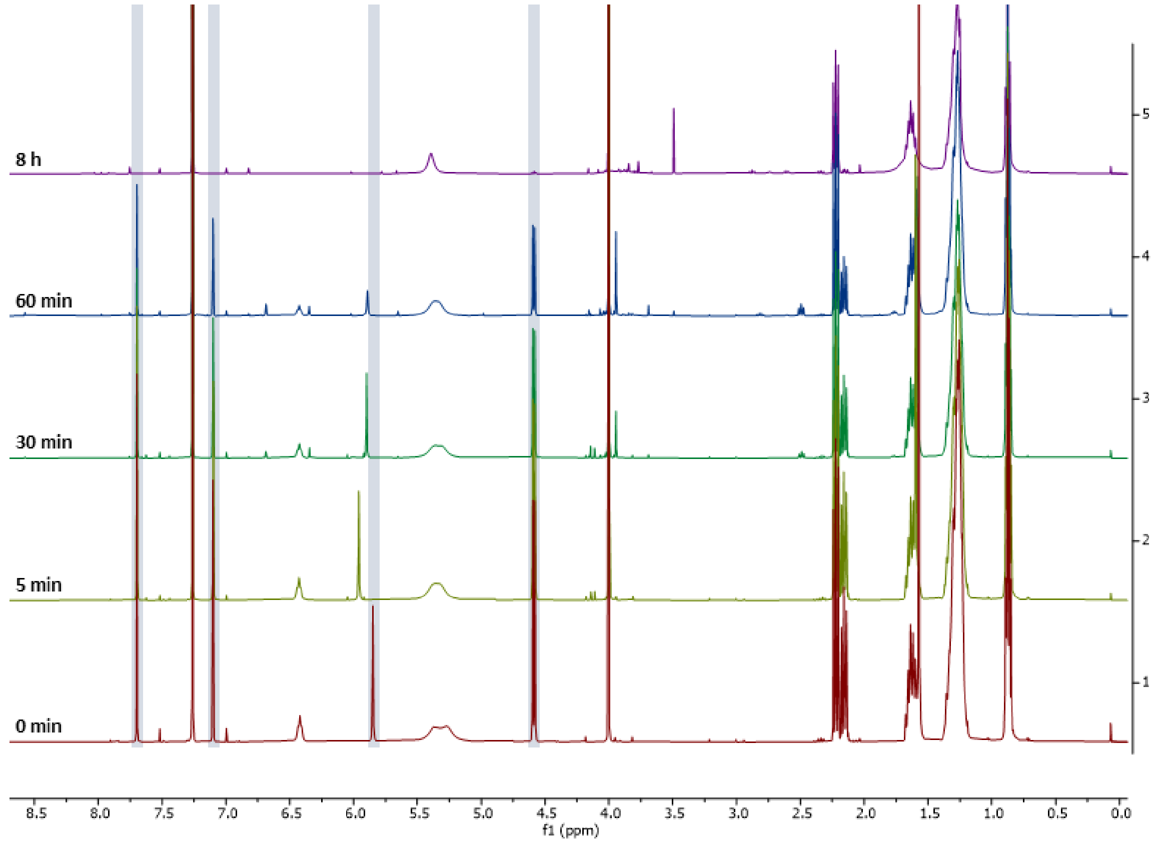

*Figure S31: NMR Spectra of **NO_2-NV** in $CDCl_3$ (ca. 1.5 mg/mL) under 365 nm irradiation.*

3.6. HPLC Trances of thePhotochemical Conversion of DEAC-NO_2-NV

HPLC-traces of **DEAC-NO_2-NV** before and after irradiation with 430 nm light (ThorLabs 430 nm LED, full power) in a transparent HPLC vial for *ca*. 30 min (recoded at 220 nm in a gradient of 5→95% MeCN in water with 0.1% formic acid).

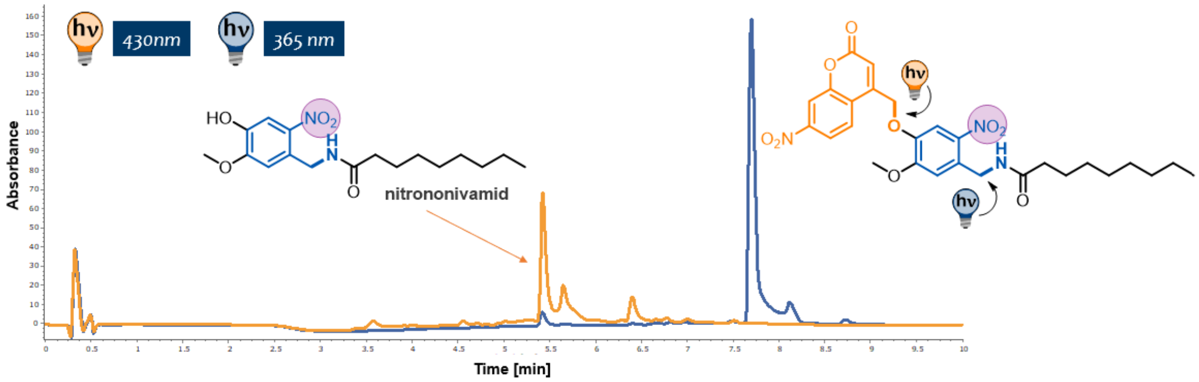

*Figure S32: HPLC traces of **DEAC-NO_2-NV** before (blue) and after (orange) 430 nm irradiation.*

4. Computational Chemistry

The tetrameric protein TRPV1 was obtained from the crystal structure PDB: 7LPE. Initial docking calculations were performed to identify potential ligand binding poses. Protein and ligand preparation were done using Autodock Tools,[6] and docking was carried out using Autodock Vina.[7,8] The binding pocket of a single monomer was used as the docking site, while the entire tetrameric assembly was retained for context. The two poses with the highest docking scores were selected to perform all-atom molecular dynamics (MD) simulations.

All simulations were performed with GROMACS 2020.4[9] using the CHARMM36m forcefield.[10] The lowest-energy pose was placed into the binding pocket of two monomers, while the second-lowest energy pose was positioned in the remaining two monomers. These starting configurations were used to build the full system in CHARMM-GUI,[11–13] embedding the tetrameric TRPV1 in a 12 × 12 nm^2 POPC membrane. The system was solvated using TIP3P water and neutralized with 0.15 M NaCl. The system setup is shown in Figure S34. Parameters for the ligands **CAP** and **NO_2-CAP** were obtained from CHARMM-GUI using the CHARMM General Force Field (CGENFF).[14]

Energy minimization was performed using 5000 steps of steepest descent. Equilibration was conducted in six steps, gradually releasing position restraints on the protein and the membrane, and increasing the timestep from Δt = 1 fs to 2 fs. The first three steps were run under NVT conditions for 125 ps each using the Berendsen thermostat (τ_T = 1 ps), followed by three equilibrations of 500 ps each under NPT conditions using the Berendsen thermostat (τ_T = 1 ps) and Berendsen barostat (τ_p = 5 ps), maintaining T = 303.15 K and p = 1 bar. Production runs were carried out for 250 ns, with a Δt = 2 fs timestep, and the temperature and pressure were kept with the Nosé-Hoover thermostat and the Parrinello-Rahman barostat (τ_p = 5 ps), respectively. Van der Waals interactions were treated with a cutoff scheme (1.2 nm); Coulomb interactions were calculated using PME (1.2 nm), following the recommended settings for the CHARMM force field.

For the analysis, the root-mean-square deviation (RMSD) of each ligand was computed relative to its minimized (docked) configuration. To ensure alignment and accurate RMSD measurements, each trajectory was fitted to the protein backbone (Cα atoms) within 5 Å of the ligand in the crystal structure. The selected alignment residues included SER512, MET547, ASN551, LEU553, TYR554, ALA566, and GLU570 from the same monomer as the ligand, and LEU662 from the adjacent chain.

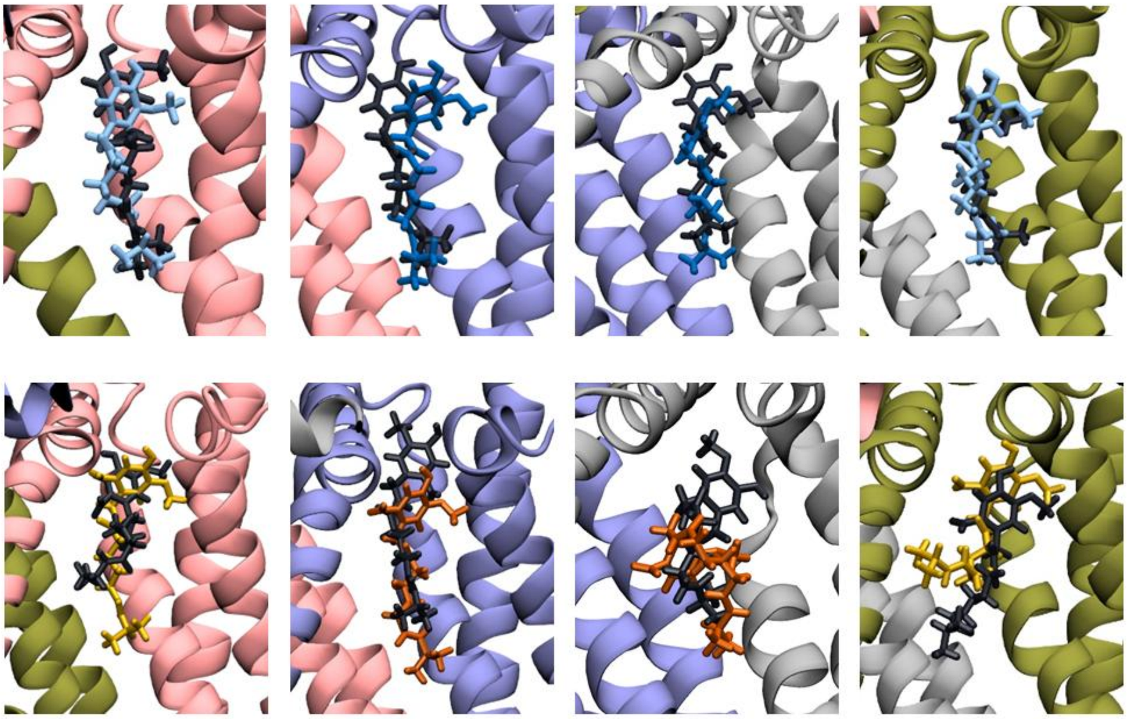

Figure S33. Snapshots of the tetrameric protein in each of the monomers (pink, ice blue, gray, dark green), initial minimized structures of the ligands (black) and the last configuration of the 250 ns simulation for **CAP** in blue (top) and **NO_2-CAP** in orange (bottom). The starting configuration of the ligands was pose 1 (dark blue and dark orange) and pose 2 (light blue and light orange).

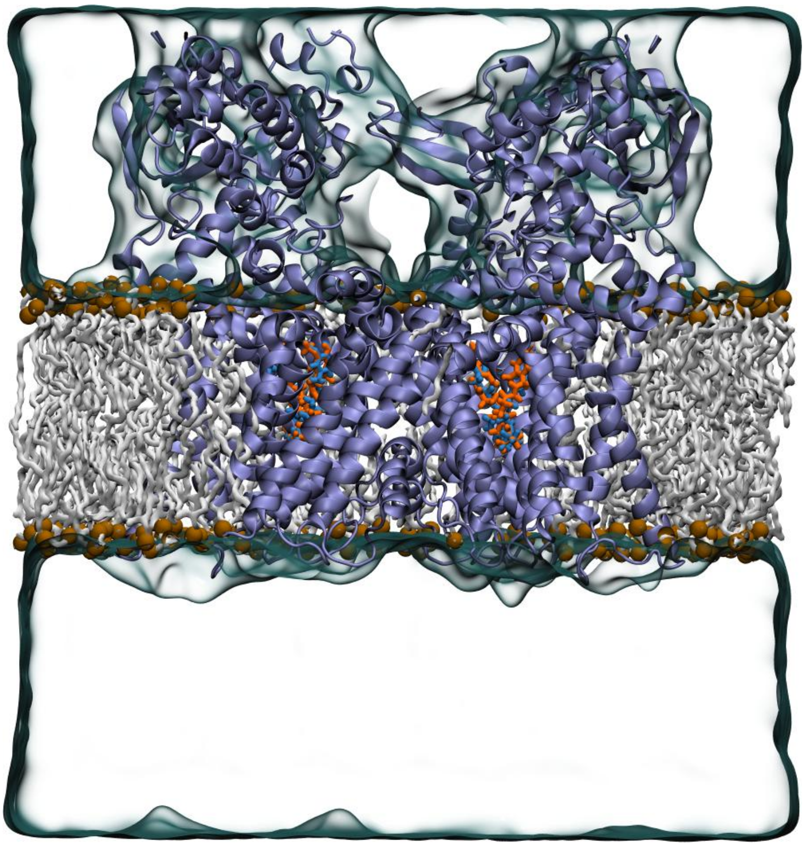

Figure S34. Snapshot of the tetrameric protein (iceblue) embedded in a POPC membrane (tails in light silver and polar heads in ochre). The initial configurations of the ligands from the two simulations are displayed together: **CAP** (blue) and **NO_2-CAP** (orange).

5. Biological Assays

Dissection and culture of dorsal root ganglion (DRG) and dorsal horn spinal neurons

Microdot (microisland) plates were prepared as previously described by Burgalossi et al., 2012.[15] Cortex astrocytes were then plated and maintained at 37°C with 5% CO_2 until neuronal dissection on DIV 2–3. The vertebral body was isolated by removing the skin and muscles, excising the internal organs, and severing it from the torso along both sides of the ribs. The vertebral body was then bisected along the spine, carefully avoiding damage to the spinal cord. Using straight forceps, the nerve fibers were gently detached from the ganglia by sweeping the tip of the forceps between the vertebral wall and spinal cord, from the cervical to the lumbar region. The spinal cord was then extracted by pinching the cervical region with straight forceps and pulling in a quick but careful motion from cervical to lumbar while lifting upwards. Dorsal root ganglia (DRGs) were carefully dissected from the intervertebral foramia using straight forceps. The spinal cord and DRG transferred to a separate dish containing Hanks' Balanced Salt Solution (HBSS), and then the nerve fibers attached to the DRG somata were subsequently removed.

The isolated tissues were then incubated in papain solution (Worthington Biochemical Corporation, 25 U/mL) for 1 hour at 37°C with gentle agitation (450 rpm) in a ThermoMixer. This was followed by a 15-minute incubation in a stop solution under the same conditions.
After enzymatic digestion, cells were dissociated in Neurobasal–A medium (Thermo Fisher Scientific) supplemented with 2% v/v B-27 supplement (Thermo Fisher Scientific), 2mM Glutamax (Thermo Fisher Scientific), and 0.1 % penicillin/streptomycin. The dissociated neurons were seeded onto astrocyte microisland cultures and maintained in Complete induced dorsal sensory neuron (idSN) Medium. This medium consisted of Basic idSN Medium (96% v/v Neurobasal–A medium, 2mM Glutamax, 2% v/v B-27 supplement, 1% v/v N2 supplement, 0.02 mM β-Mercaptoethanol and 0.1 % penicillin/streptomycin, all from Thermo Fisher Scientific), 0.01 µg/mL BDNF (Cell Guidance Systems, GFH1), 0.01 µg/mL GDNF (Cell Guidance Systems, GFH2), 0.01 µg/mL NT3 (Peprotech, 450-03), 0.01 µg/mL NGF (Peprotech, 450-01), 0.2 mM Ascorbic Acid (Sigma Aldrich, A4544) and 0.1 µg/mL laminin 521 (BioLamina, LN521) at 37°C and 5% CO2. DRG-dorsal horn (DH) co-cultures were established, along with autaptic cultures of DRG neurons.

All mice used in the current study were housed in the Max-Planck Institute for Multidisciplinary Sciences Animal Care Facility in Göttingen, and all procedures were approved by the Institutional Animal Care and Use Committee.

Electrophysiology

After breaking the glass coverslip containing autaptic or co-cultured neurons with diamond scriber, the resulting fragments were transferred to a recording chamber filled with extracellular solution (140 mM NaCl, 2.4 mM KCl, 10 mM HEPES, 10 mM glucose monohydrate, 4 mM $CaCl_2$, 4 mM MgCl2, 7.4 pH, 320 mOsm).

Whole-cell patch-clamp recordings were conducted on cultured neurons in voltage-clamp mode using a MultiClamp 700B amplifier (Molecular Devices), with signals digitized via an Axon Instruments interface (Molecular Devices) at a sampling rate of 10 kHz. For autaptic cultures, neurons were held at −70 mV, and excitatory postsynaptic currents (EPSCs)/inhibitory postsynaptic currents (IPSCs) were evoked by brief depolarization to 0 mV. The internal pipette solution contained (in mM): 136 KCl, 17.8 HEPES, 1 K-EGTA, 0.6 $MgCl_2$, 4 Mg-ATP, 0.3 Na-GTP, 15 creatine phosphate, and 5 U/mL creatine phosphokinase (pH 7.4, ~320 mOsm). Patch pipettes (2.5–4.5 MΩ) were pulled using a Sutter P-2000 horizontal puller. Series resistance was compensated by 35–80%, and only recordings with series resistance <10 MΩ were included in the analysis.

In co-culture preparations, the same stimulation protocol was applied to the presynaptic DRG neuron, while the patch pipette for the postsynaptic spinal cord neuron contained QX-314, a blocker of voltage-gated Na^+ channels, to suppress action potential propagation. This configuration ensured that EPSCs recorded in DH neurons reflected monosynaptic transmission from DRG neurons, not autaptic activity. To further ensure synaptic specificity, recordings were restricted to microislands containing only one presynaptic and one postsynaptic cell. All recordings were performed at DIV 9–16 and data are presented as mean ± standard error of the mean (SEM).

Electrophysiological data analysis

The analysis of the electrophysiological recordings was performed using AxoGraph X 1.5.4. Al recorded traces were preprocessed using 1 kHz filter. Graphs were made using GraphPad Prism.

Further Measurements

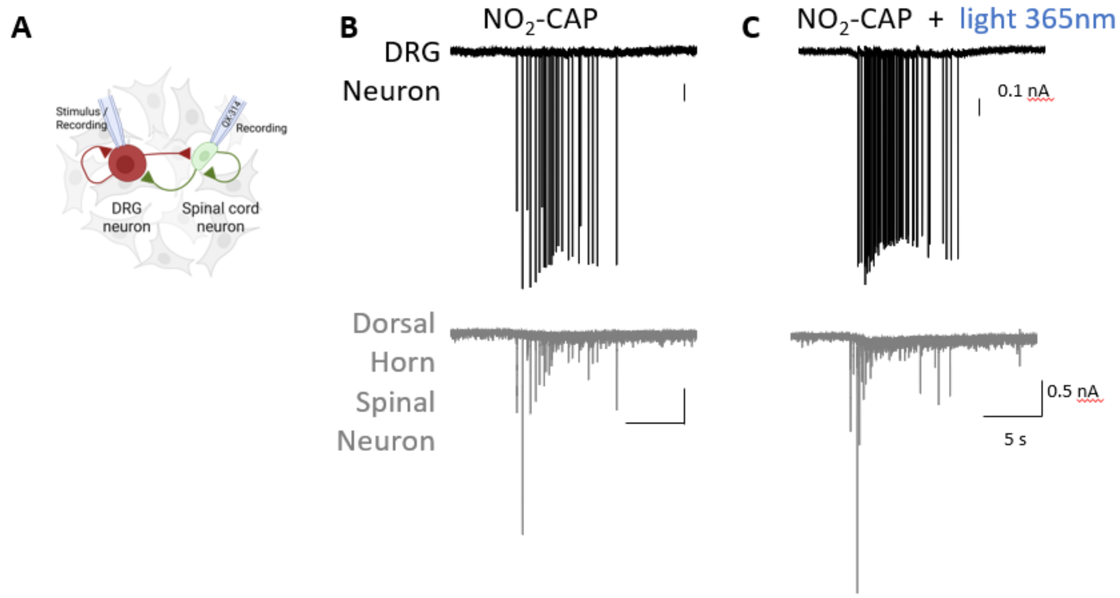

Figure S33. **Functional assessment of NO_2-CAP photosensitivity in a DRG–spinal cord neuron co-culture model.** A. Simultaneous dual whole-cell voltage-clamp recordings were performed from DRG and spinal cord neurons co-cultured on a single astrocyte microisland. To prevent action potential firing in the postsynaptic neuron and isolate presynaptic responses from DRG response, QX-314 was included in the spinal cord pipette solution. B and C. Local perfusion of **NO_2-CAP** induced fast spike currents recorded in both the DRG and the spinal cord neuron, suggesting synaptic transmission without (B) and with (C) illumination with 365 nm light.

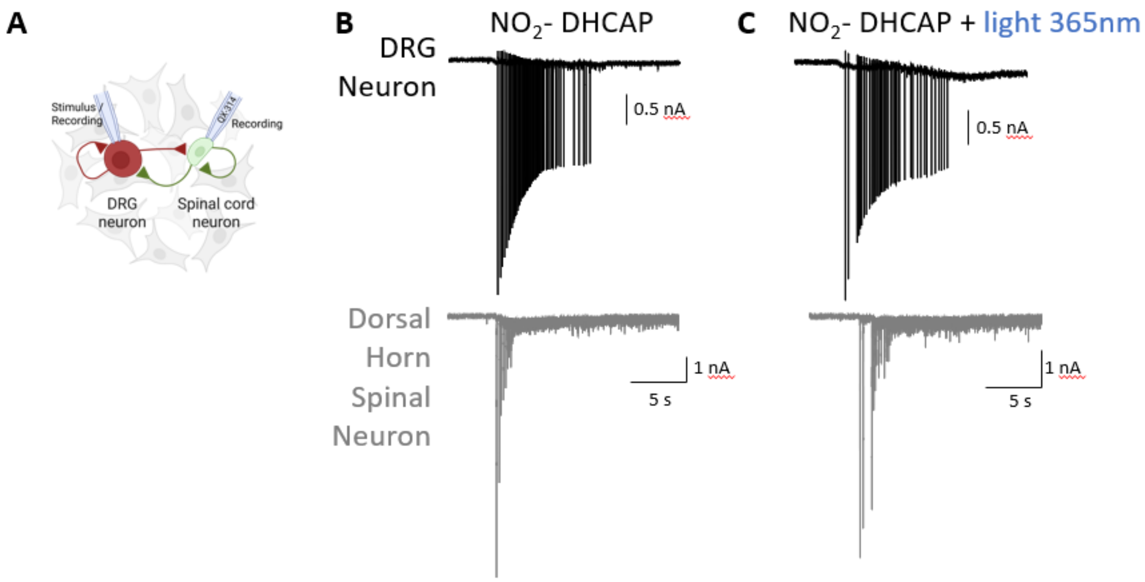

Figure S33. **Functional assessment of NO_2-DHCAP photosensitivity in a DRG–spinal cord neuron co-culture model.** A. Simultaneous dual whole-cell voltage-clamp recordings were performed from DRG and spinal cord neurons co-cultured on a single astrocyte microisland. To prevent action potential firing in the postsynaptic neuron and isolate presynaptic responses from DRG response, QX-314 was included in the spinal cord pipette solution. B and C. Local perfusion of **NO_2-DHCAP** induced fast spike currents recorded in both the DRG and the spinal cord neuron, suggesting synaptic transmission without (B) and with (C) illumination with 365 nm light.

6. X-Ray Analysis: Structure Tables

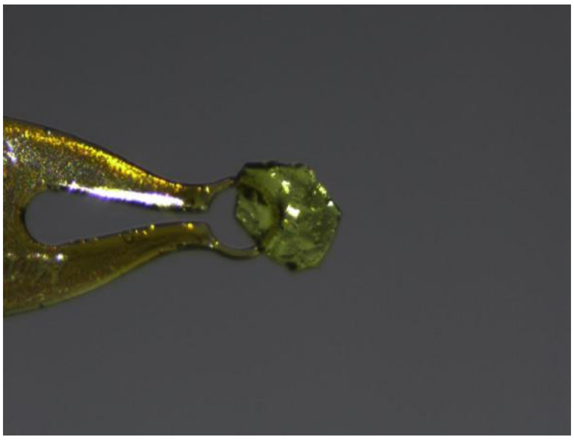

Crystallized from water:ethanol = 1:1. The data for 1829_p21c were collected from a shock-cooled single crystal at 200.00 K on a Bruker D8 VENTURE dual wavelength Mo/Cu four-circle diffractometer with a microfocus sealed X-ray tube using a mirror optics as monochromator and a Bruker PHOTON III detector. The diffractometer was equipped with an Oxford Cryostream 800 low temperature device and used MoK_α radiation (λ = 0.71073 Å). All data were integrated with SAINT and a multi-scan absorption correction using SADABS was applied.[16,17] The structure was solved by dual methods using XT and refined by full-matrix least-squares methods against F^2 by XL.[18,19] All non-hydrogen atoms were refined with anisotropic displacement parameters. All C-bound hydrogen atoms were refined with isotropic displacement parameters. Some were refined freely and some on calculated positions using a riding model with their U_{iso} values constrained to 1.5 times the U_{eq} of their pivot atoms for terminal sp^3 carbon atoms and 1.2 times for all other carbon atoms. Disordered moieties were refined using bond lengths restraints and displacement parameter restraints. Crystallographic data for the structures reported in this paper have been deposited with the Cambridge Crystallographic Data Centre.[20] CCDC (Deposition Number 2451419) contain the supplementary crystallographic data for this paper. These data can be obtained free of charge from The Cambridge Crystallographic Data Centre via www.ccdc.cam.ac.uk/ structures. This report and the CIF file were generated using FinalCif.[21]

Table 1. Crystal data and structure refinement for 1829_p21c

CCDC number	
Empirical formula	$C_{17}H_{26}N_2O_5$
Formula weight	338.40
Temperature [K]	200.00
Crystal system	monoclinic
Space group (number)	$P2_1/c$ (14)
a [Å]	14.3493(17)
b [Å]	10.0749(9)
c [Å]	14.3067(17)
α [°]	90
β [°]	117.666(3)
γ [°]	90
Volume [Å^3]	1831.8(4)
Z	4
ρ_{calc} [gcm^{-3}]	1.227
μ [mm^{-1}]	0.090
F(000)	728
Crystal size [mm^3]	0.349×0.347×0.046
Crystal colour	yellow
Crystal shape	plate
Radiation	MoK_α (λ=0.71073 Å)
2θ range [°]	5.16 to 57.47 (0.74 Å)
Index ranges	−19 ≤ h ≤ 19<br>−13 ≤ k ≤ 13<br>−19 ≤ l ≤ 19
Reflections collected	59804
Independent reflections	4737<br>R_{int} = 0.0347<br>R_{sigma} = 0.0152
Completeness to θ = 25.242°	99.6 %
Data / Restraints / Parameters	4737/146/333
Goodness-of-fit on F^2	1.072
Final *R* indexes [I≥2σ(I)]	R_1 = 0.0482<br>wR_2 = 0.1396
Final *R* indexes [all data]	R_1 = 0.0599<br>wR_2 = 0.1495
Largest peak/hole [eÅ^{-3}]	0.24/-0.22

Table 2. Atomic coordinates and U_{eq} [Å^2] for 1829_p21c

Atom	*x*	*y*	*z*	U_{eq}
O2	0.44307(8)	0.14395(9)	0.49814(7)	0.0436(2)
O1	0.59443(9)	0.26676(10)	0.65567(7)	0.0501(3)
O4	0.64236(9)	0.58422(11)	0.33383(8)	0.0599(3)
O5	0.27559(9)	0.56040(11)	0.20551(8)	0.0583(3)
O3	0.70217(10)	0.63337(11)	0.49776(9)	0.0600(3)
N2	0.64837(9)	0.56362(11)	0.42117(9)	0.0441(3)
N1	0.35825(10)	0.39864(11)	0.16459(8)	0.0464(3)
H1A	0.350680	0.326209	0.127349	0.056
C2	0.48778(10)	0.24581(11)	0.47163(9)	0.0366(3)
C3	0.45617(10)	0.29069(12)	0.36943(9)	0.0384(3)
H3	0.398888	0.247812	0.312607	0.046
C4	0.50551(10)	0.39634(12)	0.34736(9)	0.0372(3)
C5	0.59018(10)	0.45362(11)	0.43410(10)	0.0375(3)
C1	0.57067(10)	0.30955(12)	0.55727(9)	0.0383(3)
C6	0.62221(10)	0.41232(12)	0.53750(9)	0.0388(3)
H6	0.679584	0.455086	0.594301	0.047
C7	0.46375(11)	0.44469(13)	0.23373(10)	0.0441(3)
H7A	0.464241	0.542944	0.233328	0.053
H7B	0.511575	0.413703	0.205921	0.053
C9	0.27227(12)	0.46037(14)	0.15458(10)	0.0487(3)
C8	0.35676(12)	0.07574(14)	0.41548(11)	0.0493(3)
H8A	0.379241	0.038368	0.365938	0.074
H8B	0.298739	0.138100	0.378107	0.074
H8C	0.333158	0.004014	0.445726	0.074
C10	0.16782(14)	0.40205(19)	0.07615(13)	0.0658(5)
H10C	0.178777	0.309611	0.059891	0.079
H10D	0.139766	0.453709	0.009753	0.079
H10A	0.176924	0.351635	0.021543	0.079
H10B	0.116765	0.474412	0.040753	0.079
C11	0.08727(16)	0.4029(2)	0.11812(18)	0.0671(5)
H11A	0.018274	0.373614	0.061359	0.081
H11B	0.079082	0.494836	0.137650	0.081
C12	0.1191(2)	0.3122(3)	0.2148(2)	0.0912(8)

H12A	0.142492	0.226007	0.199804	0.109
H12B	0.180077	0.352695	0.275406	0.109
H12C	0.196043	0.317421	0.260582	0.109
H12D	0.100821	0.219063	0.191383	0.109
C13A	0.0347(6)	0.2863(7)	0.2465(6)	0.118(2)
H13A	-0.028328	0.250896	0.185037	0.141
H13B	0.059676	0.217701	0.302462	0.141
C16A	-0.0861(4)	0.3228(6)	0.3930(5)	0.0969(14)
H16A	-0.013924	0.349174	0.444812	0.116
H16B	-0.085216	0.225995	0.381506	0.116
C15A	-0.1128(6)	0.3905(9)	0.2930(6)	0.123(3)
H15A	-0.144139	0.478495	0.291414	0.148
H15B	-0.163803	0.337265	0.232827	0.148
C14A	0.0043(8)	0.4079(10)	0.2862(7)	0.146(3)
H14A	-0.002584	0.483222	0.238995	0.175
H14B	0.061294	0.429605	0.357317	0.175
C17A	-0.1569(10)	0.3487(15)	0.4398(8)	0.104(3)
H17A	-0.132339	0.299091	0.505930	0.157
H17B	-0.156931	0.443846	0.453933	0.157
H17C	-0.228426	0.320315	0.390446	0.157
H1	0.6378(17)	0.322(2)	0.7018(17)	0.078(6)
C13B	0.0588(5)	0.3577(15)	0.2796(7)	0.106(4)
H13C	0.090517	0.310852	0.348246	0.128
H13D	0.070899	0.453884	0.294344	0.128
C14B	-0.0606(5)	0.3320(10)	0.2245(6)	0.097(2)
H14C	-0.101730	0.409416	0.183403	0.117
H14D	-0.080502	0.251731	0.179428	0.117
C15B	-0.0684(8)	0.3118(9)	0.3256(8)	0.112(3)
H15C	-0.117527	0.236688	0.312022	0.135
H15D	0.001676	0.280015	0.378627	0.135
C17B	-0.1656(18)	0.370(3)	0.428(2)	0.161(10)
H17D	-0.180510	0.447936	0.460370	0.241
H17E	-0.231880	0.332323	0.374788	0.241
H17F	-0.127755	0.303734	0.482876	0.241
C16B	-0.0989(13)	0.4114(13)	0.3772(13)	0.150(5)

H16C	-0.137615	0.481223	0.324639	0.180
H16D	-0.033974	0.452766	0.432224	0.180
C15C	-0.0200(10)	0.4091(14)	0.3762(12)	0.075(3)
H15E	-0.012226	0.506618	0.374359	0.090
H15F	0.015809	0.378606	0.450453	0.090
C11C	0.1252(15)	0.3093(18)	0.1331(16)	0.109(5)
H11C	0.066512	0.254928	0.081196	0.130
H11D	0.181450	0.248924	0.181415	0.130
C12C	0.0896(15)	0.391(2)	0.1910(14)	0.097(5)
H12E	0.088098	0.481514	0.163303	0.116
H12F	0.148602	0.391056	0.263620	0.116
C16C	-0.1401(12)	0.367(2)	0.3197(19)	0.085(6)
H16E	-0.182265	0.434106	0.266360	0.102
H16F	-0.148254	0.280774	0.283149	0.102
C13C	-0.0035(16)	0.385(3)	0.2053(15)	0.117(6)
H13E	-0.054793	0.322766	0.152969	0.140
H13F	-0.036469	0.473606	0.193063	0.140
C14C	0.0224(15)	0.3400(17)	0.3114(14)	0.086(5)
H14E	0.100102	0.341185	0.352883	0.103
H14F	0.000460	0.245941	0.305681	0.103
C17C	-0.179(3)	0.354(3)	0.401(3)	0.086(7)
H17G	-0.253375	0.327990	0.365851	0.130
H17H	-0.137674	0.286768	0.452997	0.130
H17I	-0.171604	0.439730	0.436248	0.130

U_{eq} is defined as 1/3 of the trace of the orthogonalized U_{ij} tensor.

Table 3. Anisotropic displacement parameters [Å^2] for 1829_p21c.
The anisotropic displacement factor exponent takes the form:
$-2\pi^2[\, h^2(a^*)^2U_{11} + k^2(b^*)^2U_{22} + \ldots + 2hka^*b^*U_{12}\,]$

**Atom**	U_{11}	U_{22}	U_{33}	U_{23}	U_{13}	U_{12}
O2	0.0585(5)	0.0375(5)	0.0345(4)	−0.0014(3)	0.0212(4)	−0.0063(4)
O1	0.0711(7)	0.0455(5)	0.0290(4)	−0.0001(4)	0.0193(4)	−0.0097(5)
O4	0.0698(7)	0.0613(7)	0.0495(6)	0.0174(5)	0.0284(5)	−0.0037(5)
O5	0.0592(6)	0.0536(6)	0.0501(6)	−0.0161(5)	0.0153(5)	0.0063(5)
O3	0.0747(7)	0.0479(6)	0.0634(7)	−0.0095(5)	0.0372(6)	−0.0149(5)

N2	0.0508(6)	0.0352(5)	0.0490(6)	0.0049(5)	0.0253(5)	0.0050(4)
N1	0.0639(7)	0.0390(6)	0.0306(5)	−0.0013(4)	0.0170(5)	0.0082(5)
C2	0.0488(7)	0.0304(5)	0.0332(6)	−0.0008(4)	0.0213(5)	0.0034(5)
C3	0.0492(7)	0.0350(6)	0.0303(5)	−0.0026(4)	0.0177(5)	0.0039(5)
C4	0.0488(6)	0.0334(6)	0.0317(5)	0.0014(4)	0.0207(5)	0.0094(5)
C5	0.0469(6)	0.0308(5)	0.0386(6)	0.0020(4)	0.0231(5)	0.0060(5)
C1	0.0517(7)	0.0338(6)	0.0300(6)	0.0002(4)	0.0197(5)	0.0051(5)
C6	0.0466(6)	0.0347(6)	0.0338(6)	−0.0020(5)	0.0176(5)	0.0028(5)
C7	0.0589(8)	0.0422(7)	0.0329(6)	0.0055(5)	0.0227(6)	0.0084(6)
C9	0.0606(8)	0.0430(7)	0.0318(6)	−0.0008(5)	0.0124(6)	0.0068(6)
C8	0.0600(8)	0.0443(7)	0.0420(7)	−0.0048(5)	0.0223(6)	−0.0096(6)
C10	0.0637(10)	0.0625(10)	0.0460(8)	−0.0108(7)	0.0041(7)	0.0048(7)
C11	0.0561(10)	0.0561(11)	0.0639(12)	0.0006(9)	0.0066(9)	0.0029(8)
C12	0.0837(17)	0.0792(17)	0.0843(18)	0.0225(14)	0.0167(13)	−0.0078(14)
C13A	0.109(5)	0.124(5)	0.101(4)	0.015(4)	0.032(4)	−0.049(4)
C16A	0.088(3)	0.076(3)	0.123(4)	0.006(3)	0.046(3)	0.028(2)
C15A	0.117(5)	0.131(6)	0.138(6)	0.034(5)	0.074(5)	0.060(4)
C14A	0.171(8)	0.163(7)	0.122(6)	0.022(5)	0.083(6)	−0.004(7)
C17A	0.100(6)	0.107(6)	0.099(5)	0.000(4)	0.039(4)	0.048(5)
C13B	0.053(4)	0.174(11)	0.069(5)	0.051(6)	0.008(3)	−0.030(5)
C14B	0.066(4)	0.120(6)	0.082(4)	0.014(4)	0.013(3)	−0.025(4)
C15B	0.125(7)	0.092(6)	0.146(8)	0.054(6)	0.085(7)	0.035(6)
C17B	0.050(7)	0.19(2)	0.22(2)	−0.005(14)	0.045(10)	0.017(10)
C16B	0.128(10)	0.087(7)	0.243(17)	0.008(9)	0.092(12)	0.025(7)
C15C	0.063(6)	0.049(7)	0.092(8)	−0.017(6)	0.019(6)	0.000(5)
C11C	0.116(13)	0.113(10)	0.082(10)	−0.005(8)	0.032(9)	−0.028(9)
C12C	0.097(10)	0.103(12)	0.070(10)	0.005(9)	0.022(8)	−0.027(9)
C16C	0.068(7)	0.065(10)	0.100(12)	−0.047(10)	0.021(7)	−0.010(7)
C13C	0.112(11)	0.120(17)	0.111(10)	0.024(10)	0.046(9)	−0.019(10)
C14C	0.103(11)	0.049(8)	0.100(10)	0.006(7)	0.042(9)	0.026(8)
C17C	0.078(15)	0.050(10)	0.124(17)	−0.045(12)	0.040(13)	−0.013(10)

Table 4. Bond lengths and angles for 1829_p21c

Atom–Atom	Length [Å]
O2–C2	1.3545(15)
O2–C8	1.4299(16)
O1–C1	1.3558(14)
O1–H1	0.87(2)
O4–N2	1.2296(15)
O5–C9	1.2314(17)
O3–N2	1.2268(16)
N2–C5	1.4502(16)
N1–H1A	0.8800
N1–C7	1.4473(19)
N1–C9	1.3288(19)
C2–C3	1.3900(16)
C2–C1	1.4053(17)
C3–H3	0.9500
C3–C4	1.3933(18)
C4–C5	1.3961(18)
C4–C7	1.5280(16)
C5–C6	1.3933(17)
C1–C6	1.3760(18)
C6–H6	0.9500
C7–H7A	0.9900
C7–H7B	0.9900
C9–C10	1.513(2)
C8–H8A	0.9800
C8–H8B	0.9800
C8–H8C	0.9800
C10–H10C	0.9900
C10–H10D	0.9900
C10–H10A	0.9900
C10–H10B	0.9900
C10–C11	1.530(3)
C10–C11C	1.541(14)
C11–H11A	0.9900
C11–H11B	0.9900
C11–C12	1.539(3)
C12–H12A	0.9900
C12–H12B	0.9900
C12–H12C	0.9900
C12–H12D	0.9900
C12–C13A	1.501(7)
C12–C13B	1.600(11)
C13A–H13A	0.9900
C13A–H13B	0.9900
C13A–C14A	1.497(10)
C16A–H16A	0.9900
C16A–H16B	0.9900
C16A–C15A	1.466(8)
C16A–C17A	1.476(9)
C15A–H15A	0.9900
C15A–H15B	0.9900
C15A–C14A	1.736(10)
C14A–H14A	0.9900
C14A–H14B	0.9900
C17A–H17A	0.9800
C17A–H17B	0.9800
C17A–H17C	0.9800
C13B–H13C	0.9900
C13B–H13D	0.9900
C13B–C14B	1.541(9)
C14B–H14C	0.9900
C14B–H14D	0.9900
C14B–C15B	1.515(10)
C15B–H15C	0.9900
C15B–H15D	0.9900
C15B–C16B	1.430(12)
C17B–H17D	0.9800
C17B–H17E	0.9800

C17B–H17F	0.9800
C17B–C16B	1.507(16)
C16B–H16C	0.9900
C16B–H16D	0.9900
C15C–H15E	0.9900
C15C–H15F	0.9900
C15C–C16C	1.586(15)
C15C–C14C	1.496(14)
C11C–H11C	0.9900
C11C–H11D	0.9900
C11C–C12C	1.421(16)
C12C–H12E	0.9900
C12C–H12F	0.9900
C12C–C13C	1.443(16)
C16C–H16E	0.9900
C16C–H16F	0.9900
C16C–C17C	1.509(18)
C13C–H13E	0.9900
C13C–H13F	0.9900
C13C–C14C	1.457(16)
C14C–H14E	0.9900
C14C–H14F	0.9900
C17C–H17G	0.9800
C17C–H17H	0.9800
C17C–H17I	0.9800
**Atom–Atom–Atom**	**Angle [°]**
C2–O2–C8	118.26(10)
C1–O1–H1	109.4(14)
O4–N2–C5	119.06(12)
O3–N2–O4	121.95(12)
O3–N2–C5	118.99(11)
C7–N1–H1A	118.4
C9–N1–H1A	118.4

C9–N1–C7	123.22(11)
O2–C2–C3	125.13(11)
O2–C2–C1	114.91(10)
C3–C2–C1	119.95(11)
C2–C3–H3	118.8
C2–C3–C4	122.33(11)
C4–C3–H3	118.8
C3–C4–C5	116.01(11)
C3–C4–C7	119.67(11)
C5–C4–C7	124.29(11)
C4–C5–N2	121.17(11)
C6–C5–N2	115.98(11)
C6–C5–C4	122.83(11)
O1–C1–C2	117.58(11)
O1–C1–C6	123.55(11)
C6–C1–C2	118.87(11)
C5–C6–H6	120.0
C1–C6–C5	119.94(11)
C1–C6–H6	120.0
N1–C7–C4	113.20(11)
N1–C7–H7A	108.9
N1–C7–H7B	108.9
C4–C7–H7A	108.9
C4–C7–H7B	108.9
H7A–C7–H7B	107.8
O5–C9–N1	122.71(14)
O5–C9–C10	120.64(14)
N1–C9–C10	116.65(13)
O2–C8–H8A	109.5
O2–C8–H8B	109.5
O2–C8–H8C	109.5
H8A–C8–H8B	109.5
H8A–C8–H8C	109.5
H8B–C8–H8C	109.5
C9–C10–H10C	109.2

C9–C10–H10D	109.2
C9–C10–H10A	109.7
C9–C10–H10B	109.7
C9–C10–C11	112.05(14)
C9–C10–C11C	110.1(8)
H10C–C10–H10D	107.9
H10A–C10–H10B	108.2
C11–C10–H10C	109.2
C11–C10–H10D	109.2
C11C–C10–H10A	109.7
C11C–C10–H10B	109.7
C10–C11–H11A	109.0
C10–C11–H11B	109.0
C10–C11–C12	112.72(19)
H11A–C11–H11B	107.8
C12–C11–H11A	109.0
C12–C11–H11B	109.0
C11–C12–H12A	108.4
C11–C12–H12B	108.4
C11–C12–H12C	109.7
C11–C12–H12D	109.7
C11–C12–C13B	109.6(4)
H12A–C12–H12B	107.5
H12C–C12–H12D	108.2
C13A–C12–C11	115.5(4)
C13A–C12–H12A	108.4
C13A–C12–H12B	108.4
C13B–C12–H12C	109.7
C13B–C12–H12D	109.7
C12–C13A–H13A	109.0
C12–C13A–H13B	109.0
H13A–C13A–H13B	107.8
C14A–C13A–C12	113.0(6)

C14A–C13A–H13A	109.0
C14A–C13A–H13B	109.0
H16A–C16A–H16B	107.4
C15A–C16A–H16A	108.3
C15A–C16A–H16B	108.3
C15A–C16A–C17A	115.9(6)
C17A–C16A–H16A	108.3
C17A–C16A–H16B	108.3
C16A–C15A–H15A	110.5
C16A–C15A–H15B	110.5
C16A–C15A–C14A	106.3(6)
H15A–C15A–H15B	108.7
C14A–C15A–H15A	110.5
C14A–C15A–H15B	110.5
C13A–C14A–C15A	113.5(7)
C13A–C14A–H14A	108.9
C13A–C14A–H14B	108.9
C15A–C14A–H14A	108.9

C15A–C14A–H14B	108.9
H14A–C14A–H14B	107.7
C16A–C17A–H17A	109.5
C16A–C17A–H17B	109.5
C16A–C17A–H17C	109.5
H17A–C17A–H17B	109.5
H17A–C17A–H17C	109.5
H17B–C17A–H17C	109.5
C12–C13B–H13C	108.4
C12–C13B–H13D	108.4
H13C–C13B–H13D	107.5
C14B–C13B–C12	115.3(8)
C14B–C13B–H13C	108.4
C14B–C13B–H13D	108.4
C13B–C14B–H14C	112.7
C13B–C14B–H14D	112.7
H14C–C14B–H14D	110.2
C15B–C14B–C13B	95.3(6)
C15B–C14B–H14C	112.7

C15B–C14B–H14D	112.7
C14B–C15B–H15C	106.1
C14B–C15B–H15D	106.1
H15C–C15B–H15D	106.3
C16B–C15B–C14B	125.0(9)
C16B–C15B–H15C	106.1
C16B–C15B–H15D	106.1
H17D–C17B–H17E	109.5
H17D–C17B–H17F	109.5
H17E–C17B–H17F	109.5
C16B–C17B–H17D	109.5
C16B–C17B–H17E	109.5
C16B–C17B–H17F	109.5
C15B–C16B–C17B	118.2(13)
C15B–C16B–H16C	107.8
C15B–C16B–H16D	107.8
C17B–C16B–H16C	107.8
C17B–C16B–H16D	107.8

H16C–C16B–H16D	107.1
H15E–C15C–H15F	109.2
C16C–C15C–H15E	111.3
C16C–C15C–H15F	111.3
C14C–C15C–H15E	111.3
C14C–C15C–H15F	111.3
C14C–C15C–C16C	102.6(11)
C10–C11C–H11C	110.3
C10–C11C–H11D	110.3
H11C–C11C–H11D	108.5
C12C–C11C–C10	107.1(13)
C12C–C11C–H11C	110.3
C12C–C11C–H11D	110.3
C11C–C12C–H12E	104.3
C11C–C12C–H12F	104.3
C11C–C12C–C13C	131.6(17)
H12E–C12C–H12F	105.6
C13C–C12C–H12E	104.3
C13C–C12C–H12F	104.3

C15C–C16C–H16E	109.8
C15C–C16C–H16F	109.8
H16E–C16C–H16F	108.2
C17C–C16C–C15C	109.5(15)
C17C–C16C–H16E	109.8
C17C–C16C–H16F	109.8
C12C–C13C–H13E	109.5
C12C–C13C–H13F	109.5
C12C–C13C–C14C	110.9(15)
H13E–C13C–H13F	108.1
C14C–C13C–H13E	109.5
C14C–C13C–H13F	109.5
C15C–C14C–H14E	107.0
C15C–C14C–H14F	107.0
C13C–C14C–C15C	121.4(14)
C13C–C14C–H14E	107.0
C13C–C14C–H14F	107.0
H14E–C14C–H14F	106.7

C16C–C17C–H17G	109.5
C16C–C17C–H17H	109.5
C16C–C17C–H17I	109.5
H17G–C17C–H17H	109.5
H17G–C17C–H17I	109.5
H17H–C17C–H17I	109.5

Table 5. Torsion angles for 1829_p21c

Atom–Atom–Atom–Atom	Torsion Angle [°]
O2–C2–C3–C4	179.93(11)
O2–C2–C1–O1	−2.28(16)
O2–C2–C1–C6	178.59(11)
O1–C1–C6–C5	−177.48(11)
O4–N2–C5–C4	−19.20(18)
O4–N2–C5–C6	162.53(12)
O5–C9–C10–C11	−41.3(2)
O5–C9–C10–C11C	−84.7(8)
O3–N2–C5–C4	160.82(12)
O3–N2–C5–C6	−17.45(17)
N2–C5–C6–C1	179.27(11)
N1–C9–C10–C11	138.96(15)
N1–C9–C10–C11C	95.6(8)
C2–C3–C4–C5	1.02(17)
C2–C3–C4–C7	−177.31(11)
C2–C1–C6–C5	1.60(18)
C3–C2–C1–O1	176.30(11)
C3–C2–C1–C6	−2.83(18)
C3–C4–C5–N2	179.54(10)
C3–C4–C5–C6	−2.31(17)
C3–C4–C7–N1	16.59(16)
C4–C5–C6–C1	1.03(18)
C5–C4–C7–N1	−161.58(11)
C1–C2–C3–C4	1.51(18)
C7–N1–C9–O5	−1.8(2)
C7–N1–C9–C10	177.96(13)
C7–C4–C5–N2	−2.23(18)
C7–C4–C5–C6	175.93(11)
C9–N1–C7–C4	85.05(15)
C9–C10–C11–C12	−65.2(2)
C9–C10–C11C–C12C	73.3(16)
C8–O2–C2–C3	0.28(18)
C8–O2–C2–C1	178.78(11)
C10–C11–C12–C13A	−168.0(4)
C10–C11–C12–C13B	158.6(5)
C10–C11C–C12C–C13C	137(2)
C11–C12–C13A–C14A	−66.5(7)
C11–C12–C13B–C14B	69.5(9)
C12–C13A–C14A–C15A	165.3(5)
C12–C13B–C14B–C15B	149.3(8)
C16A–C15A–C14A–C13A	82.6(9)
C17A–C16A–C15A–C14A	153.5(9)
C13B–C14B–C15B–C16B	97.1(14)
C14B–C15B–C16B–C17B	142.6(16)
C11C–C12C–C13C–C14C	107(3)

C12C–C13C–C14C–C15C	133.7(19)
C16C–C15C–C14C–C13C	72(2)
C14C–C15C–C16C–C17C	146.5(19)

7. References

1 L. M. Hagge, A. Shahrivarkevishahi, N. M. Al-Kharji, Z. Chen, O. R. Brohlin, I. Trashi, A. Tumac, F. C. Herbert, A. V. Adlooru, H. Lee, H. R. Firouzi, S. A. Cornelius, N. J. De Nisco and J. J. Gassensmith, *J. Mater. Chem. B*, 2023, **11**, 7126–7133.
2 A. K. Migglautsch, M. Willim, B. Schweda, A. Glieder, R. Breinbauer and M. Winkler, *Tetrahedron*, 2018, **74**, 6199–6204.
3 M. Anderson, S. Afewerki, P. Berglund and A. Córdova, *Adv Synth Catal*, 2014, **356**, 2113–2118.
4 N. Mougios, E. R. Cotroneo, N. Imse, J. Setzke, S. O. Rizzoli, N. A. Simeth, R. Tsukanov and F. Opazo, *Nat Commun*, 2024, **15**, 8771.
5 K. Stranius and K. Börjesson, *Scientific Reports*, 2017, **7**, 1–9.
6 G. M. Morris, R. Huey, W. Lindstrom, M. F. Sanner, R. K. Belew, D. S. Goodsell and A. J. Olson, *J Comput Chem*, 2009, **30**, 2785–2791.
7 J. Eberhardt, D. Santos-Martins, A. F. Tillack and S. Forli, *J. Chem. Inf. Model.*, 2021, **61**, 3891–3898.
8 O. Trott and A. J. Olson, *J Comput Chem*, 2010, **31**, 455–461.
9 M. J. Abraham, T. Murtola, R. Schulz, S. Páll, J. C. Smith, B. Hess and E. Lindahl, *SoftwareX*, 2015, **1–2**, 19–25.
10 J. Huang and A. D. MacKerell, *J. Comput. Chem.*, 2013, **34**, 2135–2145.
11 S. Jo, T. Kim, V. G. Iyer and W. Im, *J Comput Chem*, 2008, **29**, 1859–1865.
12 J. Lee, X. Cheng, J. M. Swails, M. S. Yeom, P. K. Eastman, J. A. Lemkul, S. Wei, J. Buckner, J. C. Jeong, Y. Qi, S. Jo, V. S. Pande, D. A. Case, C. L. Brooks, A. D. MacKerell, J. B. Klauda and W. Im, *J. Chem. Theory Comput.*, 2016, **12**, 405–413.
13 S. Jo, T. Kim and W. Im, *PLoS ONE*, 2007, **2**, e880.
14 K. Vanommeslaeghe, E. Hatcher, C. Acharya, S. Kundu, S. Zhong, J. Shim, E. Darian, O. Guvench, P. Lopes, I. Vorobyov and A. D. Mackerell, *J Comput Chem*, 2010, **31**, 671–690.
15 A. Burgalossi, S. Jung, K. M. Man, R. Nair, W. J. Jockusch, S. M. Wojcik, N. Brose and J.-S. Rhee, *Nat Protoc*, 2012, **7**, 1351–1365.
16 Bruker, SAINT, V8.40A, Bruker AXS Inc., Madison, Wisconsin, USA.
17 L. Krause, R. Herbst-Irmer, G. M. Sheldrick and D. Stalke, *J Appl Crystallogr*, 2015, **48**, 3–10.
18 G. M. Sheldrick, *Acta Crystallogr A Found Adv*, 2015, **71**, 3–8.
19 G. M. Sheldrick, *Acta Crystallogr C Struct Chem*, 2015, **71**, 3–8.
20 C. R. Groom, I. J. Bruno, M. P. Lightfoot and S. C. Ward, *Acta Crystallogr B Struct Sci Cryst Eng Mater*, 2016, **72**, 171–179.
21 D. Kratzert, FinalCif, V113, https://dkratzert.de/finalcif.html.
